# Effective detection of rare variants in pooled DNA samples using Cross-pool tailcurve analysis

**DOI:** 10.1186/gb-2011-12-9-r93

**Published:** 2011-09-28

**Authors:** Tejasvi S Niranjan, Abby Adamczyk, Héctor Corrada Bravo, Margaret A Taub, Sarah J Wheelan, Rafael Irizarry, Tao Wang

**Affiliations:** 1 McKusick-Nathans Institute of Genetic Medicine and Department of Pediatrics, Johns Hopkins University School of Medicine, Baltimore, MD 21205, USA; 2 Predoctoral Training Program in Human Genetics, Johns Hopkins University School of Medicine, Baltimore, MD 21205, USA; 3 Center for Bioinformatics and Computational Biology, Department of Computer Science, University of Maryland, College Park, MD 20742, USA; 4 Current address: Center for Bioinformatics and Computational Biology, Department of Computer Science, University of Maryland, College Park, MD 20742, USA; 5 Department of Biostatistics, Bloomberg School of Public Health, Johns Hopkins University, Baltimore, MD 21205, USA; 6 Department of Oncology, Johns Hopkins University School of Medicine, Baltimore, MD 21287, USA

## Abstract

Sequencing targeted DNA regions in large samples is necessary to discover the full spectrum of rare variants. We report an effective Illumina sequencing strategy utilizing pooled samples with novel quality (Srfim) and filtering (*SERVIC^4^E*) algorithms. We sequenced 24 exons in two cohorts of 480 samples each, identifying 47 coding variants, including 30 present once per cohort. Validation by Sanger sequencing revealed an excellent combination of sensitivity and specificity for variant detection in pooled samples of both cohorts as compared to publicly available algorithms.

## Background

Next-generation sequencing and computational genomic tools permit rapid, deep sequencing for hundreds to thousands of samples [1-3]. Recently, rare variants of large effect have been recognized as conferring substantial risks for common diseases and complex traits in humans [4]. There is considerable interest in sequencing limited genomic regions such as sets of candidate genes and target regions identified by linkage and/or association studies. Sequencing large sample cohorts is essential to discover the full spectrum of genetic variants and provide sufficient power to detect differences in the allele frequencies between cases and controls. However, several technical and analytical challenges must be resolved to efficiently apply next-generation sequencing to large samples in individual laboratories. First, it remains expensive to sequence a large number of samples despite a substantial cost reduction in available technologies. Second, for target regions of tens to hundreds of kilobases or less for a single DNA sample, the smallest functional unit of a next-generation sequencer (for example, a single lane of an Illumina Genomic Analyzer II (GAII) or HiSeq2000 flow cell) generates a wasteful excess of coverage. Third, methods for individually indexing hundreds to thousands of samples are challenging to develop and limited in efficacy [5,6]. Fourth, generating sequence templates for target DNA regions in large numbers of samples is laborious and costly. Fifth, while pooling samples can reduce both labor and costs, it reduces sensitivity for the identification of rare variants using currently available next-generation sequencing strategies and bioinformatics tools [1,3].

We have optimized a flexible and efficient strategy that combines a PCR-based amplicon ligation method for template enrichment, sample pooling, and library indexing in conjunction with novel quality and filtering algorithms for identification of rare variants in large sample cohorts. For validation of this strategy, we present data from sequencing 12 indexed libraries of 40 samples each (total of 480 samples) using a single lane of a GAII Illumina Sequencer. We utilized an alternative base-calling algorithm, Srfim [7], and an automated filtering program, *SERVIC^4^E *(Sensitive Rare Variant Identification by Cross-pool Cluster, Continuity, and tailCurve Evaluation), designed for sensitive and reliable detection of rare variants in pooled samples. We validated this strategy using Illumina sequencing data from an additional independent cohort of 480 samples. Compared to publicly available software, this strategy achieved an excellent combination of sensitivity and specificity for rare variant detection in pooled samples through a substantial reduction of false positive and false negative variant calls that often confound next-generation sequencing. We anticipate that our pooling strategy and filtering algorithms can be easily adapted to other popular platforms of template enrichment, such as microarray capture and liquid hybridization [8,9].

## Results and discussion

### An optimized sample-pooling strategy

**Figure 1:**
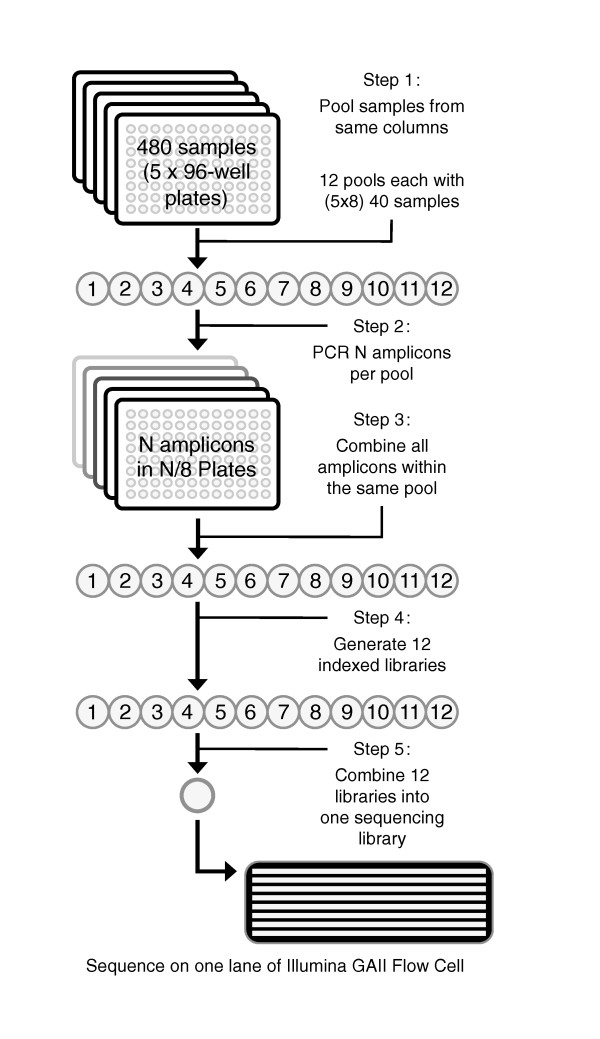
**Schematic diagram of the sequencing strategy**. Sample pools of 40 samples × 12 pools were generated from a cohort of 480 individuals for PCR amplification of individual exons. After blunt-ended ligation and random fragmentation, PCR amplicons from individual sample pools were used to generate indexed sequence libraries. The 12 indexed libraries were combined in equal molar amounts and sequenced in one lane of a flow cell using an Illumina GAII.

**Figure 2:**
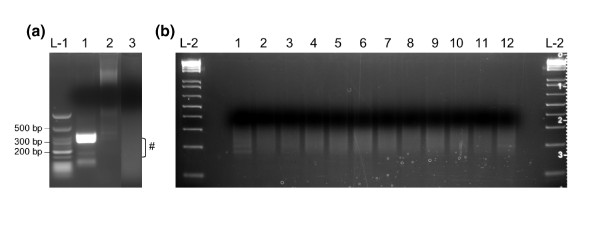
**Amplicon ligation, fragmentation and indexed Illumina libraries**. **(a) **Amplicon ligation and fragmentation: L-1, low molecular weight marker; lane 1, PCR amplicons before ligation; lane 2, PCR amplicons after ligation; lane 3, random fragmentation using Fragmentase (NEB). ^#^The bracket indicates fragments of desired length. **(b) **Indexed Illumina libraries: L-2, 1-kb ladder; lanes 1 to 12, size distribution of 12 indexed Illumina libraries.

We utilized a PCR-based amplicon-ligation method because PCR remains the most reliable method of template enrichment for selected regions in a complex genome. This approach ensures low cost and maximal flexibility in study design compared to other techniques [9-11]. Additionally, PCR of pooled samples alleviates known technical issues associated with PCR multiplexing [12]. We sequenced 24 exon-containing regions (250 to 300 bp) of a gene on chromosome 3, *GRIP2 *(encoding glutamate-receptor interacting protein 2; [GenBank: AB051506]) in 480 unrelated individuals (Figure 1). The total targeted region is 6.7 kb per sample. We pooled 40 DNA samples at equal concentration into 12 pools, which was done conveniently by combining samples from the same columns of five 96-well plates. We separately amplified each of the 24 regions for each pool, then normalized and combined resulting PCR products at equal molar ratio. The 12 pools of amplicons were individually blunt-end ligated and randomly fragmented for construction of sequencing libraries, each with a unique Illumina barcode [13]. These 12 indexed libraries were combined at equal molar concentrations and sequenced on one lane of a GAII (Illumina) using a 47-bp single-end module. We aimed for 30-fold coverage for each allele. Examples of amplicon ligation, distribution of fragmented products, and 12 indexed libraries are shown in Figure 2.

### Data analysis and variant calling

**Figure 3:**
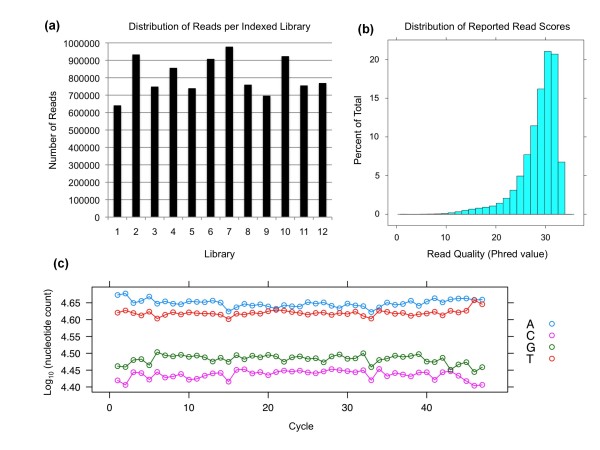
**Quality assessment of the Illumina sequence data**. **(a) **Number of reads with barcodes that passed Illumina filtering and aligned to the reference templates using Bowtie from individually indexed libraries (*n *= 12). Range, 641 k to 978 k reads; mean ± standard deviation, 809 k ± 107 k. **(b) **Percentage of total (unaligned) reads that fall into a mean Phred quality interval. Note > 80% of the reads have mean Phred quality scores ≥25. **(c) **Nucleotide content as a function of sequencing cycles (*n *= 47). Note that the nucleotide proportions closely match the expected proportions as determined from the templates.

**Figure 4:**
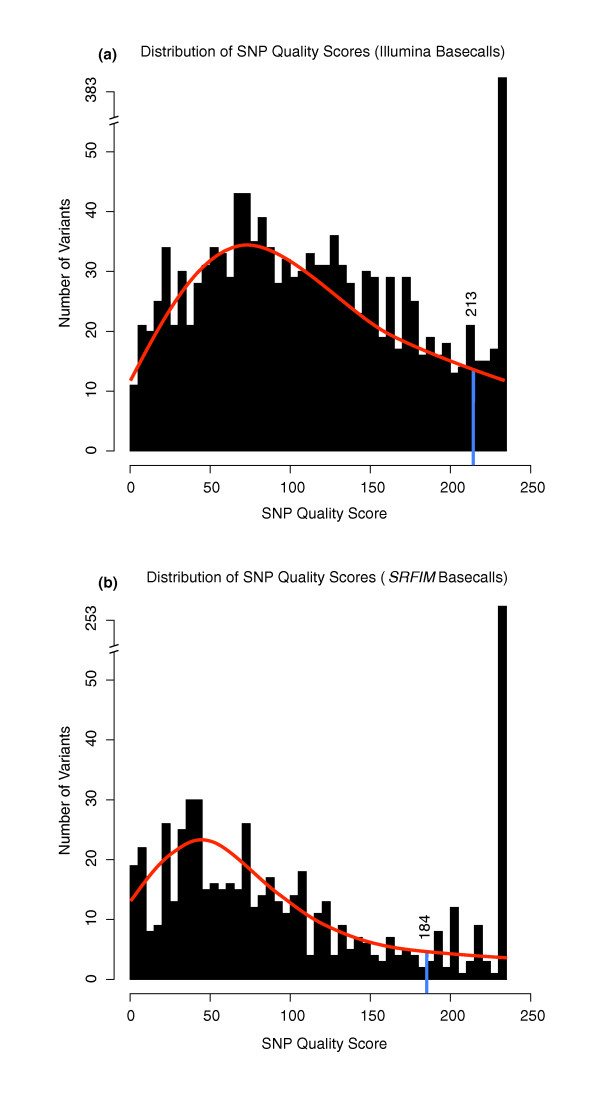
**Distribution of quality score from SAMtools Pileup**. Filtering was conducted at the 95th percentile of the consensus and SNP quality distributions reported by SAMtools; only the distribution of SNP quality values is depicted here. The blue bar is the 95th percentile score cutoff, discounting variants with max score. **(a) **SNP quality scores derived from Illumina base calls. **(b) **SNP quality scores derived from Srfim base calls.

Sequence reads were mapped by Bowtie using strict alignment parameters (-v 3: entire read must align with three or fewer mismatches) [14]. We chose strict alignment to focus on high quality reads. Variants were called using SAMtools (deprecated algorithms [pileup -A -N 80]; see Materials and methods) [15]. A total of 11.1 million reads that passed Illumina filtering and had identifiable barcodes were aligned to the human genome (hg19), generating approximately 520 megabases of data. The distribution of reads for each indexed library ranged from 641 k to 978 k and 80% of reads had a reported read score (Phred) greater than 25 (Figure 3a, b). The aggregate nucleotide content of all reads in the four channels across sequencing cycles was constant (Figure 3c), indicating a lack of global biases in the data. There was little variability in total coverage per amplicon pool, and sufficient coverage was achieved to make variant calling possible from all amplicon pools (Additional file 1). Our data indicated that 98% of exonic positions had an expected minimum coverage of 15× per allele (approximately 1,200× minimum coverage per position) and 94% had an expected minimum coverage of 30× (approximately 2,400× minimum coverage per position). Overall average expected allelic coverage was 68×. No exonic positions had zero coverage. To filter potential false positive variants from SAMtools, we included only high-quality variant calls by retaining variants with consensus quality (cq) and SNP quality (sq) scores in 95% of the score distributions (cq ≥ 196, sq ≥ 213; Figure 4a). This initially generated 388 variant calls across the 12 pools. A fraction of these variant calls (*n *= 39) were limited to single pools, indicating potential rare variants.

### Tailcurve analysis

**Figure 5:**
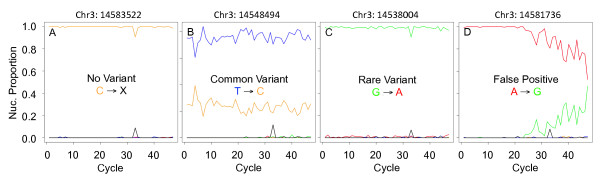
**Representative base reads and tailcurves for common and rare variants and error calls**. **(a) **Position with no variant. **(b) **Position with a common variant. **(c) **Position with a rare variant. **(d) **Position with a false positive call.

Initial validations by Sanger sequencing indicated that approximately 25% or more of these variant calls were false positives. Sequencing errors contribute to false positive calls and are particularly problematic for pooled samples where rare variant frequencies approach the error rate. To determine the effect of cycle-dependent errors on variant calls [7], we analyzed the proportions of each nucleotide called at each of the 47 sequencing cycles in each variant. We refer to this analysis as a tailcurve analysis due to the characteristic profile of these proportion curves in many false-positive variant calls (Figure 5; Additional file 2). This analysis indicated that many false positive calls arise from cycle-dependent errors during later sequencing cycles (Figure 5d). The default base-calling algorithm (BUSTARD) and the quality values it generates make existing variant detection software prone to false positive calls because of these technical biases. Examples of tailcurves reflecting base composition by cycle at specific genetic loci for wild type, common SNP, rare variant, and false positive calls are shown in Figure 5.

### Quality assessment and base calling using *SRFIM*

To overcome this problem, we utilized Srfim, a quality assessment and base-calling algorithm based on a statistical model of fluorescence intensity measurements that captures the technical effects leading to base-calling biases [7]. Srfim explicitly models cycle-dependent effects to create read-specific estimates that yield a probability of nucleotide identity for each position along the read. The algorithm identifies nucleotides with highest probability as the final base call, and uses these probabilities to define highly discriminatory quality metrics. Srfim increased the total number of mapped reads by 1% (to 11.2 million), reflecting improved base-calling and quality metrics, and reduced the number of variant calls by 20% (308 variants across 12 pools; 33 variant calls present in only a single pool).

### Cross-pool filtering using *SERVIC^4^E*

Further validation by Sanger sequencing indicated the persistence of a few false positive calls from this dataset. Analysis of these variant calls allowed us to define statistics that capture regularities in the base calls and quality values at false positive positions compared to true variant positions. We developed *SERVIC^4^E*, an automated filtering algorithm designed for high sensitivity and reliable detection of rare variants using these statistics.

**Figure 6:**
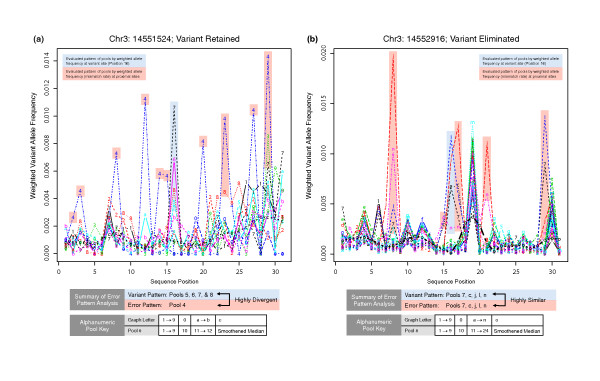
**Local pool patterns for error analysis**. X-axes denote position in a local sequence. Position 16 is the variant site being analyzed, positions 1 to 15 are immediately upstream and positions 17 to 31 are downstream. Y-axes denote the weighted allele frequency of the most prominent non-reference allele at each position (mismatch rate). Individual pools are denoted by a unique line pattern, color, and number/letter. Light shading indicates the pool pattern that is most recognizable by *SERVIC^4^E *for each position. **(a) **Local weighted allele frequencies for each pool at position 14,551,524 ± 15 in chromosome 3 from the first cohort. The evaluated pattern of pools at the variant position involves pools 5, 6, 7, and 8, while the evaluated pattern at proximal positions involves pool 4. The dissimilarity between patterns results in retention of chr3:14551524 as a variant site. **(b) **Local weighted allele frequencies for each pool at position 14,552,916 ± 15 in chromosome 3 from the second cohort. The evaluated pattern of pools at the variant position involves pools 7, 13 (c), 20 (j), 22 (l), and 24 (n), and the evaluated pattern at proximal positions involves the same pools. The similarity between patterns results in elimination of chr3:14552916 as a variant site.

Our filtering methods are based on four statistics derived from the coverage and qualities of variant calls at each position and pool: (1) continuity, defined as the number of cycles in which the variant nucleotide is called (ranges from 1 to 47); (2) weighted allele frequency, defined as the ratio of the sum of Phred quality scores of the variant base call to the sum of Phred quality scores of all base calls; (3) average quality, defined as the average quality of all base calls for a variant; and (4) tailcurve ratio, a metric that captures strand-specific tailcurve profiles that are characteristic of falsely called variants. *SERVIC^4^E *employs filters based on these four statistics to remove potential false-positive variant calls. Additionally, *SERVIC^4^E *searches for patterns of close-proximity variant calls, a hallmark of errors that have been observed across different sequenced libraries and sequencing chemistries (Figure 6), and uses these patterns to further filter out remaining false positive variants. In the next few paragraphs we provide rationales for our filtering statistics, and then define the various filters employed.

**Figure 7:**
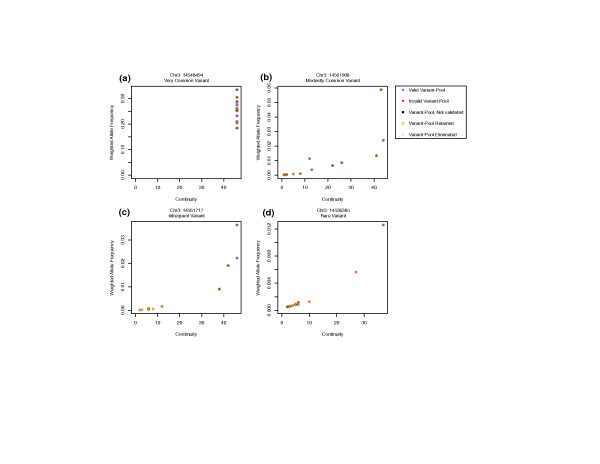
**Continuity versus weighted allele frequency curves for select variants**. **(a) **Very common variant present in all 12 pools. **(b) **Modestly common variant present in the majority of pools. **(c) **Infrequent variant present in a minority of pools. **(d) **Rare variant present in only one pool. Gold circles indicate variant pools retained by cluster analysis, while a gold 'x' indicates a variant pool that has been eliminated.

The motivation for using continuity and weighted allele frequency is based on the observation that a true variant is generally called evenly across all cycles, leading to a continuous representation of the variant nucleotide along the 47 cycles, and is captured by a high continuity score. However, continuity is coverage-dependent and should only be reliable when the variant nucleotide has sufficient sequencing quality. For this reason, continuity is assessed in the context of the variant's weighted allele frequency. Examples of continuity versus weighted allele frequency curves for common and rare variants are shown in Figure 7. Using these two statistics, *SERVIC^4^E *can use those pools lacking the variant allele (negative pools) as a baseline to isolate those pools that possess the variant allele (positive pools).

*SERVIC^4^E *uses a clustering analysis of continuity and weighted allele frequency to filter variant calls between pools. We use k-medioid clustering and decide the number of clusters using average silhouette width [16]. For common variants, negative pools tend to cluster and are filtered out while all other pools are retained as positives (Figure 7a, b). Rare variant pools, due to their lower allele frequency, will have a narrower range in continuity and weighted allele frequency. Negative pools will appear to cluster less, while positive pools cluster more. *SERVIC^4^E *will retain as positive only the cluster with highest continuity and weighted allele frequency (Figure 7c, d).

**Figure 8:**
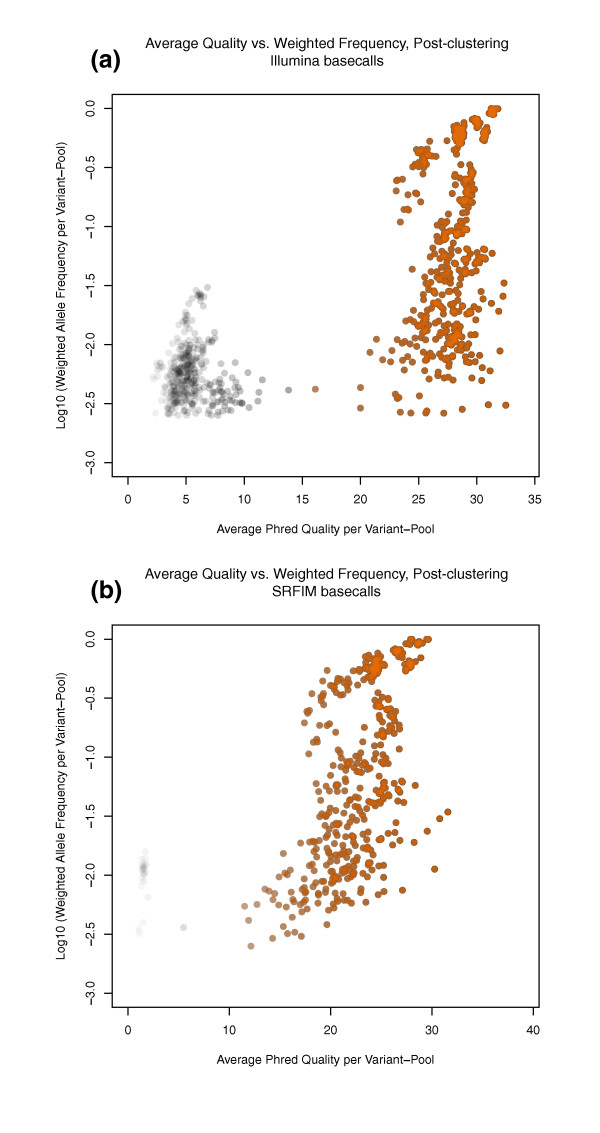
**Average quality versus weighted allele frequency for variant pools after filtering by clustering**. The X-axis is average Phred sequencing quality score and the Y-axis is weighted allele frequency (ratio of the sum of Phred quality scores for the variant allele at a position to the sum of all Phred quality scores at that position) in log_10 _scale. Characteristic distribution shapes make it possible to cluster and retain only high quality variants (orange points). **(a) **Illumina base calls. **(b) **Srfim base calls.

The second filter used by *SERVIC^4^E *is based on the average quality of the variant base calls at each position. One can expect that the average quality score is not static, and can differ substantially between different sequencing libraries and even different base-calling algorithms. As such, the average quality cutoff is best determined by the aggregate data for an individual project (Figure 8). Based on the distribution of average qualities analyzed, *SERVIC^4^E *again uses cluster analysis to separate and retain the highest quality variants from the rest of the data. Alternatively, if the automated clustering method is deemed unsatisfactory for a particular set of data, a more refined average quality cutoff score can be manually provided to *SERVIC^4^E*, which will override the default clustering method. For our datasets, we used automated clustering to retain variants with high average quality.

The third filtering step used by *SERVIC^4^E *captures persistent cycle-dependent errors in variant tailcurves that are not eliminated by Srfim. Cycle-specific nucleotide proportions (tailcurves) from calls in the first half of sequencing cycles are compared to the proportions from calls in the second half of sequencing cycles. The ratio of nucleotide proportions between both halves of cycles is calculated separately for plus and minus strands, thereby providing the tailcurve ratio added sensitivity to strand biases. By default, variant calls are filtered out if the tailcurve ratio differs more than ten-fold; we do not anticipate that this default will need adjustment with future sequencing applications, as it is already fairly generous, chiefly eliminating variant pools with clearly erroneous tailcurve ratios. This default was used for all our datasets.

The combination of filtering by average quality and tailcurve structure eliminates a large number of false variant calls. Additional file 3 demonstrates the effect of these filtering steps applied sequentially on two sets of base call data.

In addition to these filtering steps, *SERVIC^4^E *employs limited error modeling. The pattern of errors observed in many libraries may be dependent on the sequence context of the reads, the preparation of the library being sequenced, the sequencing chemistry used, or a combination of these three factors. We have observed that certain erroneous variant calls tend to aggregate in proximity. These clusters of errors can sometimes occur in the same positions across multiple pools. These observations appeared in two independent datasets in our studies. Importantly, many of the false positive calls that escaped our tailcurve and quality filtering fell within these clusters of errors. To overcome this problem, *SERVIC^4^E *conducts error filtering by analyzing mismatch rates in proximity to a variant position of interest and then determining the pattern of error across multiple pools. This pattern is defined as the most frequently occurring combination of pools with high mismatch rates at multiple positions within the isolated regions. The similarity between a variant call of interest and the local pattern or error across pools can then be used to eliminate that variant call (Figure 6). The consequences of these sequential filtering steps on variant output are outlined in Table 1 for both cohorts tested in this study.

**Table 1:** Effect of sequential filtering by *SERVIC^4^E *on variant output

	Dataset 1		Dataset 2
		
	Illumina	Srfim	Illumina
Number of variant-pools after filtering			
Prior to cluster analysis	1,656	1,056	7,272
After cluster analysis	929	905	5,123
After average quality filtering	437	342	422
After tailcurve filtering	341	340	412
After error-modeling	333	334	378

Reported values indicate the total number of variant positions (across all pools) that remain after each filtering step. Dataset 1: sequencing output of GRIP2 exons in a first cohort of 480 samples. Dataset 2: sequencing output of GRIP2 exons in a second independent cohort of 480 samples.

Finally, *SERVIC^4^E *provides a trim parameter that masks a defined length of sequence from the extremes of target regions from variant calling. This allows for *SERVIC^4^E *to ignore spurious variant calling that may occur in primer regions as a result of the concatenation of amplicons. By default, this parameter is set to 0; for our datasets, we used a trim value of 25, which is the approximate length of our primers.

### Reliable detection of rare variants in pooled samples

Using *SERVIC^4^E*, we identified 68 unique variants (total of 333 among 12 pools), of which 34 were exonic variants in our first dataset of 480 samples (Additional file 4). For validation, we performed Sanger sequencing for all exonic variants in individual samples in at least one pool. A total of 4,050 medium/high-quality Sanger traces were generated, targeting approximately 3,380 individual amplicons. Total coverage in the entire study by Sanger sequencing was approximately 930 kb (approximately 7.3% of total coverage obtained by high-throughput sequencing). Sanger sequencing confirmed 31 of the 34 variants. Fifteen rare exonic variants were identified as heterozygous in a single sample in the entire cohort.

### A comparison with available variant calling algorithms

We compared our variant calling method to publicly available algorithms, including SAMtools, SNPSeeker, CRISP, and Syzygy [1,3,15,17]. Because some variants are present and validated in multiple pools and each pool is considered as an independent discovery step, we determined the detection sensitivity and specificity on a variant pool basis. Results are shown in Table 2.

**Table 2:** Validation analysis of variant calling from first cohort samples

	Illumina					Srfim			
		
	SNPSeeker	SAMTools*	CRISP	Syzygy	*SERVIC^4^E*	SAMTools^#^	CRISP	Syzygy	*SERVIC^4^E*
Variant identification and validation									
True positive	56	83	79	85	88	80	78	80	84
True negative	72	41	44	49	62	60	50	51	61
False positive	2	32	29	24	11	13	23	22	12
False negative	34	7	11	5	2	10	12	10	6
Statistical analysis (%)									
Sensitivity	62.22	92.22	87.78	94.44	97.78	88.89	86.67	88.89	93.33
Specificity	97.26	56.16	60.27	67.12	84.93	82.19	68.49	69.86	83.56
PPV	96.55	72.17	73.15	77.98	88.89	86.02	77.23	78.43	87.50
NPV	67.62	85.42	80.00	90.74	96.88	85.71	80.65	83.61	91.04
FPR	2.74	43.84	39.73	32.88	15.07	17.81	31.51	30.14	16.44
FDR	3.45	27.83	26.85	22.02	11.11	13.98	22.77	21.57	12.50
Accuracy	77.91	76.07	75.46	82.21	92.02	85.89	78.53	80.37	88.96
MCC	61.78	52.79	50.54	65.05	84.22	71.41	56.50	60.37	**77.72**
Rare exonic variant detection and validation									
Detected total variants (*n *= 15)	7	15	13	14	15	14	12	10	15
Detection rate (%)	46.67	100	86.67	93.33	100	93.33	80.00	66.67	100

Descriptions of calculations used in statistical data analysis are provided in Materials and methods. FDR, false discovery rate; FPR, false positive rate; MCC, Matthews correlation coefficient; NPV, negative predictive value; PPV, positive predictive value. SNPSeeker: variant called uses the first 15 cycles (author recommended). *P*-value cutoff of 0.05 gave the best results. SAMTools* pileup -A -N 80: filtered for variants with consensus quality score ≥ 194 and SNP quality scores ≥ 213. CRISP: all 47 cycles used in alignment. Minimum base quality set to a default of 10. Syzygy: default parameters used. SAMTools^# ^pileup -A -N 80: filtered for variants with consensus quality score ≥ 161 and SNP quality scores ≥ 184. *SERVIC^4^E*: default parameters used.

To call variants with SAMtools [15], we used the deprecated Maq algorithms (SAMtools pileup -A -N 80), as the regular SAMtools algorithms failed to identify all but the most common variants. As a filtering cutoff we retained only the top 95th percentile of variants by consensus quality and SNP quality score (cq ≥ 196 and sq ≥ 213 for standard Illumina base calls, Figure 4a; cq ≥ 161 and sq ≥ 184 for Srfim base calls, Figure 4b).

SNPSeeker [1] uses large deviation theory to identify rare variants. It reduces the effect of sequencing errors by generating an error model based on internal negative controls. We used exons 6 and 7 as the negative controls in our analysis (total length = 523 bp) as both unfiltered SAMtools analysis and subsequent Sanger validation indicated a complete absence of variants in both exons across all 12 pools. Only Illumina base calls were used in this comparison because of a compatibility issue with the current version of Srfim. The authors of SNPSeeker recently developed a newer variant caller called SPLINTER [18], which requires both negative and positive control DNA to be added to the sequencing library. SPLINTER was not tested due to the lack of a positive control in our libraries.

CRISP [17] conducts variant calling using multiple criteria, including the distribution of reads and pool sizes. Most importantly, it analyzes variants across multiple pools, a strategy also employed by *SERVIC^4^E*. CRISP was run on both Illumina base calls and Srfim base calls using default parameters.

Syzygy [3] uses likelihood computation to determine the probability of a non-reference allele at each position for a given number of alleles in each pool, in this case 80 alleles. Additionally, Syzygy conducts error modeling by analyzing strand consistency (correlation of mismatches between the plus and minus strands), error rates for dinucleotide and trinucleotide sequences, coverage consistency, and cycle positions for mismatches in the read [19]. Syzygy was run on both Illumina and Srfim base calls, using the number of alleles in each pool (80) and known dbSNP positions as primary input parameters.

*SERVIC^4^E *was run using a trim value of 25 and a total allele number of 80. All other parameters were run at default. The focus of our library preparation and analysis strategy is to identify rare variants in large sample cohorts, which necessitates variant calling software with very high sensitivity. At the same time, specificity must remain high, primarily to ease the burden during validation of potential variants. In addition to calculating sensitivity and specificity, we calculated the Matthews correlation coefficient (MCC; see Materials and methods) for each method (Table 2) in order to provide a more balanced comparison between the nine methods.

For validation of our dataset, we focused primarily on changes in the exonic regions of our amplicons. Any intronic changes that were collaterally sequenced successfully were also included in our final analysis (Table 2). Sixty-one exonic positions were called as having a variant allele in at least one pool by one or more of the nine combinations of algorithms tested. We generated Sanger validation data in at least one pool for 49 of the 61 positions identified. Genotypes for validated samples are indicated in Additional file 5.

SNPSeeker (with Illumina base calls) performed with the highest specificity (97.3%), but with the worst sensitivity (62.2%), identifying less than half of the 15 valid rare exonic variants (Table 2). This is likely due to an inability of this algorithm to discriminate variants with very low allele frequencies in a pool; 84% of SNPSeeker's true positive calls have an allele frequency ≥ 1/40, while only 13% of the false negative calls have a frequency ≥ 1/40 (Additional files 4 and 6). SNPSeeker's MCC score was low (61.8%), due in large part to its very low false positive rate.

SAMtools alone with Illumina base calls achieved a 92.2% sensitivity, identifying all 15 rare exonic variants; however, these results were adulterated with the highest number of false positives, resulting in the worst specificity (56.2%) and MCC score (52.8%) among the nine methods (Table 2). Incorporation of Srfim base calls cut the number of false positives by 60% (from 32 to 13) without a sizeable reduction in the number of true positive calls (from 83 to 80). Fourteen of the fifteen valid rare exonic variants were successfully identified, which while not perfect, is an acceptably high sensitivity (Table 2). Srfim made noticeable improvements to individual base quality assessment as reflected in a substantial reduction in low quality variant calls (Figure 4) by reducing the contribution of low quality base calls to the average quality distribution (Figure 8b) and by reducing the tailcurve effect that leads to many false positives (Additional file 3a, b). Most low quality variant calls eliminated when transitioning to Srfim were not valid; nonetheless, three low quality valid variant calls were similarly affected by Srfim, and their loss resulted in a slight reduction in the true positive rate.

CRISP using Illumina base calls achieved a sensitivity slightly lower than SAMtools (87.8% versus 92.2%). Additionally, CRISP identified only 13 of the 15 valid rare exonic variants. Though this is lower than SAMtools, it is a large improvement over SNPSeeker; for the purposes set forth in our protocol, the > 75% sensitivity for extremely rare variants achieved by CRISP (using either base-calling method) is acceptable (Table 2).

Syzygy achieved the second highest sensitivity (94.4%) using Illumina base calls, but specificity remained low (67.1%). Fourteen of the fifteen rare exonic variants were successfully identified. CRISP and Syzygy achieved relatively average MCC values (50.5% and 65.0%, respectively), reflecting better performance than SAMtools with Illumina base calls.

*SERVIC^4^E *using Illumina base calls achieved the highest sensitivity (97.8%) and identified all 15 valid rare exonic variants. Both sensitivity and specificity were improved over SAMtools, CRISP, and Syzygy (Table 2), reflected in the highest MCC score of all the tested methods (84.2%). Taken together, the combination of *SERVIC^4^E *with either base-calling algorithm provides the highest combination of sensitivity and specificity in the dataset from pooled samples.

As previously mentioned, Srfim greatly improved variant calling in SAMtools, as is reflected in the 19% increase in SAMtools' MCC value (from 52.8% to 71.4%). CRISP, Syzygy, and *SERVIC^4^E *benefited little from using Srfim base calls: the MCC value for CRISP improved by only 6% (from 50.5% to 56.5%), Syzygy diminished by 4.6% (from 65.0% to 60.4%), and *SERVIC^4^E *diminished by 6.5% (from 84.2% to 77.7%). Importantly, use of Srfim base calls with Syzygy diminished its capacity to detect rare variants by a third. These three programs are innately designed to distinguish low frequency variants from errors using many different approaches. As such, it can be inferred from our results that any initial adjustments to raw base calls and quality scores by the current version of Srfim will do little to improve that innate capacity. In contrast, SAMtools, which is not specifically built for rare variant detection and would therefore have more difficulty distinguishing such variants from errors, benefits greatly from the corrective pre-processing provided by Srfim.

In addition to performance metrics like sensitivity and specificity, we analyzed annotated SNP rates, transition-transversion rates, and synonymous-non-synonymous rates of the nine algorithms on a variant-pool basis (Additional file 7).

The variant pools with the greatest discrepancies between the various detection methods tended to have an estimated allele frequency within the pool that is less than the minimum that should be expected (1/80; Additional files 4, 6, and 8). Such deviations are inevitable, even with normalization steps, given the number of samples being pooled. This underscores the importance of having careful, extensive normalization of samples to minimize these deviations as much as possible, and the importance of using variant detection methods that are not heavily reliant on allele frequency as a filtering parameter or are otherwise confounded by extremely low allele frequencies.

### Validation using data from an independent cohort of samples

To further assess the strength of our method and analysis software, we sequenced the same 24 *GRIP2 *exons in a second cohort of 480 unrelated individuals. The same protocol for the first cohort was followed, with minor differences. Firstly, we pooled 20 DNA samples at equal concentration into 24 pools. The first 12 pools were sequenced in one lane of a GAII and the last 12 pools were sequenced in a separate lane (Additional file 9). Additionally, the libraries were sequenced using the 100-bp paired-end module, and sequencing was conducted using a newer version of Illumina's sequencing chemistry. These 24 libraries occupied approximately 5% of the total sequencing capacity of the two lanes. The remaining capacity was occupied by unrelated libraries that lacked reads originating from the *GRIP2 *locus

To map reads from this dataset, we initially used Bowtie's strict alignment parameters (-v 3), as we had done with our first dataset, but this resulted in a substantial loss of coverage in the perimeters of target regions. This is likely due to reads that cross the junctions between our randomly concatenated amplicons; such reads, which have sequence from two distant amplicons, appear to have extensive mismatching that would result in their removal. This effect became pronounced when using long read lengths (100 bp), but was not noticeable when using the shorter reads in our first dataset (Additional file 10). This effect should not be an issue when using hybridization enrichment, where ligation of fragments is not needed.

In order to improve our coverage, we used Bowtie's default parameter, which aligns the first 28 bases of each read, allowing no more than two mismatches. To focus on *GRIP2 *alignments, we provided a fasta reference of 60 kb covering the *GRIP2 *locus. A total of 6.4 million reads (5.6% of all reads) aligned to our reference template of the *GRIP2 *locus. The depth of coverage for each amplicon pool is shown in Additional file 11. For exonic positions, the average allelic coverage was 60.8×, and the minimum coverage was 10×; 99.9% of exonic positions were covered at least 15× per allele, and 98.5% were covered at least 30× per allele.

We did not apply Srfim base calls to our variant calling as Srfim has not yet been fully adapted to the newer sequencing chemistry used with this cohort. For variant calling, we tested Syzygy and *SERVIC^4^E*, the two most sensitive software identified in our first dataset when using only the standard Illumina base calls (Table 2). Syzygy was provided with a template-adjusted dbSNP file and a total allele number of 40 as input parameters. All other parameters were run at default. Syzygy made a total of 474 variant calls across 24 pools (74 unique variant calls). Of the 74 unique calls made, 36 were exonic changes. *SERVIC^4^E *was run using a trim value of 25 and a total allele number of 40. All other parameters were run at default. *SERVIC^4^E *made a total of 378 variant calls across 24 pools (68 unique variant calls). Of the 68 unique calls made, 33 were exonic changes. Between Syzygy and *SERVIC^4^E*, a total of 42 unique exonic sequence variant calls were made (Additional files 12 and 13).

For validation of these results, we again targeted variants within exons for Sanger sequencing. Sanger data were successfully obtained from individual samples in at least one pool for 41 of the 42 exonic variants. Genotypes for validated samples are indicated in Additional file 14. Results are summarized in Table 3 and include any intronic variant pools that were collaterally Sanger sequenced successfully. Of the 41 exonic variants checked, 29 were valid. Sixteen were identified as occurring only once in the entire cohort of 480 individuals. Syzygy achieved a high sensitivity of 85.5% but a fairly low specificity of 59.4%. Of the 16 valid rare exonic variants, 13 (81.25%) were identified. The MCC score was low (45.9%), primarily as a result of the low specificity (Table 3). *SERVIC^4^E *achieved a higher sensitivity of 96.4% and a higher specificity of 93.8%. All 16 valid rare exonic variants were identified and a high MCC score (89.9%) was obtained. The combined analysis of the first and second cohorts identified 47 valid coding variants, of which 30 were present only once in each cohort.

**Table 3:** Validation analysis of variant calling from second cohort samples

	Illumina	
	
	Syzygy	*SERVIC^4^E*
Variant identification and validation		
True positive	47	53
True negative	38	60
False positive	26	4
False negative	8	2
Statistical analysis (%)		
Sensitivity	85.45	96.36
Specificity	59.38	93.75
PPV	64.38	92.98
NPV	82.61	96.77
FPR	40.63	6.25
FDR	35.62	7.02
Accuracy	71.43	94.96
MCC	45.90	89.93
Rare exonic variant detection and validation		
Detected total variants (*n *= 16)	13	16
Positive detection rate (%)	81.25	100

Descriptions of calculations used in statistical data analysis are provided in Materials and methods. FDR, false discovery rate; FPR, false positive rate; MCC, Matthews correlation coefficient; NPV, negative predictive value; PPV, positive predictive value For both algorithms, an allele count of 40 was used. Syzygy: default parameters used. *SERVIC^4^E*: trim value of 25 used. Default used for all other parameters.

## Conclusions

We have developed a strategy for targeted deep sequencing in large sample cohorts to reliably detect rare sequence variants. This strategy is highly flexible in study design and well suited to focused resequencing of candidate genes and genomic regions from tens to hundreds of kilobases. It is cost-effective due to substantial cost reductions provided by sample pooling prior to target enrichment and by the efficient utilization of next-generation sequencing capacity using indexed libraries. Though we utilized a PCR method for target enrichment in this study, other popular enrichment methods, such as microarray capture and liquid hybridization [8-10], can be easily adapted for this strategy.

Careful normalization is needed during sample pooling, PCR amplification, and library indexing, as variations at these steps will influence detection sensitivity and specificity. While genotyping positive pools will be needed for validation of individual variants, only a limited number of pools require sequence confirmation as this strategy is intended for discovery of rare variants.

*SERVIC^4^E *is highly sensitive to the identification or rare variants with minimal contamination by false positives. It consistently outperformed several publicly available analysis algorithms, generating an excellent combination of sensitivity and specificity across base-calling methods, sample pool sizes, and Illumina sequencing chemistries in this study. As sequencing chemistry continues to improve, we anticipate that our combined sample pooling, library indexing, and variant calling strategy should be even more robust in identifying rare variants with allele frequencies of 0.1 to 5%, which are within the range of the majority of rare deleterious variants in human diseases.

## Materials and methods

### Sample pooling and PCR amplification

De-identified genomic DNA samples from unrelated patients with intellectual disability and autism, and normal controls were obtained from Autism Genetics Research Exchange (AGRE), Greenwood Genomic Center, SC, and other DNA repositories [20]. An informed consent was obtained from each enrolled family at the respective institutions. The Institutional Review Board at the Johns Hopkins Medical Institutions approved this study.

DNA concentration from each cohort of 480 samples in 5 × 96-well plates was measured using a Quant-iT™ PicoGreen^® ^dsDNA Kit (Invitrogen, Carlsbad, CA, USA) in a Gemini XS Microplate Spectrofluorometer. These samples were normalized and mixed at equal molar ratio into 12 pools of 40 samples each (first cohort) or 24 pools of 20 samples each (second cohort). For convenience, first cohort samples from the same column of each 5 × 96-well plate were pooled into a single well (Figure 1). The same principle was applied to the second cohort, with the first two and a half plates combined into the first 12 pools, and the last two and a half plates combined into the last 12 pools (Additional file 9). PCR primers for individual amplicons were designed using the Primer3 program. PCR reaction conditions were optimized to result in a single band of the expected size. Phusion Hot Start High-Fidelity DNA Polymerase (Finnzymes, Thermo Fisher Scientific, Waltham, MA, USA) and limited amplification cycles (*n *= 25) were used to minimize random errors introduced during PCR amplification. PCR reactions were carried out in a 20- μl system containing 50 ng of DNA, 200 μM of dNTP, 1× reaction buffer, 0.2 μM of primers, and 0.5 units of Phusion Hot Start High-Fidelity Polymerase in a thermocycler with an initial denaturation at 98°C for 30 seconds followed by 25 cycles of 98°C for 10 seconds, 58 to 66°C for 10 seconds, and 72°C for 30 seconds. The annealing temperature was optimized for individual primer pairs. Successful PCR amplification for individual samples was then verified by agarose gel electrophoresis. The concentration for individual PCR products was measured using the Quant-iT™ PicoGreen^® ^dsDNA Kit (Invitrogen) on Gemini XS Microplate Spectrofluorometer, and converted to molarity. PCR amplicons intended for the same indexed library were combined at equal molar ratio, purified using QIAGEN (Hilden, Germany) QIAquick PCR Purification Kit, and concentrated using Microcon YM-30 columns (Millipore, Billerica, MA, USA).

### Amplicon ligation and fragmentation

The pooled amplicons were ligated using a Quick Blunting and Quick Ligation Kit (NEB, Ipswich, MA, USA) following the manufacturer's instructions. For blunting, a 25- μl reaction system was set up as follows: 1× blunting buffer, 2 to 5 μg of pooled PCR amplicons, 2.5 μl of 1 mM dNTP mix, and 1 μl of enzyme mix including T4 DNA polymerase (NEB #M0203) with 3' → 5' exonuclease activity and 5' → 3' polymerase activity and T4 polynucleotide kinase (NEB #M0201) for phosphorylation of the 5' ends of blunt-ended DNA. The reaction was incubated at 25°C for 30 minutes and then the enzymes were inactivated at 70°C for 10 minutes. The blunting reaction products were purified using a MinElute PCR purification column (QIAGEN) and then concentrated using a Microcon YM-30 column (Millipore) to 5 μl volume in distilled water. For ligation, 5 μl of 2× Quick-ligation buffer was mixed with 5 μl of purified DNA. Quick T4 DNA ligase (1 μl; NEB) was added to the reaction mixture, which was incubated at 25°C for 5 minutes and then chilled on ice. The reaction product (0.5 μl) was checked for successful ligation using 1.5% agarose gel electrophoresis. The ligation products were then purified using a MinElute PCR purification column (QIAGEN). Random fragmentation of the ligated amplicons was achieved using either one of the two methods: (1) nebulization in 750 μl of nebulization buffer at 45 psi for 4 minutes on ice following a standard protocol (Agilent); or (2) using a NEBNext dsDNA Fragmentase Kit following the manufacturer's instructions (NEB). One-twentieth of the product was analyzed for successful fragmentation to a desired range using 2% agarose gel electrophoresis.

### Library construction and Illumina sequencing

The Multiplexing Sample Preparation Oligonucleotide Kit (Illumina PE-400-1001) was used to generate 1 × 12 (first cohort) and 2 × 12 (second cohort) individually indexed libraries following the manufacturer's instructions. The indexed libraries were quantified individually and pooled at equal molar quantity. The concentration of the final pooled library was determined using a Bioanalyzer (Agilent). All 12 pooled libraries from the first cohort were run in one lane of a flow cell on an Illumina Genomic Analyzer II (GAII). The first 12 pooled libraries from the second cohort were run in one lane of a GAII, while the last 12 pooled libraries were run in another lane in the same flow cell. Illumina sequencing was done at the UCLA DNA Sequence Core and Genetic Resource Core Facility at the Johns Hopkins University.

### Sequence data analysis

Raw intensity files and fastq-formatted reads were provided for both cohort datasets. Output had been calibrated with control lane PhiX DNA to calculate matrix and phasing for base calling. A custom script was used on first cohort sequence data to identify the 12 Illumina barcodes from the minimum edit distance to the barcode and assign a read to that pool if the distance index was unique (demultiplexing). Second cohort sequence data were provided to us already demultiplexed. Read mapping was done independently on each pool using BOWTIE (options: -v 3 for first cohort, default for second cohort). As reference templates, hg19 was used for the first cohort and a 60-kb fragment of the *GRIP2 *regions was used for the second cohort (*GRIP2 *region- chr3:14527000-14587000).

Variant calling using SAMtools was done independently on each pool using SAMtools' deprecated algorithms (options: pileup -vc -A -N 80). Variants identified were first filtered by eliminating non-*GRIP2 *variants, and then filtered by consensus quality and SNP quality scores (cq ≥ 196 and sq ≥ 213 for Illumina base calls; cq ≥ 161 and sq ≥ 184 for Srfim base calls). Deprecated (Maq) algorithms were used, as the current SAMtools variant-calling algorithms failed to call all but the most common SNPs. Quality cutoff is based on the 95th percentile of scores in the quality distributions observed amongst all reported SAMtools variants in the *GRIP2 *alignment region, after excluding variants with the maximal quality score of 235). Reads were base-called using Srfim using default filtering and quality parameters.

*SERVIC^4^E *was given the location of sorted alignment (BAM) files. Though alignment files are maintained separately for each pool, the locations of each file are given all together. A trim value was set at 25. This trims 25 bases away from the ends of aligned amplicons, so that variant calling is focused away from primer regions. Use of shorter primers during library preparation allows for a smaller trim value. Hybridization enrichment will always result in a trim value of zero, regardless of what trim value is actually set. The total number of alleles in each pool was also provided as input (80 alleles for the first cohort; 40 alleles for the second cohort). *SERVIC^4^E *(release 1) does not call insertions or deletions.

SNPSeeker was run on first cohort data using author recommended parameters. Reads (Illumina base calls) were converted to SCARF format. Srfim base calls could not be used due to an unknown formatting issue after SCARF conversion. Alignment was conducted against *GRIP2 *template sequences. Exons 6 and 7 reference sequences were merged so that their alignments could be used as a negative control to develop an error model. All 47 cycles were used in the alignment, allowing for up to three mismatches. Alignments were tagged and concatenated, and an error model generated using all 47 cycles, allowing for up to three mismatches, and using no pseudocounts. The original independent alignment files (pre-concatenation) were used for variant detection. As per recommendation by the authors, the first third of cycles was used for variant detection (15 cycles). A *P*-value cutoff of 0.05 was used. Lower cutoffs generated worse results when checked against our validation database.

CRISP was run using default parameters. A CRISP-specific pileup file was generated using the author-provided sam_to_pileup.py script and not generated using the pileup function in SAMtools. A separate pileup was generated for each pool for both alignments from Illumina base calls and alignment from Srfim base calls. A BED file was provided to focus pileup at *GRIP2 *loci. CRISP analysis for variant detection was conducted using all 47 cycles and a minimum base quality of 10 (default). All other parameters were also kept at default.

Syzygy [3,19] was run on both cohorts using 80 and 40 as the total number of alleles, respectively. A dbSNP file was provided for known chromosome 3 variants. A TGF file was provided to focus variant calling at *GRIP2 *target regions. Hg19 was used as the reference sequence for the first cohort, while the same abridged *GRIP2 *sequence that was used by *SERVIC^4^E *was also used by Syzygy for the second cohort. All other parameters were run at default.

Reads used for analysis, both Illumina and Srfim base calls, are available through the public data repository at the NCBI (accession number SRP007694). Srfim is available as an R package, while *SERVIC^4^E *is available as a set of R scripts. Both are available for download online [21].

### Validation by Sanger sequencing

Sanger sequencing of positive pools for variant validation was conducted using the BigDye Terminator v3.1 Cycle Sequencing Kit on an ABI3100 automatic DNA analyzer (Applied Biosystems, Foster City, CA, USA) following the manufacturer's instructions.

Sanger sequencing was done on each sample within a pool separately (40 traces per pool with the first cohort, 20 traces per pool for the second cohort). Only traces with low quality or ambiguous calls were sequenced bidirectionally. In the event that a positive sample was verified at least once in the pool, further sequencing of that pool was halted. Sequencing primers were the same primers used in target enrichment to build the libraries for next-generation sequencing.

Standard sequence alignment software (CodonCode, MacVector) followed by manual investigations of the chromatograms was used to identify any variants that might have been missed by all nine combinations of programs.

### Calculations

#### Matthews correlation coefficient

The MCC is intended as a measure of true positives (TPs), true negatives (TNs), false positives (FPs), and false negatives (FNs), without being influenced by potential extreme sizes by one or more of the groups. An MCC = 1 indicates perfect correlation between predicted results (variants identified by next-generation sequencing and various combinations of base-calling and variant-calling algorithms) and the observed results (validation by Sanger sequencing). An MCC = 0 indicates that the algorithm is no better than random. An MCC = -1 indicates an inverse correlation. MCC = (TP × TN-FP × FN)/SQRT [(TP + FP) × (TP + FN) × (TN + FP) × (TN + FN)]. Sensitivity (true positive rate, recall): TP/(TP + FN). Specificity (true negative rate): TN/(FP + TN). Positive predictive value (precision): TP/(TP + FP). Negative predictive value: TN/(TN + FN). Accuracy: (TP + TN)/(TP + TN + FP + FN). False positive rate (fall-out): 1-True negative rate. False discovery rate: FP/(FP + TP).

## Abbreviations

bp: base pair; cq: consensus quality score generated by SAMtools pileup; GAII: Genome Analyzer II (Illumina Sequencing Machine); *GRIP2*: glutamate-receptor interacting protein 2; MCC: Matthews correlation coefficient; PCR: polymerase chain reaction; *SERVIC^4^E*: Sensitive Rare Variant Identification by Cross-pool Cluster: Continuity: and tailCurve Evaluation; SNP: single nucleotide polymorphism; sq: SNP quality score generated by SAMtools pileup.

## Authors' contributions

AA and TSN generated Illumina libraries and conducted sequence validation. TSN, HCB, and MAT performed sequence data analysis. RI, SW, and TW conceived of the study, participated in its design, and supervised its execution. TSN, AA, HCB and TW wrote the manuscript. All authors read and approved the final manuscript.

## Supplementary Material

Additional file 1 **Depth of coverage for each amplicon pool derived from first cohort sequencing data**. Blue line depicts absolute coverage for plus-strand aligned reads. Green line depicts coverage of minus-strand aligned reads. Scales of X- and Y-axes are identical for all graphs depicted for each exon. Light red line indicates presumptive mismatch rate determined from plus-strand aligned reads. Light orange line indicates presumptive mismatch rate determined from minus-strand aligned reads. Ratio of mismatch rate between plus and minus strands is later incorporate into the tailcurve factor used in filtering by *SERVIC^4^E*. Click here for file

Additional file 2 **Description of tailcurve (nucleotide proportion at individual cycles along the sequence read)**. With perfect random fragmentation, a given position and its associated base calls (consensus and variant) should be represented at multiple sequencing cycles. With high coverage, a particular base call will be present for that position at all or most cycles. Example: for a sequencing module of 25 cycles with several hundred (24 shown) overlapping reads covering the highlighted position, all the cycles are represented by 'G', with variant reads producing the 'T' at a handful of cycles (potential variant). Click here for file

Additional file 3 **Diagrammatic output of first three filtering steps using *SERVIC^4^E *on first cohort data**. Left-hand panel uses Illumina base calls. Right-hand panel uses Srfim base calls. Individual filtering steps progress while moving down each panel. Colored dots incorporate validation data for visualization purposes; blue dots are valid variant pools and red dots are invalid variant pools. Within each panel, the graphs on the left are Average quality versus Weighted allele frequency distributions. X-axis is average Phred quality for each variant-pool. Y-axis is log_10 _of weighted allele frequency. Histograms on the right depict the frequency of evaluated tailcurve ratios across bins of length = 2. Click here for file

Additional file 4 **Variant call results from first cohort analysis**. All positions are given in reference to chromosome 3 of hg19. For each program, a '+' value indicates that a variant call was made by that program for that variant position and pool. Column 'P' indicates the position is in exonic sequence (not intronic). Column 'Valid' indicates validation results for each variant-pool tested; '+' indicates a valid call and '-'indicates an invalid call. Column 'Dist' indicates the position of the variant call in each amplicon. Click here for file

Additional file 5 **Genotyping results for individual first cohort samples**. For all samples validated by Sanger sequencing, homozygous wild types are indicated by '-', heterozygotes are indicated by '+', and homozygous mutants are indicated by '++'. Click here for file

Additional file 6 **Variant call output of *SERVIC^4^E *on the first cohort using Illumina base calls**. Click here for file

Additional file 7 **Comparisons of annotated SNPs, transition-transversion ratios, and synonymous-non-synonymous ratios**. Calculated metrics for annotation rates, transition-transversion rates, and synonymous-non-synonymous rates for first cohort data only. Click here for file

Additional file 8 **Variant call output of *SERVIC^4^E *on the first cohort using Srfim base calls**. Click here for file

Additional file 9 **Pooling strategy for second cohort samples**. Example: Normalized DNA samples from column 12 of plates 1 and 2 as well as samples from plate 3, column 12, rows A, B, C, and D are pooled together to form pool 12. Normalized DNA samples from column 1 of plates 4 and 5 as well as samples from plate 3, column 1, rows E, F, G, and H are pooled together to form pool 13. Click here for file

Additional file 10 **Effect of strict alignment on coverage from concatenated amplicons**. Panel 1 indicates targets for amplification (primers denoted by black half-arrows). Color-coding for each unique target region is retained in all panels. Panel 2 depicts ligation (concatenation) of amplicons. Only two amplicons are depicted; in practice many amplicons ligate together in a row. Darker shaded regions are from primer sequence. Panel 3 depicts random fragmentation to generate 150- to 200-bp segments for sequencing. Panel 4 depicts subsequent strict alignment of short (left) and long (right) reads to genomic reference sequence. Click here for file

Additional file 11 **Depth of coverage for each amplicon pool derived from second cohort sequencing data**. Blue line depicts absolute coverage for plus-strand aligned reads. Green line depicts coverage of minus-strand aligned reads. Scales of X- and Y-axes are identical for all graphs depicted for each exon. Light red line indicates presumptive mismatch rate determined from plus-strand aligned reads. Light orange line indicates presumptive mismatch rate determined from minus-strand aligned reads. Ratio of mismatch rate between plus and minus strands is later incorporated into the tailcurve factor used in filtering by *SERVIC^4^E*. Click here for file

Additional file 12 **Variant call results from second cohort analysis**. All positions are given in reference to chromosome 3 of hg19. For each program, a '+' value indicates that a variant call was made by that program for that variant position and pool. Column 'P' indicates the position is in exonic sequence (not intronic). Column 'Valid' indicates validation results for each variant-pool tested; '+' indicates a valid call and '-' indicates an invalid call. Column 'Dist' indicates the position of the variant call in each amplicon. Click here for file

Additional file 13 **Variant call output of *SERVIC^4^E *on the second cohort using Illumina base calls**. Click here for file

Additional file 14 **Genotyping results for individual second cohort samples**. For all samples validated by Sanger sequencing, homozygous wild types are indicated by '-', heterozygotes are indicated by '+', and homozygous mutants are indicated by '++'. Click here for file
