# Supplementary material for: Effective detection of rare variants in pooled DNA samples using Cross-pool tailcurve analysis
Source: Genome Biol. 2011 Sep 28;12(9):R93. doi: 10.1186/gb-2011-12-9-r93 (PMC3308056; doi:10.1186/gb-2011-12-9-r93)
Supplement: Additional file 1 — Depth of coverage for each amplicon pool derived from first cohort sequencing data. Blue line depicts absolute coverage for plus-strand aligned reads. Green line depicts coverage of minus-strand aligned reads. Scales of X- and Y-axes are identical for all graphs depicted for each exon. Light red line indicates presumptive mismatch rate determined from plus-strand aligned reads. Light orange line indicates presumptive mismatch rate determined from minus-strand aligned reads. Ratio of mismatch rate between plus and minus strands is later incorporate into the tailcurve factor used in filtering by SERVIC4E. [file gb-2011-12-9-r93-S1.PDF]

Exon 25

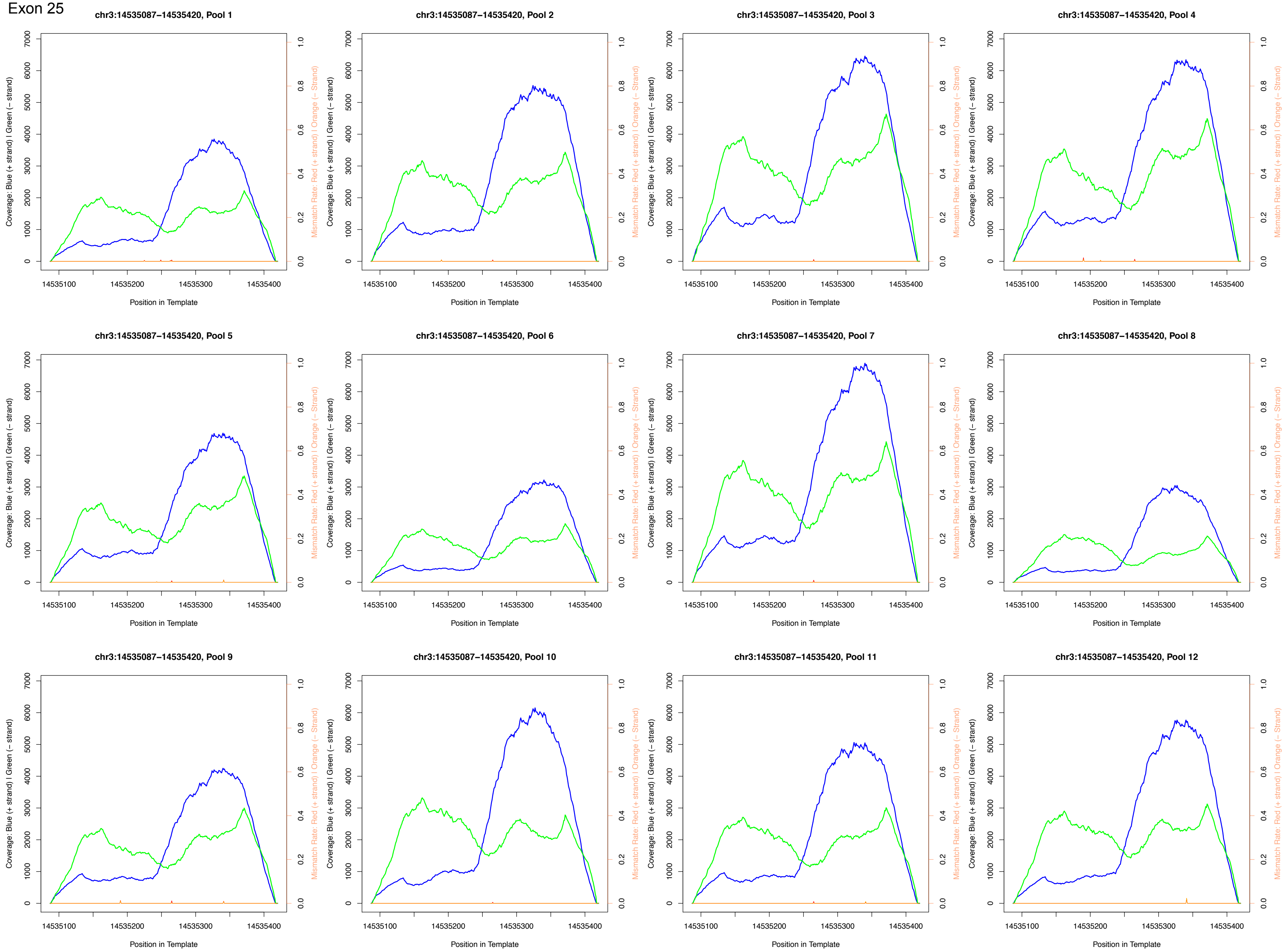
chr3:14535087–14535420, Pool 1
chr3:14535087–14535420, Pool 2
chr3:14535087–14535420, Pool 3
chr3:14535087–14535420, Pool 4
chr3:14535087–14535420, Pool 5
chr3:14535087–14535420, Pool 6
chr3:14535087–14535420, Pool 7
chr3:14535087–14535420, Pool 8
chr3:14535087–14535420, Pool 9
chr3:14535087–14535420, Pool 10
chr3:14535087–14535420, Pool 11
chr3:14535087–14535420, Pool 12
Coverage: Blue (+ strand) | Green (− strand)
Mismatch Rate: Red (+ strand) | Orange (− Strand)
Position in Template
0
1000
2000
3000
4000
5000
6000
7000
0.0
0.2
0.4
0.6
0.8
1.0
14535100
14535200
14535300
14535400

Exon 24

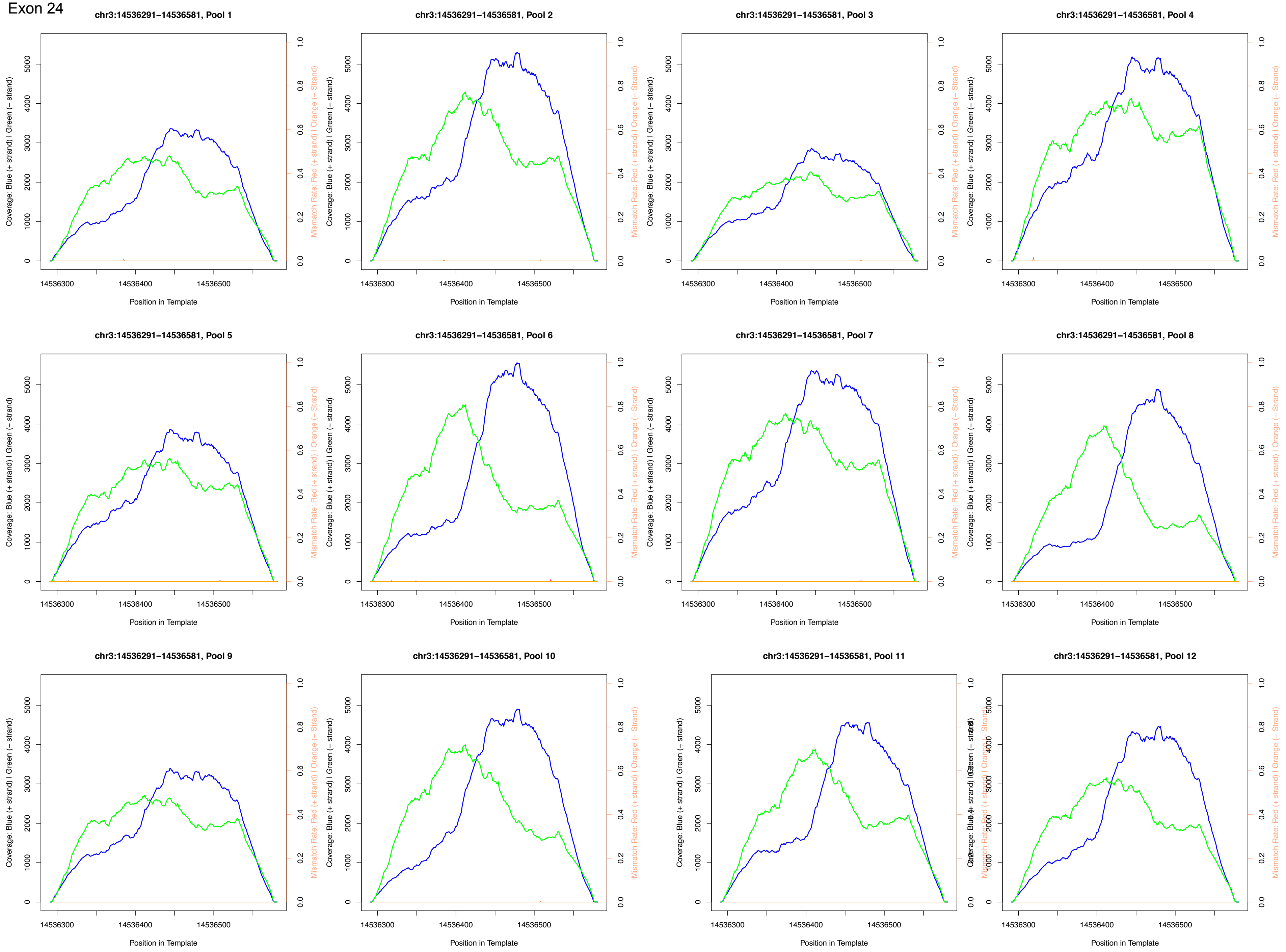

chr3:14536291–14536581, Pool 1
chr3:14536291–14536581, Pool 2
chr3:14536291–14536581, Pool 3
chr3:14536291–14536581, Pool 4
chr3:14536291–14536581, Pool 5
chr3:14536291–14536581, Pool 6
chr3:14536291–14536581, Pool 7
chr3:14536291–14536581, Pool 8
chr3:14536291–14536581, Pool 9
chr3:14536291–14536581, Pool 10
chr3:14536291–14536581, Pool 11
chr3:14536291–14536581, Pool 12
Coverage: Blue (+ strand) | Green (− strand)
Mismatch Rate: Red (+ strand) | Orange (− Strand)
Position in Template
14536300
14536400
14536500
0
1000
2000
3000
4000
5000
0.0
0.2
0.4
0.6
0.8
1.0

Exon 23

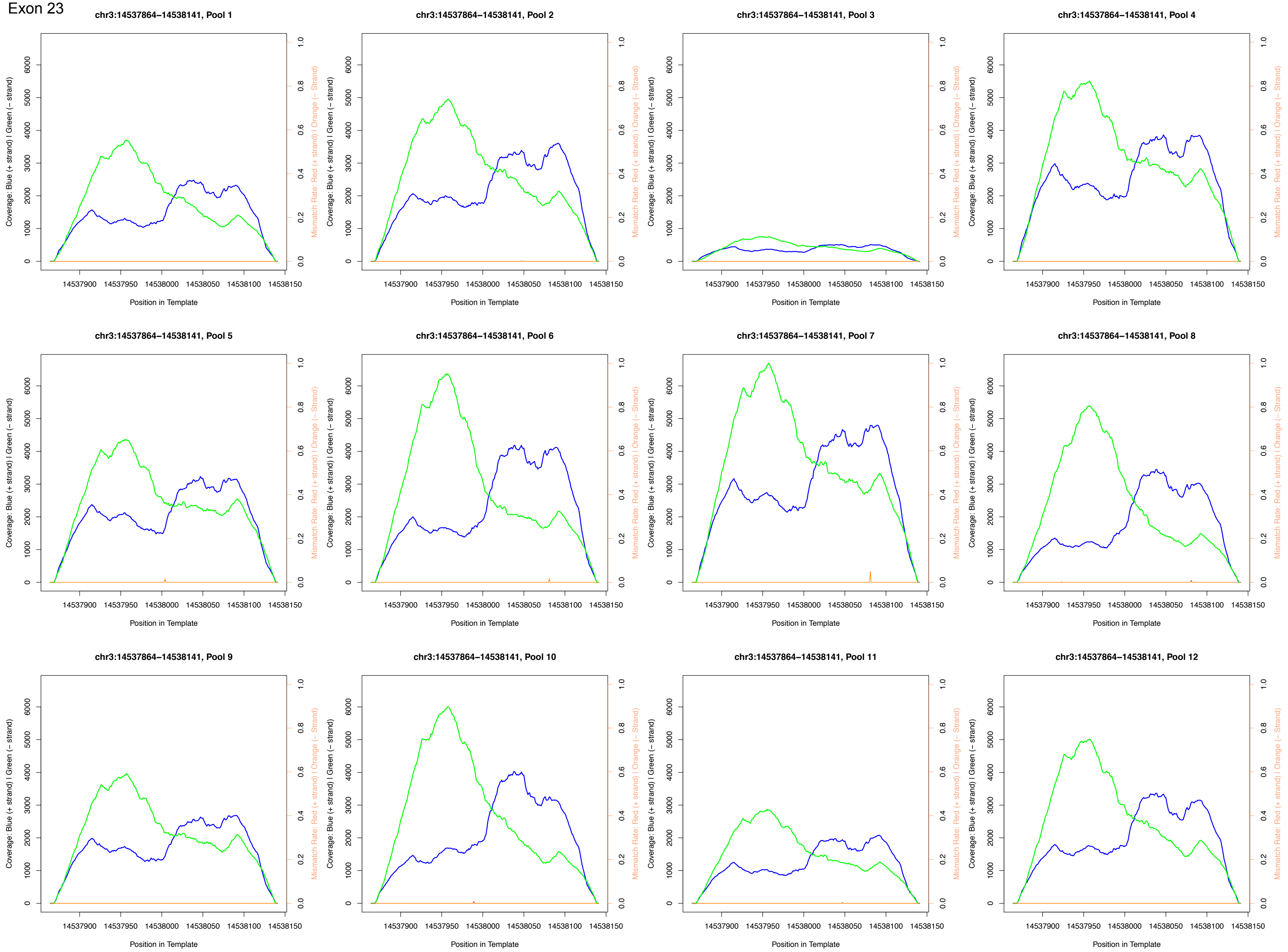
chr3:14537864–14538141, Pool 1
chr3:14537864–14538141, Pool 2
chr3:14537864–14538141, Pool 3
chr3:14537864–14538141, Pool 4
chr3:14537864–14538141, Pool 5
chr3:14537864–14538141, Pool 6
chr3:14537864–14538141, Pool 7
chr3:14537864–14538141, Pool 8
chr3:14537864–14538141, Pool 9
chr3:14537864–14538141, Pool 10
chr3:14537864–14538141, Pool 11
chr3:14537864–14538141, Pool 12
Coverage: Blue (+ strand) | Green (– strand)
Mismatch Rate: Red (+ strand) | Orange (– Strand)
Position in Template
14537900 14537950 14538000 14538050 14538100 14538150
0 1000 2000 3000 4000 5000 6000
0.0 0.2 0.4 0.6 0.8 1.0

Exon 21

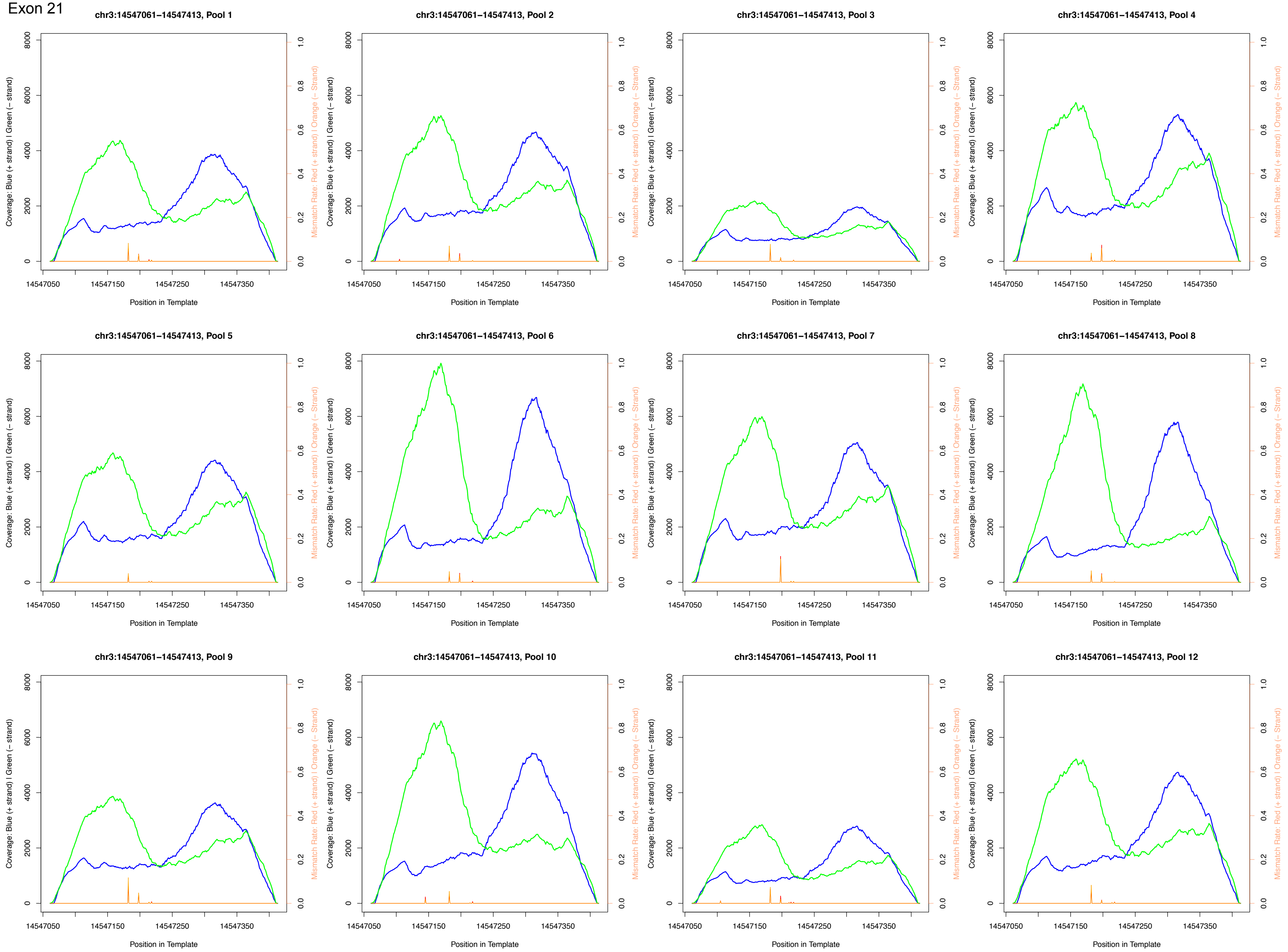
chr3:14547061–14547413, Pool 1
chr3:14547061–14547413, Pool 2
chr3:14547061–14547413, Pool 3
chr3:14547061–14547413, Pool 4
chr3:14547061–14547413, Pool 5
chr3:14547061–14547413, Pool 6
chr3:14547061–14547413, Pool 7
chr3:14547061–14547413, Pool 8
chr3:14547061–14547413, Pool 9
chr3:14547061–14547413, Pool 10
chr3:14547061–14547413, Pool 11
chr3:14547061–14547413, Pool 12
Coverage: Blue (+ strand) | Green (− strand)
Mismatch Rate: Red (+ strand) | Orange (− Strand)
Position in Template
14547050
14547150
14547250
14547350
0
2000
4000
6000
8000
0.0
0.2
0.4
0.6
0.8
1.0

Exon 20

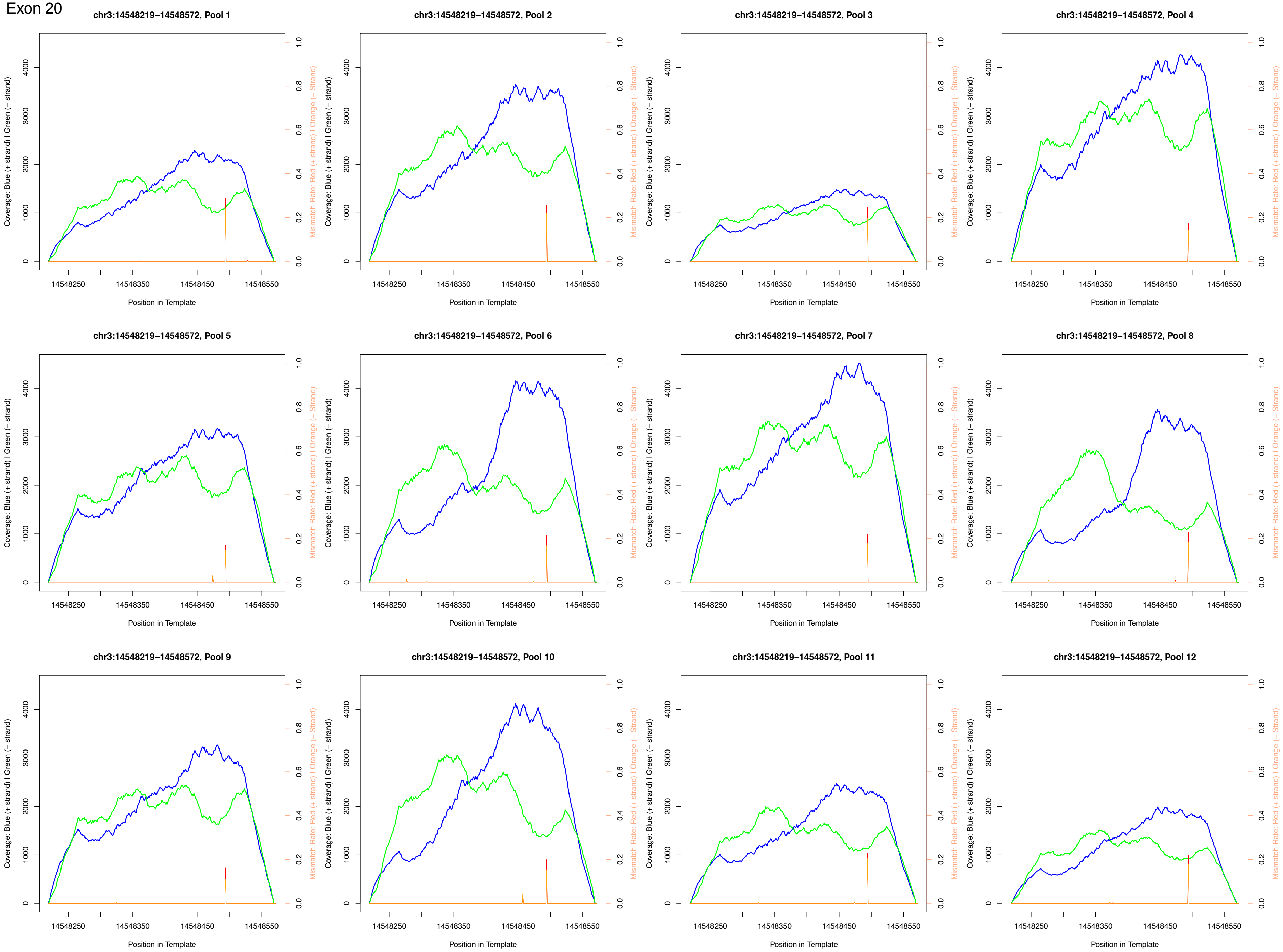

chr3:14548219–14548572, Pool 1
chr3:14548219–14548572, Pool 2
chr3:14548219–14548572, Pool 3
chr3:14548219–14548572, Pool 4
chr3:14548219–14548572, Pool 5
chr3:14548219–14548572, Pool 6
chr3:14548219–14548572, Pool 7
chr3:14548219–14548572, Pool 8
chr3:14548219–14548572, Pool 9
chr3:14548219–14548572, Pool 10
chr3:14548219–14548572, Pool 11
chr3:14548219–14548572, Pool 12
Coverage: Blue (+ strand) | Green (− strand)
Mismatch Rate: Red (+ strand) | Orange (− Strand)
Position in Template
14548250
14548350
14548450
14548550
0
1000
2000
3000
4000
0.0
0.2
0.4
0.6
0.8
1.0

Exon 19

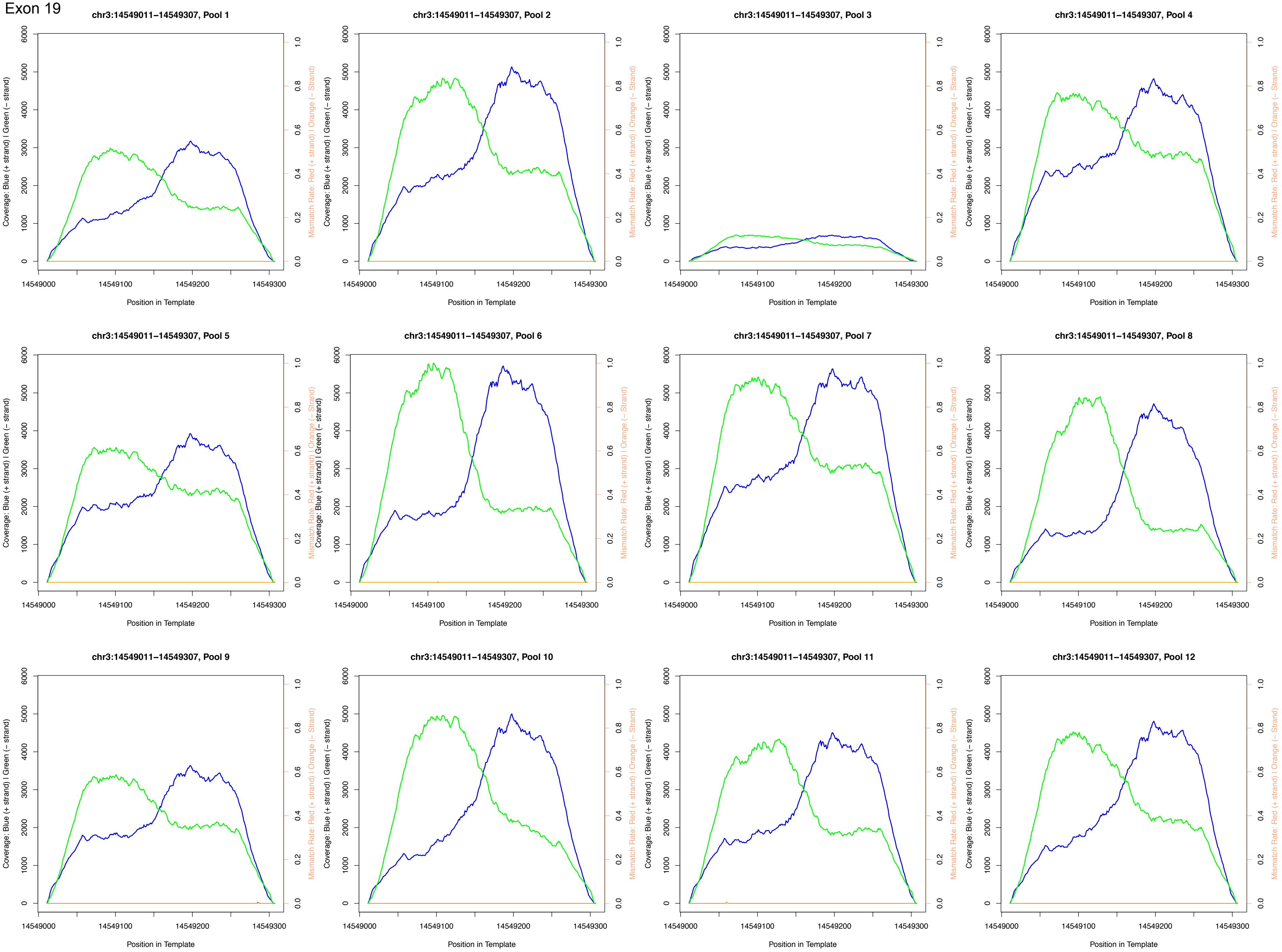
chr3:14549011–14549307, Pool 1
chr3:14549011–14549307, Pool 2
chr3:14549011–14549307, Pool 3
chr3:14549011–14549307, Pool 4
chr3:14549011–14549307, Pool 5
chr3:14549011–14549307, Pool 6
chr3:14549011–14549307, Pool 7
chr3:14549011–14549307, Pool 8
chr3:14549011–14549307, Pool 9
chr3:14549011–14549307, Pool 10
chr3:14549011–14549307, Pool 11
chr3:14549011–14549307, Pool 12
Coverage: Blue (+ strand) | Green (– strand)
Mismatch Rate: Red (+ strand) | Orange (– Strand)
Position in Template
0
1000
2000
3000
4000
5000
6000
0.0
0.2
0.4
0.6
0.8
1.0
14549000
14549100
14549200
14549300

Exon 18

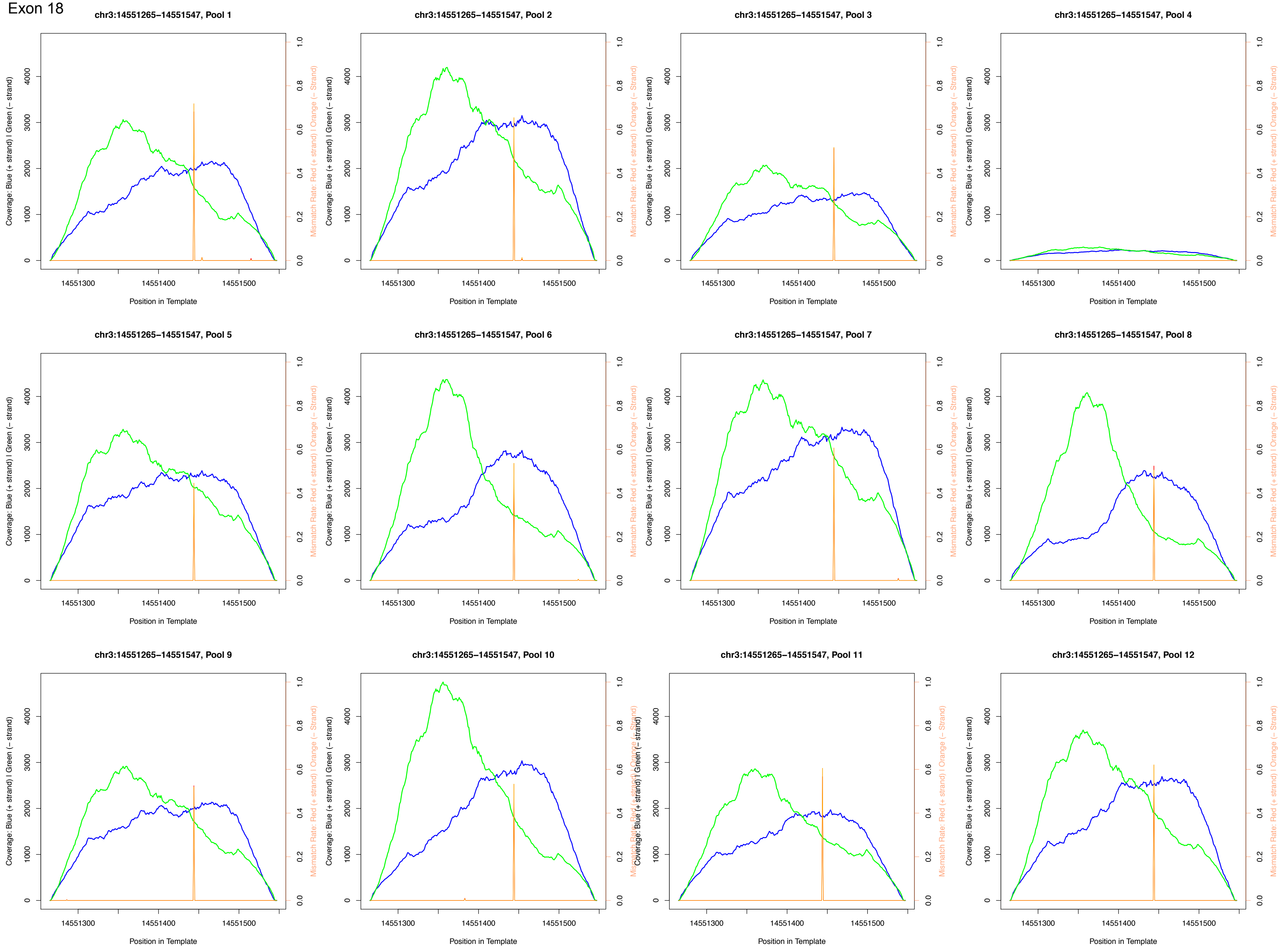

chr3:14551265–14551547, Pool 1
chr3:14551265–14551547, Pool 2
chr3:14551265–14551547, Pool 3
chr3:14551265–14551547, Pool 4
chr3:14551265–14551547, Pool 5
chr3:14551265–14551547, Pool 6
chr3:14551265–14551547, Pool 7
chr3:14551265–14551547, Pool 8
chr3:14551265–14551547, Pool 9
chr3:14551265–14551547, Pool 10
chr3:14551265–14551547, Pool 11
chr3:14551265–14551547, Pool 12
Coverage: Blue (+ strand) | Green (– strand)
Mismatch Rate: Red (+ strand) | Orange (– Strand)
Position in Template
14551300
14551400
14551500
0
1000
2000
3000
4000
0.0
0.2
0.4
0.6
0.8
1.0

Exons 16 & 17

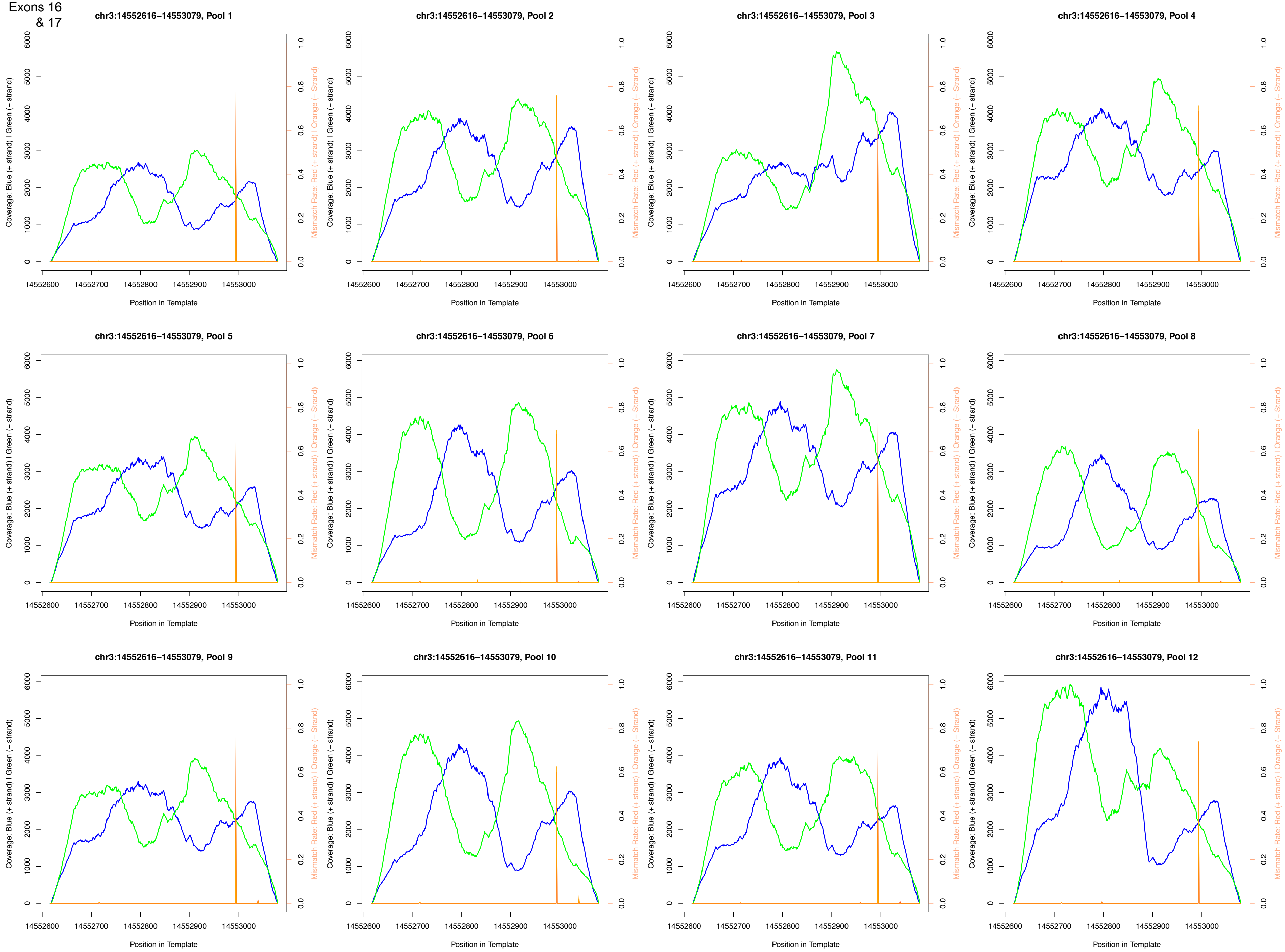
chr3:14552616–14553079, Pool 1
chr3:14552616–14553079, Pool 2
chr3:14552616–14553079, Pool 3
chr3:14552616–14553079, Pool 4
chr3:14552616–14553079, Pool 5
chr3:14552616–14553079, Pool 6
chr3:14552616–14553079, Pool 7
chr3:14552616–14553079, Pool 8
chr3:14552616–14553079, Pool 9
chr3:14552616–14553079, Pool 10
chr3:14552616–14553079, Pool 11
chr3:14552616–14553079, Pool 12
Coverage: Blue (+ strand) | Green (– strand)
Mismatch Rate: Red (+ strand) | Orange (– Strand)
Position in Template
0
1000
2000
3000
4000
5000
6000
0.0
0.2
0.4
0.6
0.8
1.0
14552600
14552700
14552800
14552900
14553000

Exon 15

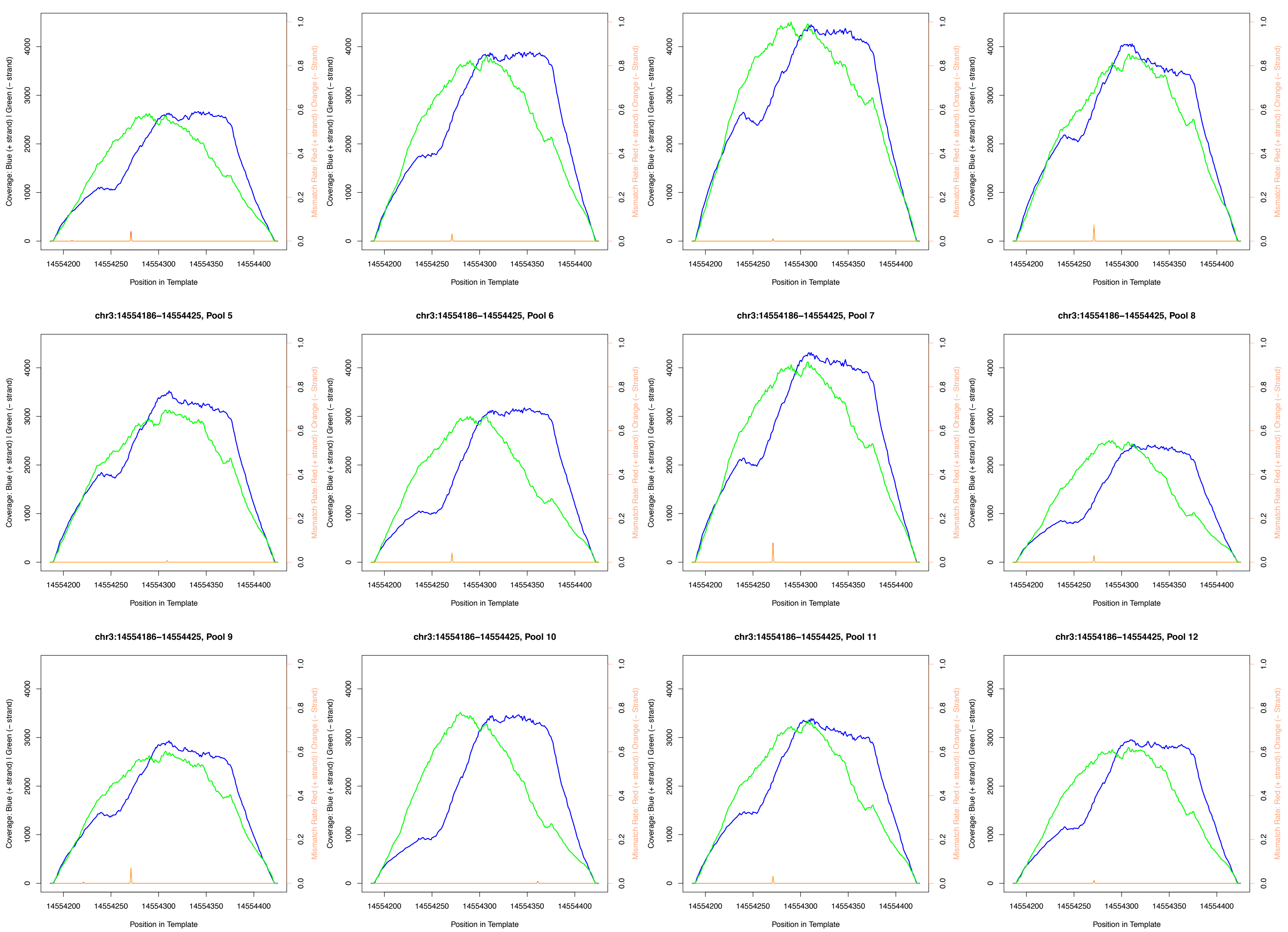
chr3:14554186–14554425, Pool 1
chr3:14554186–14554425, Pool 2
chr3:14554186–14554425, Pool 3
chr3:14554186–14554425, Pool 4
chr3:14554186–14554425, Pool 5
chr3:14554186–14554425, Pool 6
chr3:14554186–14554425, Pool 7
chr3:14554186–14554425, Pool 8
chr3:14554186–14554425, Pool 9
chr3:14554186–14554425, Pool 10
chr3:14554186–14554425, Pool 11
chr3:14554186–14554425, Pool 12
Coverage: Blue (+ strand) | Green (– strand)
Mismatch Rate: Red (+ strand) | Orange (– Strand)
Position in Template
0
1000
2000
3000
4000
0.0
0.2
0.4
0.6
0.8
1.0
14554200
14554250
14554300
14554350
14554400

Exon 14

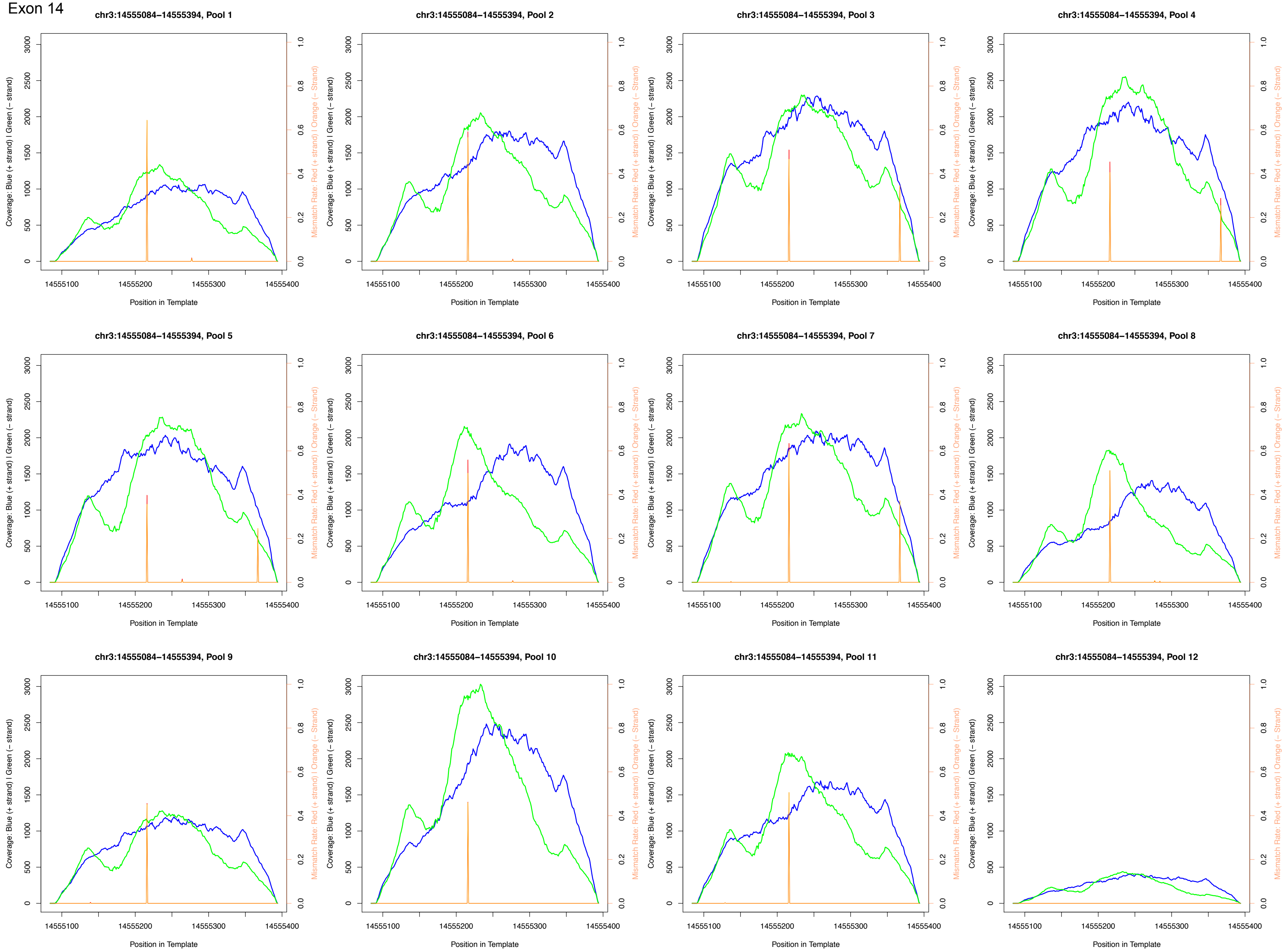
chr3:14555084–14555394, Pool 1
chr3:14555084–14555394, Pool 2
chr3:14555084–14555394, Pool 3
chr3:14555084–14555394, Pool 4
chr3:14555084–14555394, Pool 5
chr3:14555084–14555394, Pool 6
chr3:14555084–14555394, Pool 7
chr3:14555084–14555394, Pool 8
chr3:14555084–14555394, Pool 9
chr3:14555084–14555394, Pool 10
chr3:14555084–14555394, Pool 11
chr3:14555084–14555394, Pool 12
Coverage: Blue (+ strand) | Green (– strand)
Mismatch Rate: Red (+ strand) | Orange (– Strand)
Position in Template
0
500
1000
1500
2000
2500
3000
0.0
0.2
0.4
0.6
0.8
1.0
14555100
14555200
14555300
14555400

Exon 13

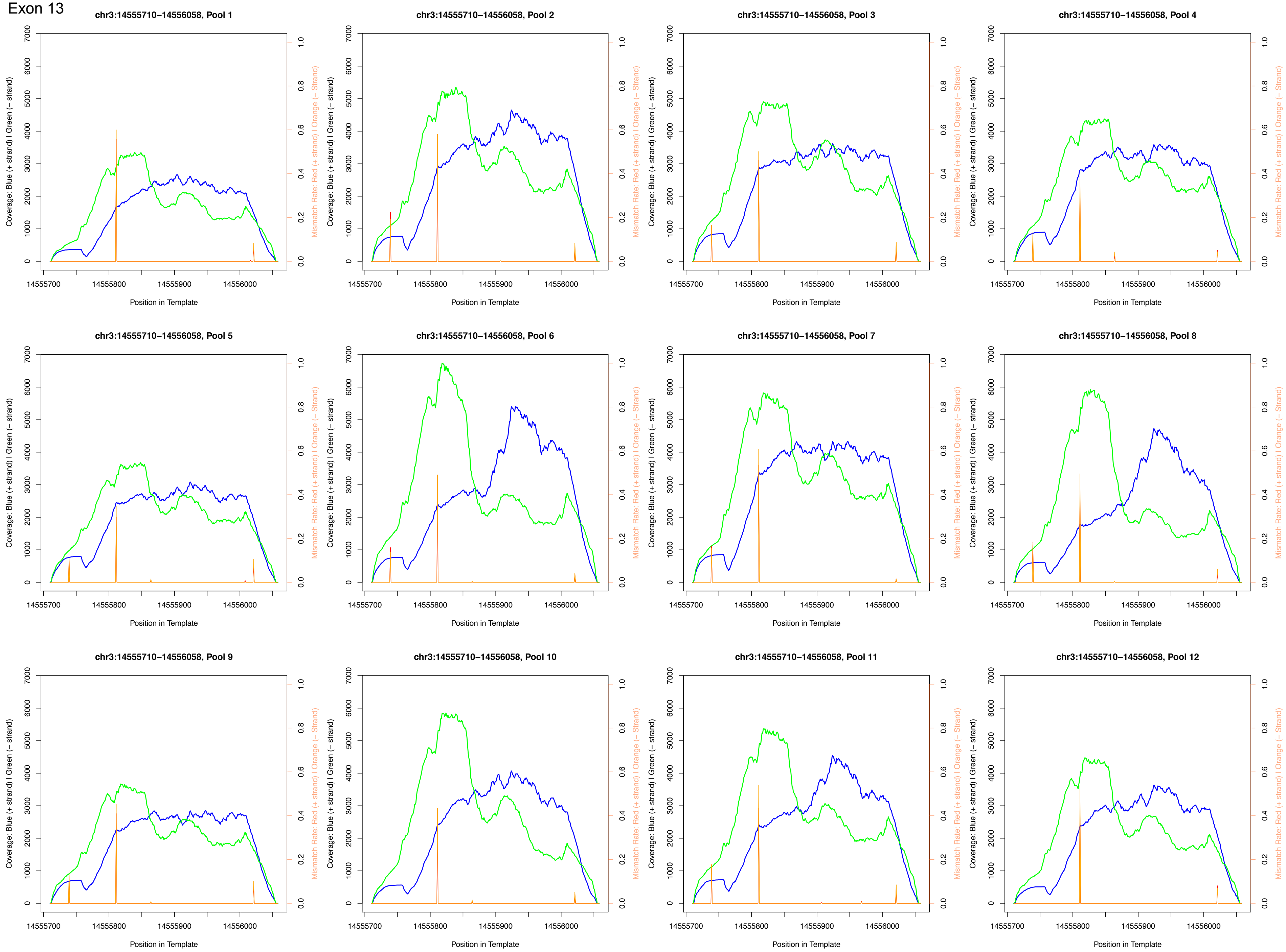
chr3:14555710–14556058, Pool 1
chr3:14555710–14556058, Pool 2
chr3:14555710–14556058, Pool 3
chr3:14555710–14556058, Pool 4
chr3:14555710–14556058, Pool 5
chr3:14555710–14556058, Pool 6
chr3:14555710–14556058, Pool 7
chr3:14555710–14556058, Pool 8
chr3:14555710–14556058, Pool 9
chr3:14555710–14556058, Pool 10
chr3:14555710–14556058, Pool 11
chr3:14555710–14556058, Pool 12
Coverage: Blue (+ strand) | Green (– strand)
Mismatch Rate: Red (+ strand) | Orange (– Strand)
Position in Template
14555700
14555800
14555900
14556000
0
1000
2000
3000
4000
5000
6000
7000
0.0
0.2
0.4
0.6
0.8
1.0

Exon 12

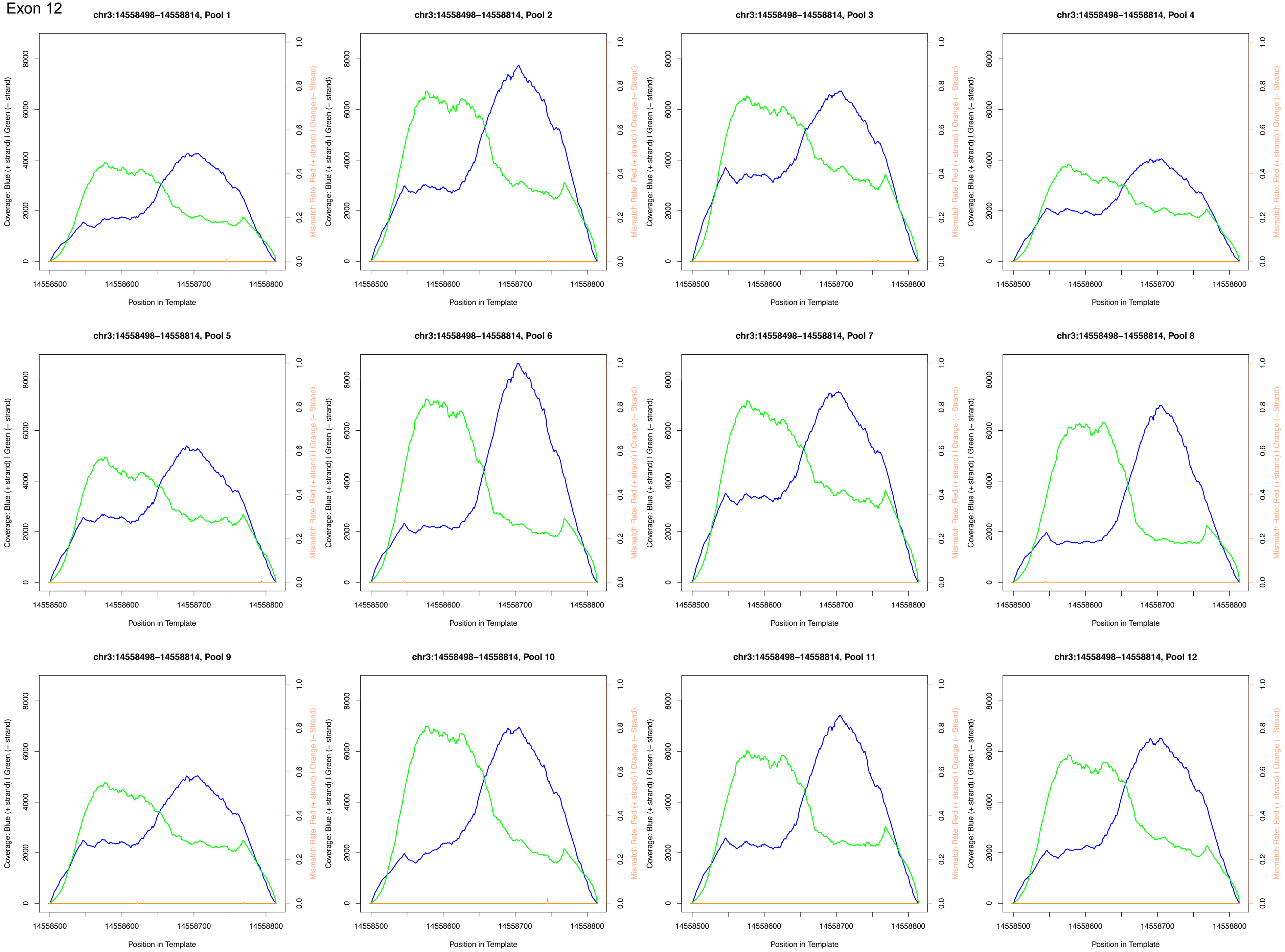
chr3:14558498–14558814, Pool 1
chr3:14558498–14558814, Pool 2
chr3:14558498–14558814, Pool 3
chr3:14558498–14558814, Pool 4
chr3:14558498–14558814, Pool 5
chr3:14558498–14558814, Pool 6
chr3:14558498–14558814, Pool 7
chr3:14558498–14558814, Pool 8
chr3:14558498–14558814, Pool 9
chr3:14558498–14558814, Pool 10
chr3:14558498–14558814, Pool 11
chr3:14558498–14558814, Pool 12
Coverage: Blue (+ strand) | Green (− strand)
Mismatch Rate: Red (+ strand) | Orange (− Strand)
Position in Template
0
2000
4000
6000
8000
0.0
0.2
0.4
0.6
0.8
1.0
14558500
14558600
14558700
14558800

Exon 11

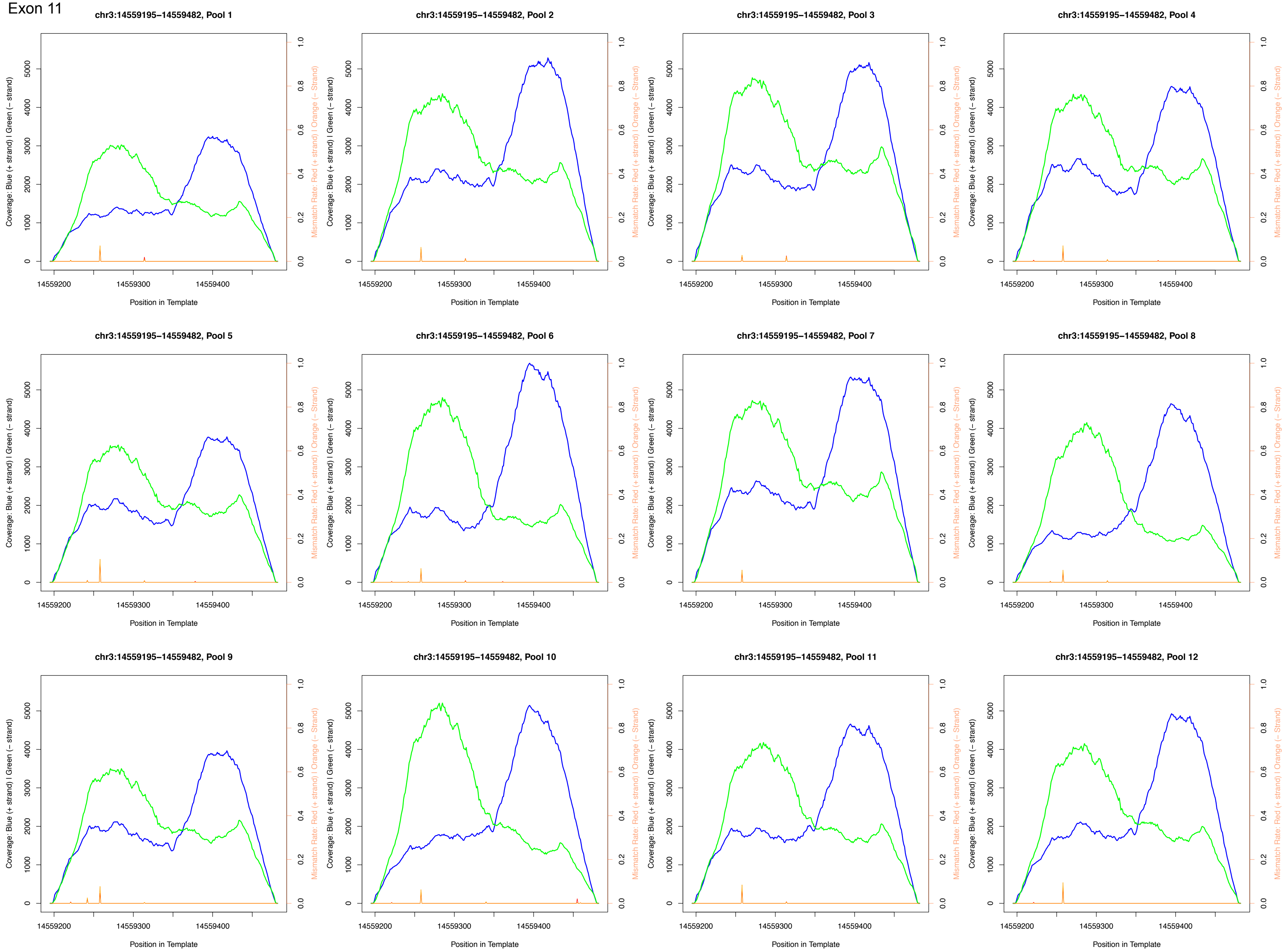
chr3:14559195–14559482, Pool 1
chr3:14559195–14559482, Pool 2
chr3:14559195–14559482, Pool 3
chr3:14559195–14559482, Pool 4
chr3:14559195–14559482, Pool 5
chr3:14559195–14559482, Pool 6
chr3:14559195–14559482, Pool 7
chr3:14559195–14559482, Pool 8
chr3:14559195–14559482, Pool 9
chr3:14559195–14559482, Pool 10
chr3:14559195–14559482, Pool 11
chr3:14559195–14559482, Pool 12
Coverage: Blue (+ strand) | Green (– strand)
Mismatch Rate: Red (+ strand) | Orange (– Strand)
Position in Template
14559200
14559300
14559400
0
1000
2000
3000
4000
5000
0.0
0.2
0.4
0.6
0.8
1.0

Exons 9 & 10

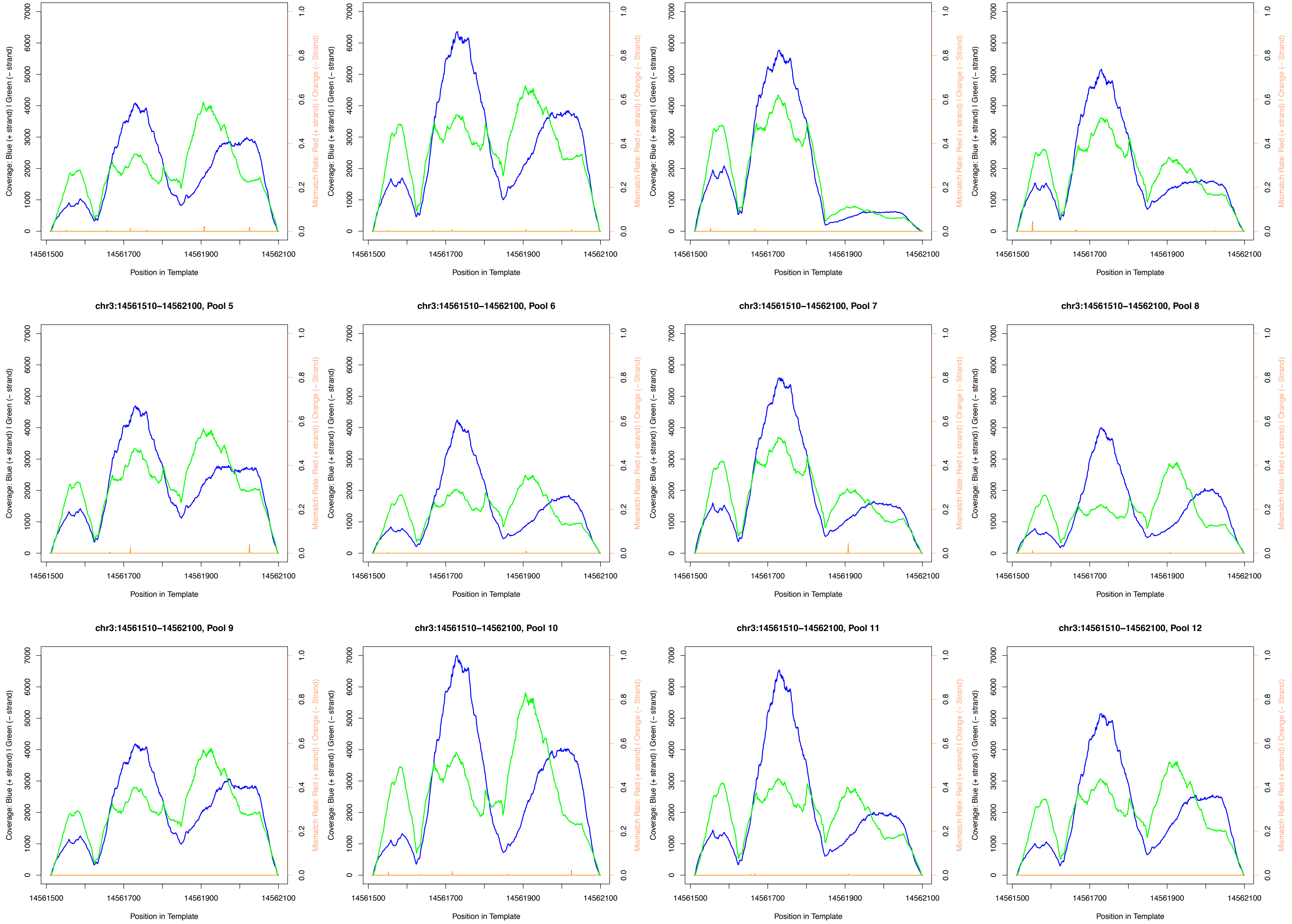

chr3:14561510–14562100, Pool 1
chr3:14561510–14562100, Pool 2
chr3:14561510–14562100, Pool 3
chr3:14561510–14562100, Pool 4
chr3:14561510–14562100, Pool 5
chr3:14561510–14562100, Pool 6
chr3:14561510–14562100, Pool 7
chr3:14561510–14562100, Pool 8
chr3:14561510–14562100, Pool 9
chr3:14561510–14562100, Pool 10
chr3:14561510–14562100, Pool 11
chr3:14561510–14562100, Pool 12
Coverage: Blue (+ strand) | Green (– strand)
Mismatch Rate: Red (+ strand) | Orange (– Strand)
Position in Template
14561500
14561700
14561900
14562100
0
1000
2000
3000
4000
5000
6000
7000
0.0
0.2
0.4
0.6
0.8
1.0

Exon 8

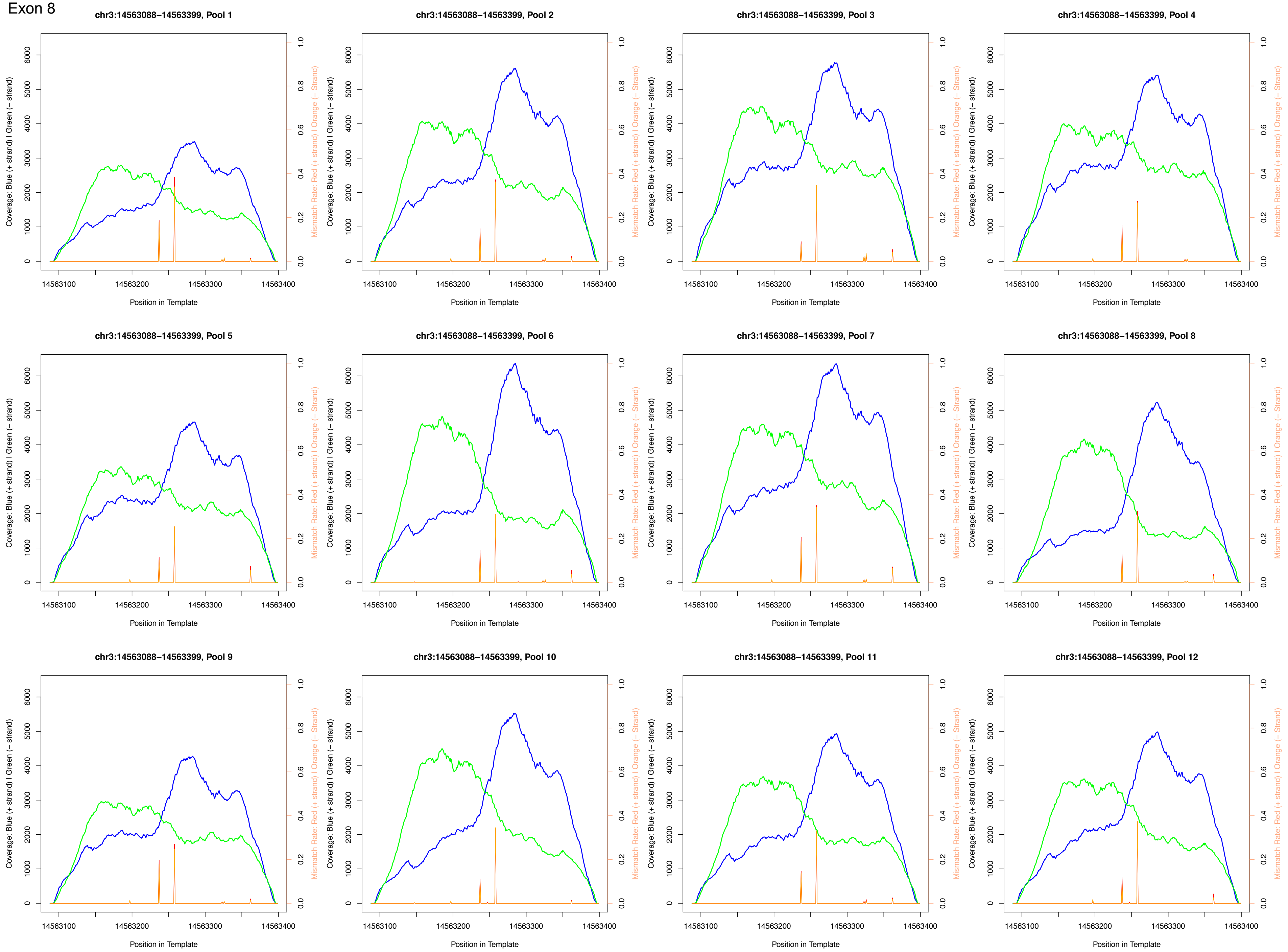
chr3:14563088–14563399, Pool 1
chr3:14563088–14563399, Pool 2
chr3:14563088–14563399, Pool 3
chr3:14563088–14563399, Pool 4
chr3:14563088–14563399, Pool 5
chr3:14563088–14563399, Pool 6
chr3:14563088–14563399, Pool 7
chr3:14563088–14563399, Pool 8
chr3:14563088–14563399, Pool 9
chr3:14563088–14563399, Pool 10
chr3:14563088–14563399, Pool 11
chr3:14563088–14563399, Pool 12
Coverage: Blue (+ strand) | Green (– strand)
Mismatch Rate: Red (+ strand) | Orange (– Strand)
Position in Template
14563100
14563200
14563300
14563400
0
1000
2000
3000
4000
5000
6000
0.0
0.2
0.4
0.6
0.8
1.0

Exon 7

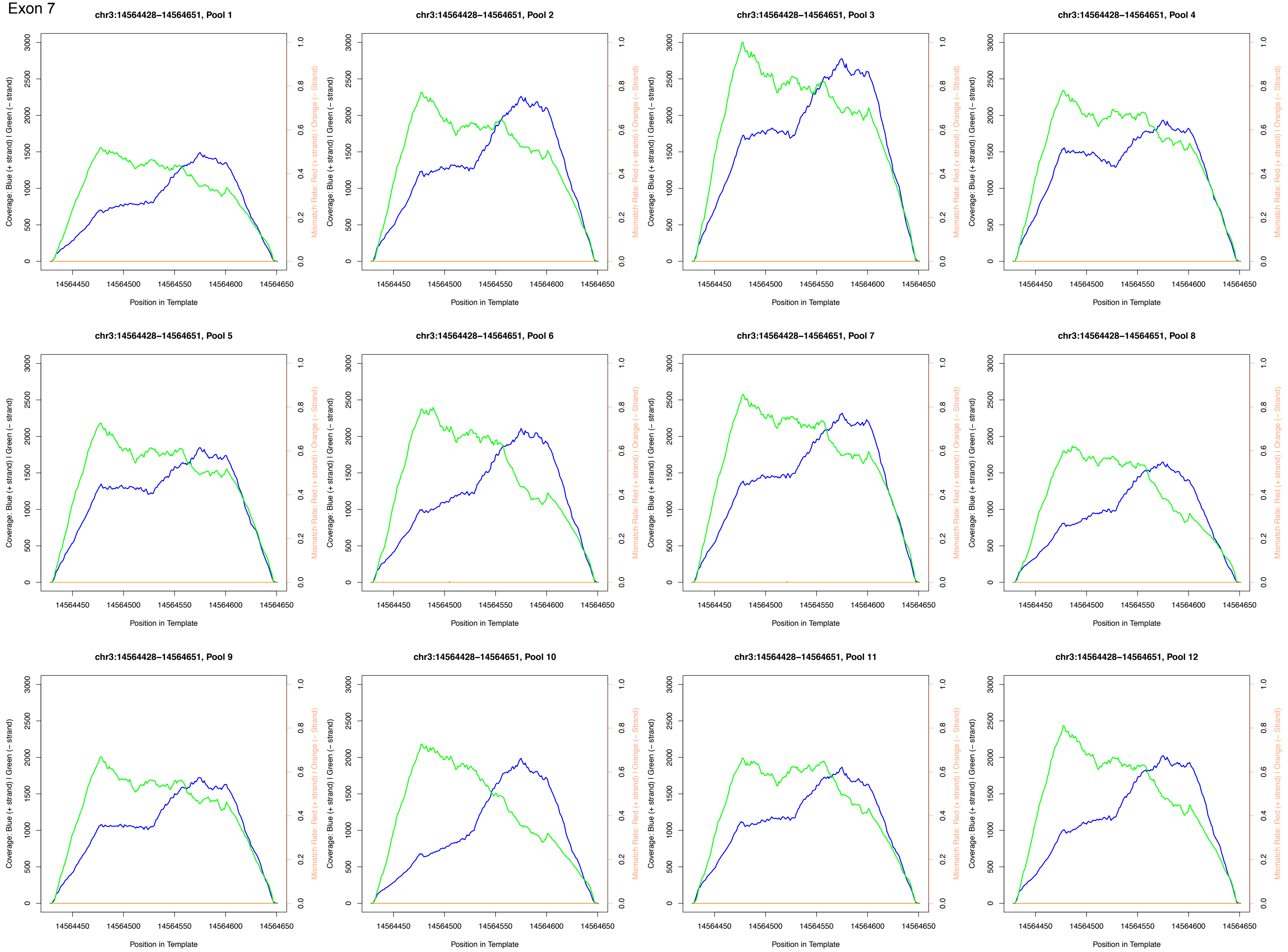
chr3:14564428–14564651, Pool 1
chr3:14564428–14564651, Pool 2
chr3:14564428–14564651, Pool 3
chr3:14564428–14564651, Pool 4
chr3:14564428–14564651, Pool 5
chr3:14564428–14564651, Pool 6
chr3:14564428–14564651, Pool 7
chr3:14564428–14564651, Pool 8
chr3:14564428–14564651, Pool 9
chr3:14564428–14564651, Pool 10
chr3:14564428–14564651, Pool 11
chr3:14564428–14564651, Pool 12
Coverage: Blue (+ strand) | Green (− strand)
Mismatch Rate: Red (+ strand) | Orange (− Strand)
Position in Template
14564450
14564500
14564550
14564600
14564650
0
500
1000
1500
2000
2500
3000
0.0
0.2
0.4
0.6
0.8
1.0

Exon 6

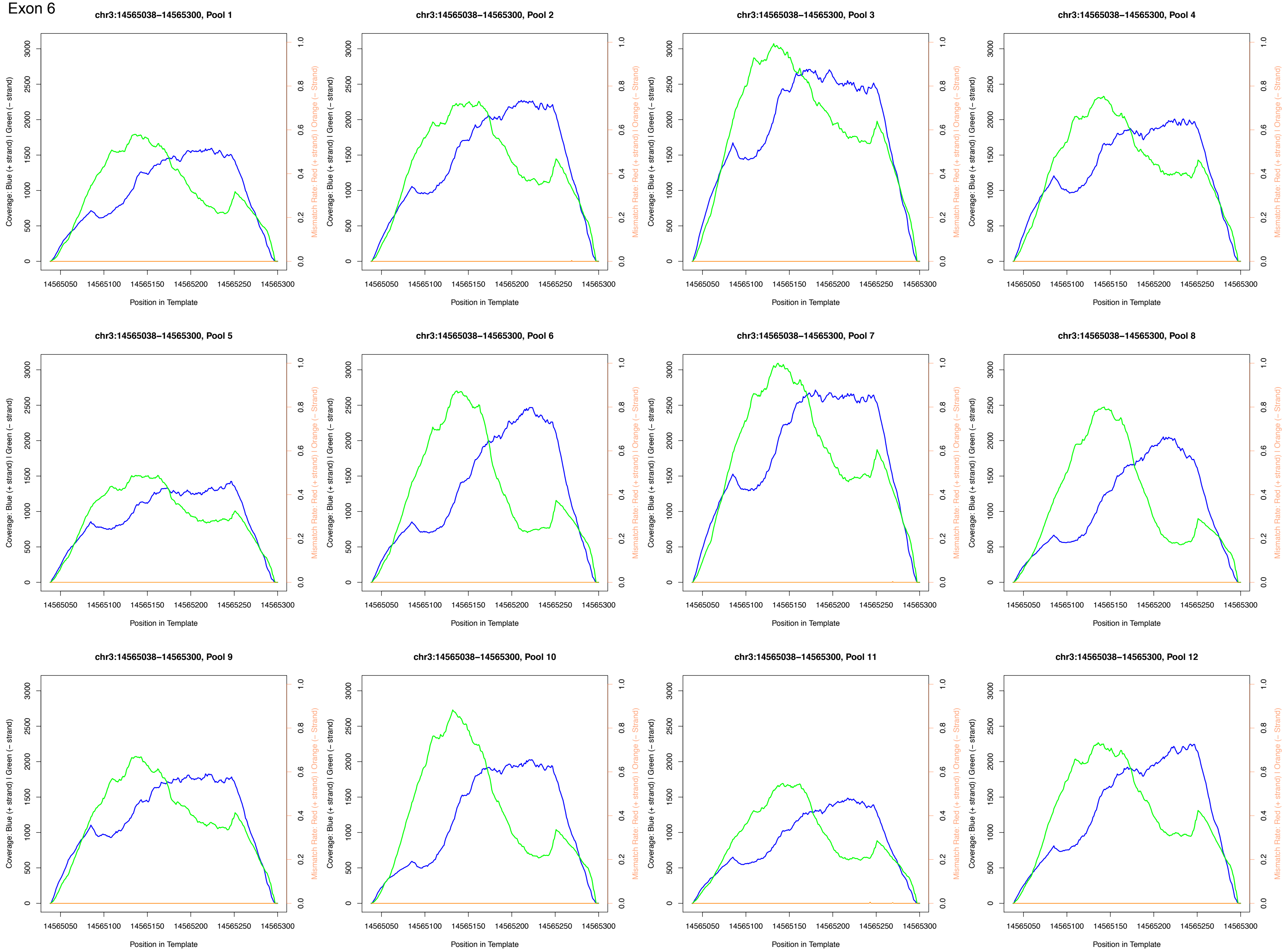

chr3:14565038–14565300, Pool 1
chr3:14565038–14565300, Pool 2
chr3:14565038–14565300, Pool 3
chr3:14565038–14565300, Pool 4
chr3:14565038–14565300, Pool 5
chr3:14565038–14565300, Pool 6
chr3:14565038–14565300, Pool 7
chr3:14565038–14565300, Pool 8
chr3:14565038–14565300, Pool 9
chr3:14565038–14565300, Pool 10
chr3:14565038–14565300, Pool 11
chr3:14565038–14565300, Pool 12
Coverage: Blue (+ strand) | Green (− strand)
Mismatch Rate: Red (+ strand) | Orange (− Strand)
Position in Template
0
500
1000
1500
2000
2500
3000
0.0
0.2
0.4
0.6
0.8
1.0
14565050
14565100
14565150
14565200
14565250
14565300

Exon 5

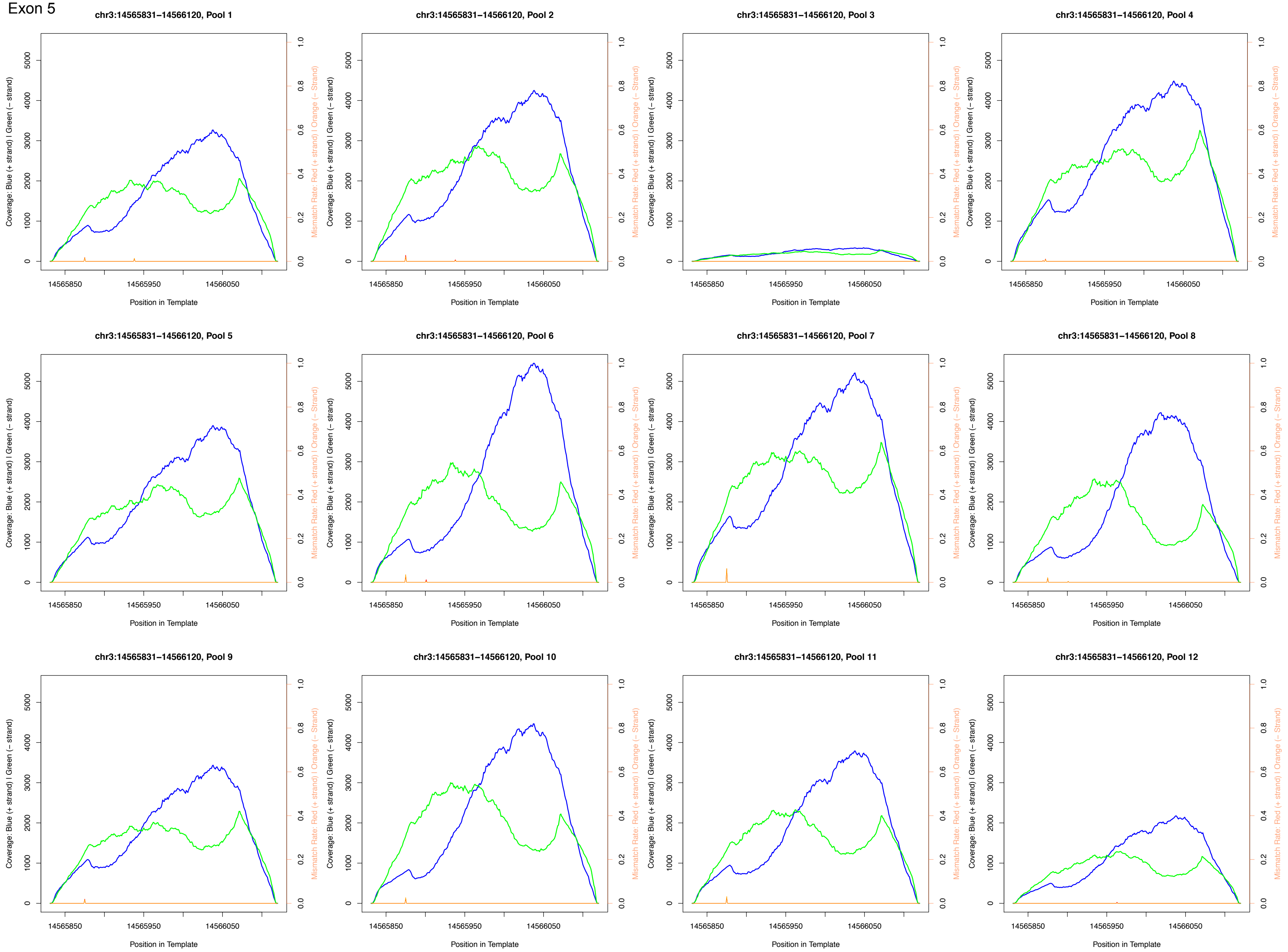
chr3:14565831–14566120, Pool 1
chr3:14565831–14566120, Pool 2
chr3:14565831–14566120, Pool 3
chr3:14565831–14566120, Pool 4
chr3:14565831–14566120, Pool 5
chr3:14565831–14566120, Pool 6
chr3:14565831–14566120, Pool 7
chr3:14565831–14566120, Pool 8
chr3:14565831–14566120, Pool 9
chr3:14565831–14566120, Pool 10
chr3:14565831–14566120, Pool 11
chr3:14565831–14566120, Pool 12
Coverage: Blue (+ strand) | Green (– strand)
Mismatch Rate: Red (+ strand) | Orange (– Strand)
Position in Template
14565850
14565950
14566050
0
1000
2000
3000
4000
5000
0.0
0.2
0.4
0.6
0.8
1.0

Exon 4

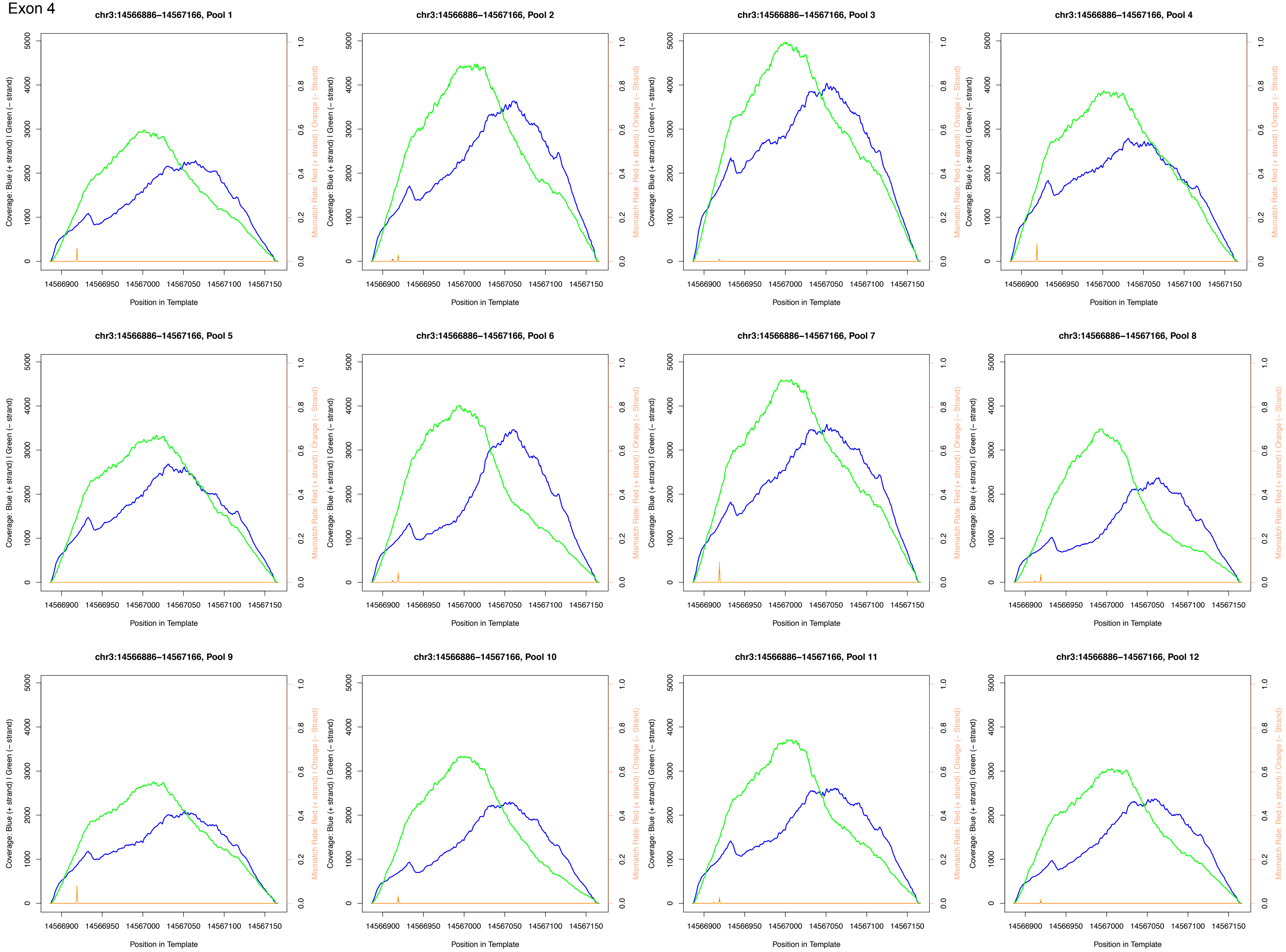
chr3:14566886–14567166, Pool 1
chr3:14566886–14567166, Pool 2
chr3:14566886–14567166, Pool 3
chr3:14566886–14567166, Pool 4
chr3:14566886–14567166, Pool 5
chr3:14566886–14567166, Pool 6
chr3:14566886–14567166, Pool 7
chr3:14566886–14567166, Pool 8
chr3:14566886–14567166, Pool 9
chr3:14566886–14567166, Pool 10
chr3:14566886–14567166, Pool 11
chr3:14566886–14567166, Pool 12
Coverage: Blue (+ strand) | Green (− strand)
Mismatch Rate: Red (+ strand) | Orange (− Strand)
Position in Template
0
1000
2000
3000
4000
5000
0.0
0.2
0.4
0.6
0.8
1.0
14566900
14566950
14567000
14567050
14567100
14567150

Exon 3

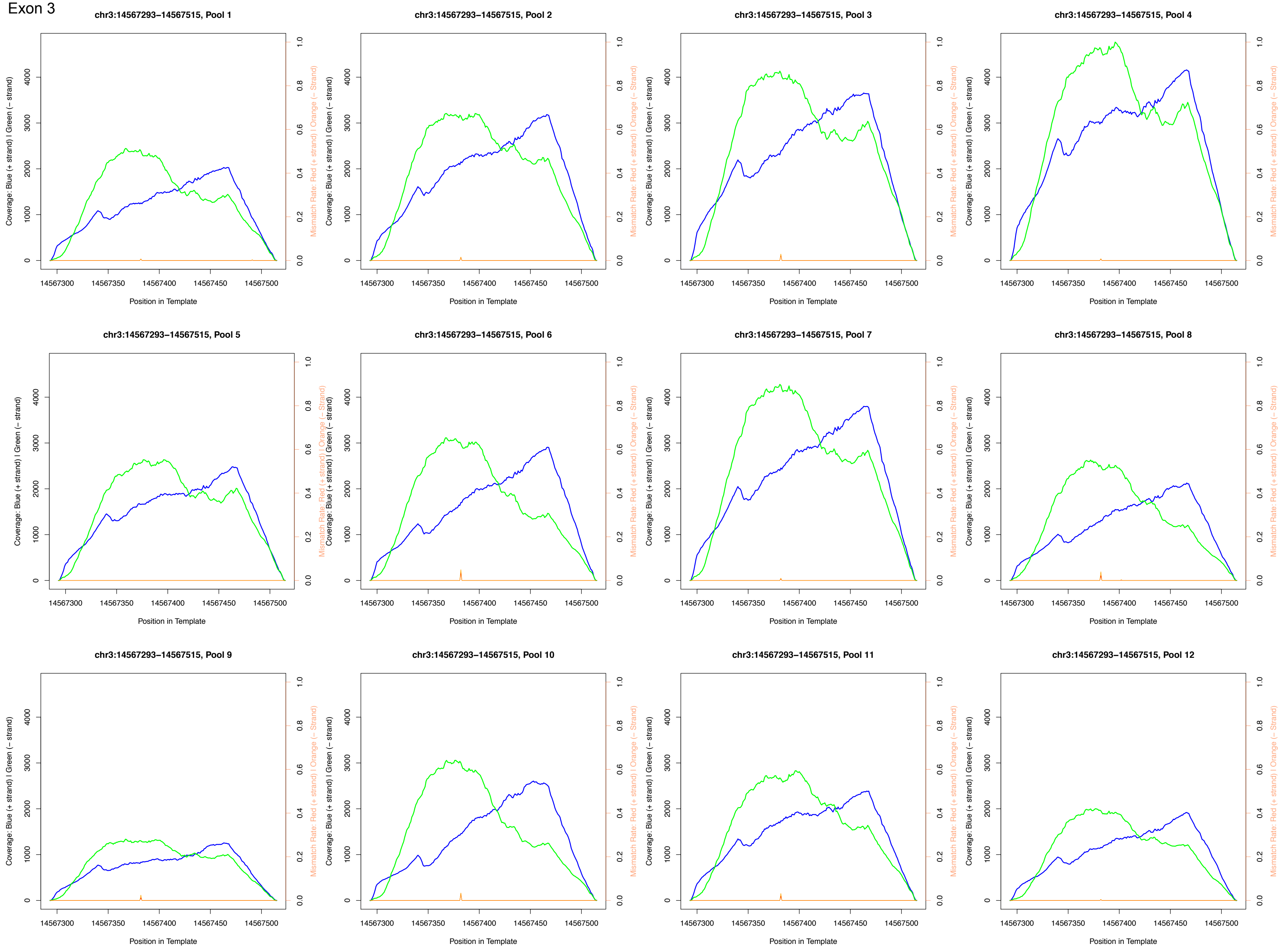
chr3:14567293–14567515, Pool 1
chr3:14567293–14567515, Pool 2
chr3:14567293–14567515, Pool 3
chr3:14567293–14567515, Pool 4
chr3:14567293–14567515, Pool 5
chr3:14567293–14567515, Pool 6
chr3:14567293–14567515, Pool 7
chr3:14567293–14567515, Pool 8
chr3:14567293–14567515, Pool 9
chr3:14567293–14567515, Pool 10
chr3:14567293–14567515, Pool 11
chr3:14567293–14567515, Pool 12
Coverage: Blue (+ strand) | Green (− strand)
Mismatch Rate: Red (+ strand) | Orange (− Strand)
Position in Template
14567300
14567350
14567400
14567450
14567500
0
1000
2000
3000
4000
0.0
0.2
0.4
0.6
0.8
1.0

Exon 2

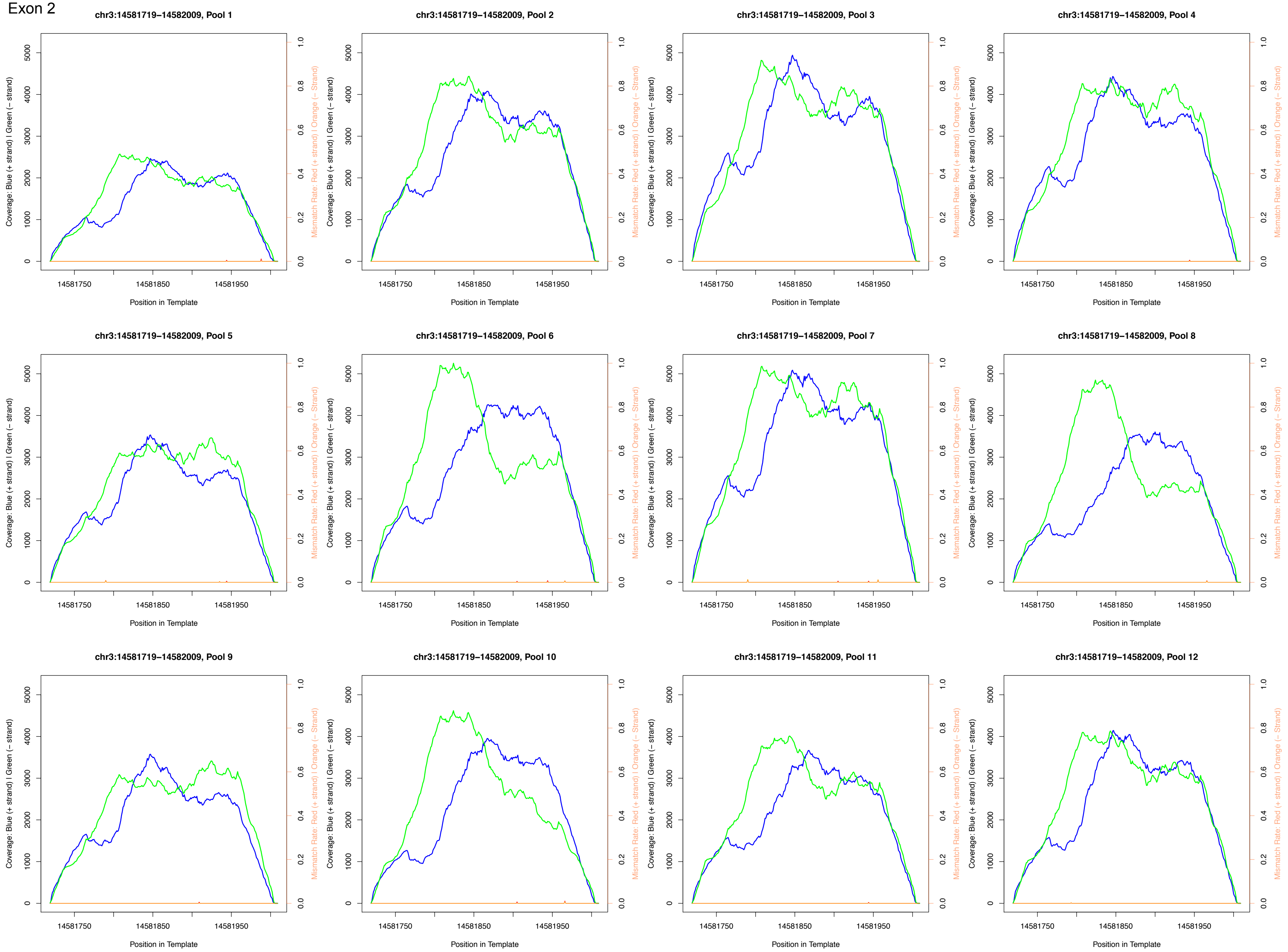
chr3:14581719–14582009, Pool 1
chr3:14581719–14582009, Pool 2
chr3:14581719–14582009, Pool 3
chr3:14581719–14582009, Pool 4
chr3:14581719–14582009, Pool 5
chr3:14581719–14582009, Pool 6
chr3:14581719–14582009, Pool 7
chr3:14581719–14582009, Pool 8
chr3:14581719–14582009, Pool 9
chr3:14581719–14582009, Pool 10
chr3:14581719–14582009, Pool 11
chr3:14581719–14582009, Pool 12
Coverage: Blue (+ strand) | Green (− strand)
Mismatch Rate: Red (+ strand) | Orange (− Strand)
Position in Template
14581750
14581850
14581950
0
1000
2000
3000
4000
5000
0.0
0.2
0.4
0.6
0.8
1.0

Exon 1

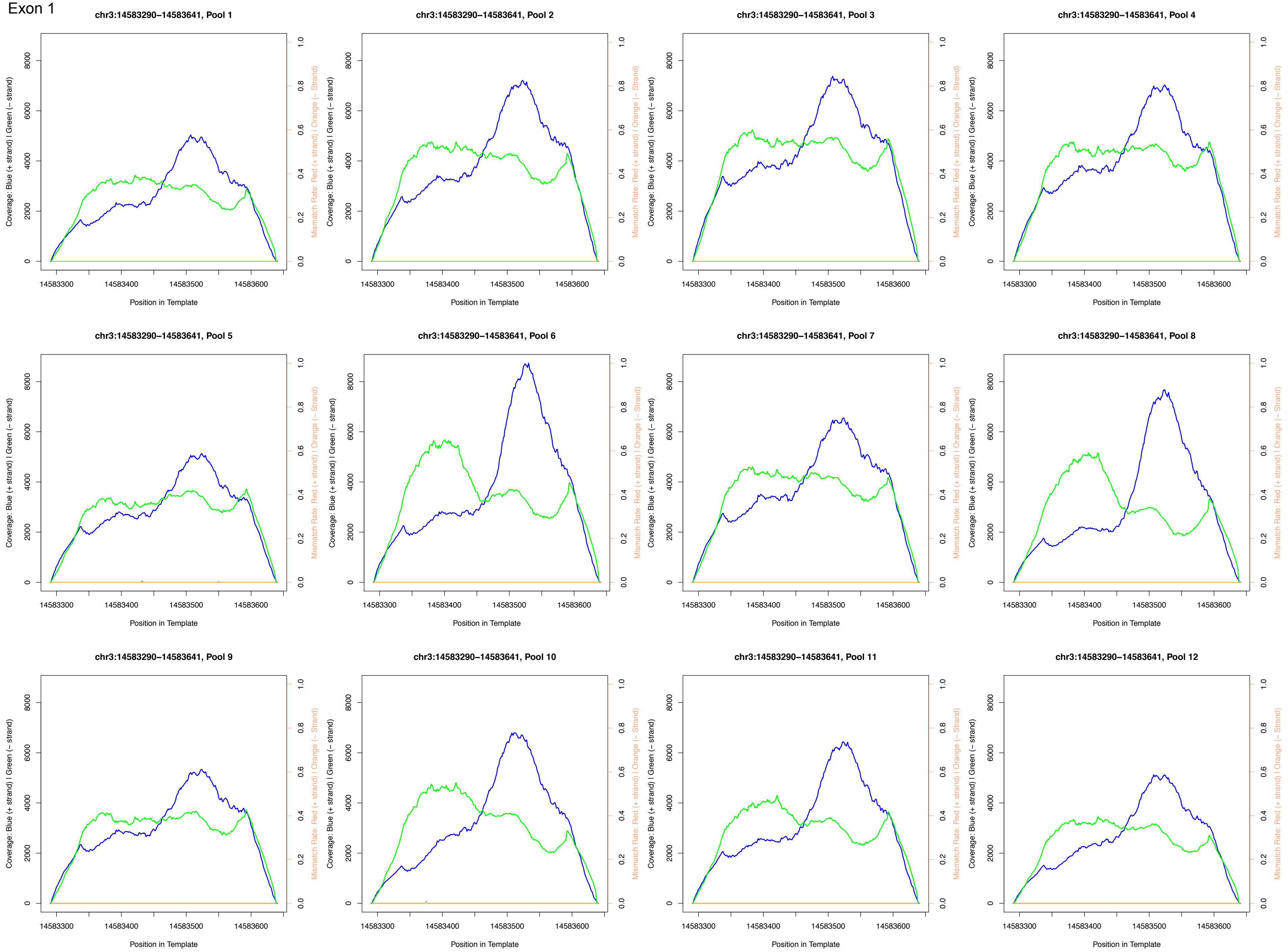

chr3:14583290–14583641, Pool 1
chr3:14583290–14583641, Pool 2
chr3:14583290–14583641, Pool 3
chr3:14583290–14583641, Pool 4
chr3:14583290–14583641, Pool 5
chr3:14583290–14583641, Pool 6
chr3:14583290–14583641, Pool 7
chr3:14583290–14583641, Pool 8
chr3:14583290–14583641, Pool 9
chr3:14583290–14583641, Pool 10
chr3:14583290–14583641, Pool 11
chr3:14583290–14583641, Pool 12
Coverage: Blue (+ strand) | Green (− strand)
Mismatch Rate: Red (+ strand) | Orange (− Strand)
Position in Template
14583300
14583400
14583500
14583600
0
2000
4000
6000
8000
0.0
0.2
0.4
0.6
0.8
1.0
