# Supplementary material for: Effective detection of rare variants in pooled DNA samples using Cross-pool tailcurve analysis
Source: Genome Biol. 2011 Sep 28;12(9):R93. doi: 10.1186/gb-2011-12-9-r93 (PMC3308056; doi:10.1186/gb-2011-12-9-r93)
Supplement: Additional file 3 — Diagrammatic output of first three filtering steps using SERVIC4E on first cohort data. Left-hand panel uses Illumina base calls. Right-hand panel uses Srfim base calls. Individual filtering steps progress while moving down each panel. Colored dots incorporate validation data for visualization purposes; blue dots are valid variant pools and red dots are invalid variant pools. Within each panel, the graphs on the left are Average quality versus Weighted allele frequency distributions. X-axis is average Phred quality for each variant-pool. Y-axis is log10 of weighted allele frequency. Histograms on the right depict the frequency of evaluated tailcurve ratios across bins of length = 2. [file gb-2011-12-9-r93-S3.PDF]

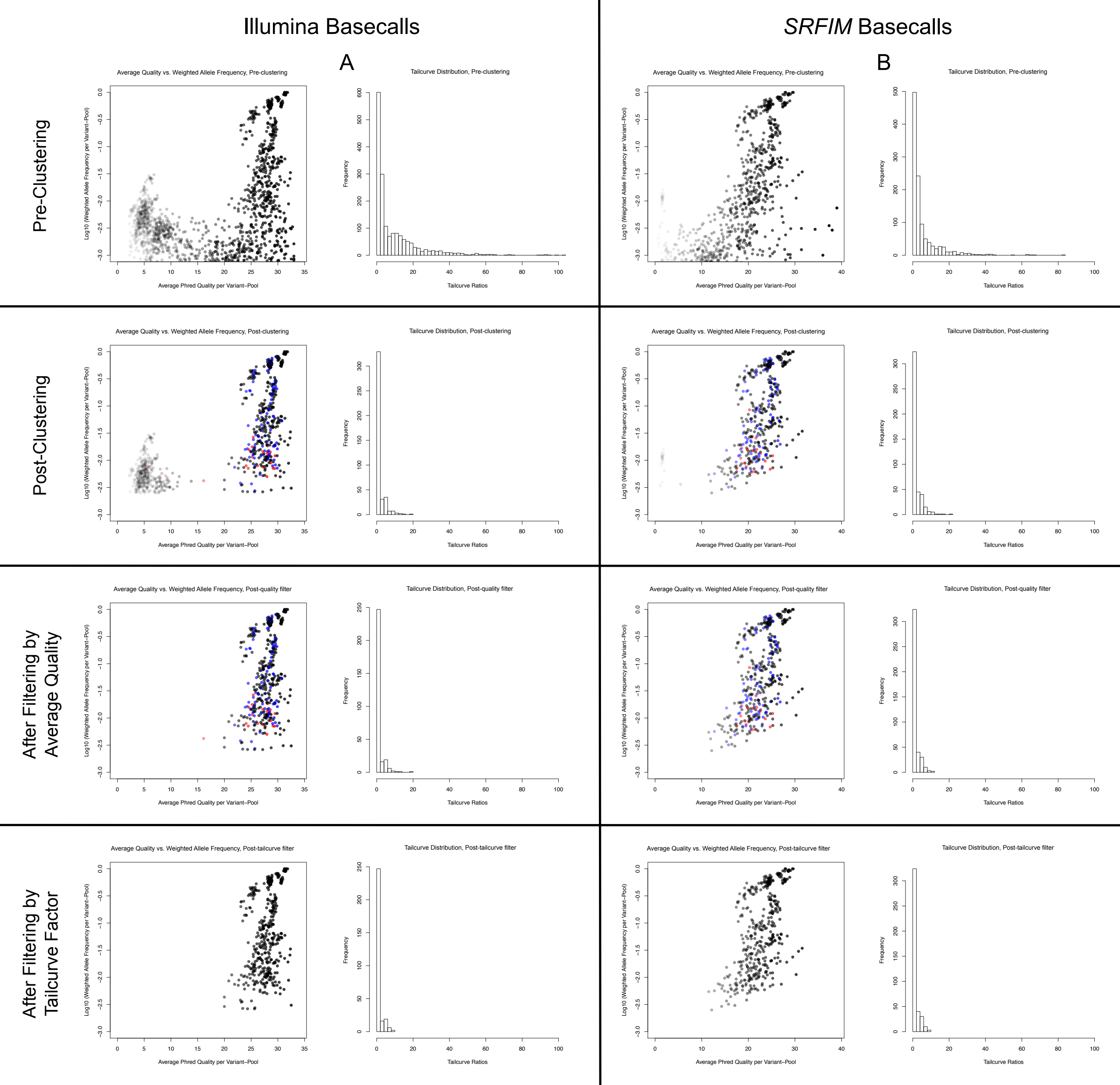

Illumina Basecalls
SRFIM Basecalls
A
B
Pre-Clustering
Post-Clustering
After Filtering by Average Quality
After Filtering by Tailcurve Factor
Average Quality vs. Weighted Allele Frequency, Pre-clustering
Tailcurve Distribution, Pre-clustering
Average Quality vs. Weighted Allele Frequency, Post-clustering
Tailcurve Distribution, Post-clustering
Average Quality vs. Weighted Allele Frequency, Post-quality filter
Tailcurve Distribution, Post-quality filter
Average Quality vs. Weighted Allele Frequency, Post-tailcurve filter
Tailcurve Distribution, Post-tailcurve filter
Log10 (Weighted Allele Frequency per Variant–Pool)
Average Phred Quality per Variant–Pool
Frequency
Tailcurve Ratios
