# Supplementary figures and images for: Effective detection of rare variants in pooled DNA samples using Cross-pool tailcurve analysis
Source: Genome Biol. 2011 Sep 28;12(9):R93. doi: 10.1186/gb-2011-12-9-r93 (PMC3308056; doi:10.1186/gb-2011-12-9-r93)

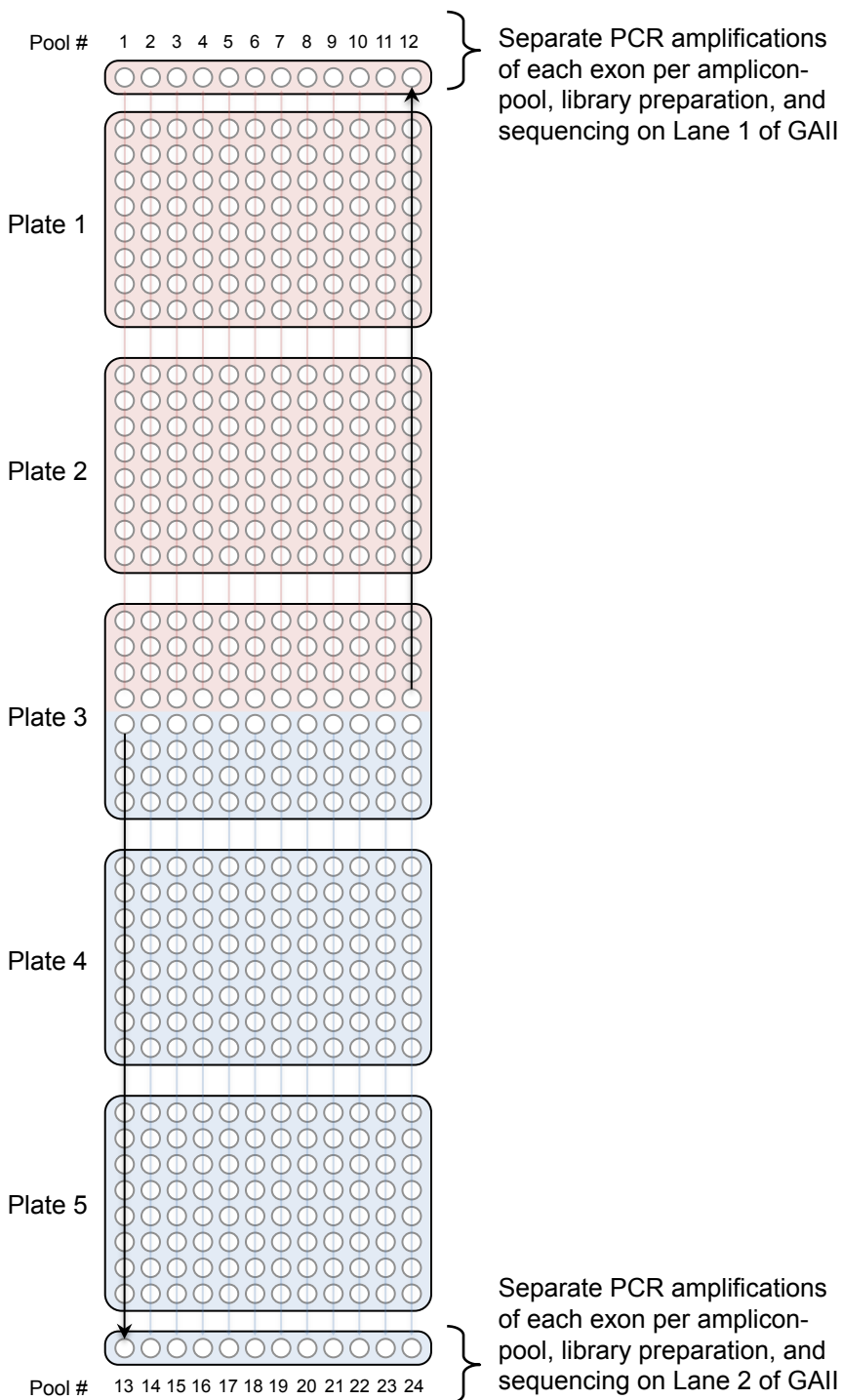

Supplement: Additional file 9 — Pooling strategy for second cohort samples. Example: Normalized DNA samples from column 12 of plates 1 and 2 as well as samples from plate 3, column 12, rows A, B, C, and D are pooled together to form pool 12. Normalized DNA samples from column 1 of plates 4 and 5 as well as samples from plate 3, column 1, rows E, F, G, and H are pooled together to form pool 13. [file gb-2011-12-9-r93-S9.PDF]
