# Supplementary material for: Effective detection of rare variants in pooled DNA samples using Cross-pool tailcurve analysis
Source: Genome Biol. 2011 Sep 28;12(9):R93. doi: 10.1186/gb-2011-12-9-r93 (PMC3308056; doi:10.1186/gb-2011-12-9-r93)
Supplement: Additional file 10 — Effect of strict alignment on coverage from concatenated amplicons. Panel 1 indicates targets for amplification (primers denoted by black half-arrows). Color-coding for each unique target region is retained in all panels. Panel 2 depicts ligation (concatenation) of amplicons. Only two amplicons are depicted; in practice many amplicons ligate together in a row. Darker shaded regions are from primer sequence. Panel 3 depicts random fragmentation to generate 150- to 200-bp segments for sequencing. Panel 4 depicts subsequent strict alignment of short (left) and long (right) reads to genomic reference sequence. [file gb-2011-12-9-r93-S10.PDF]

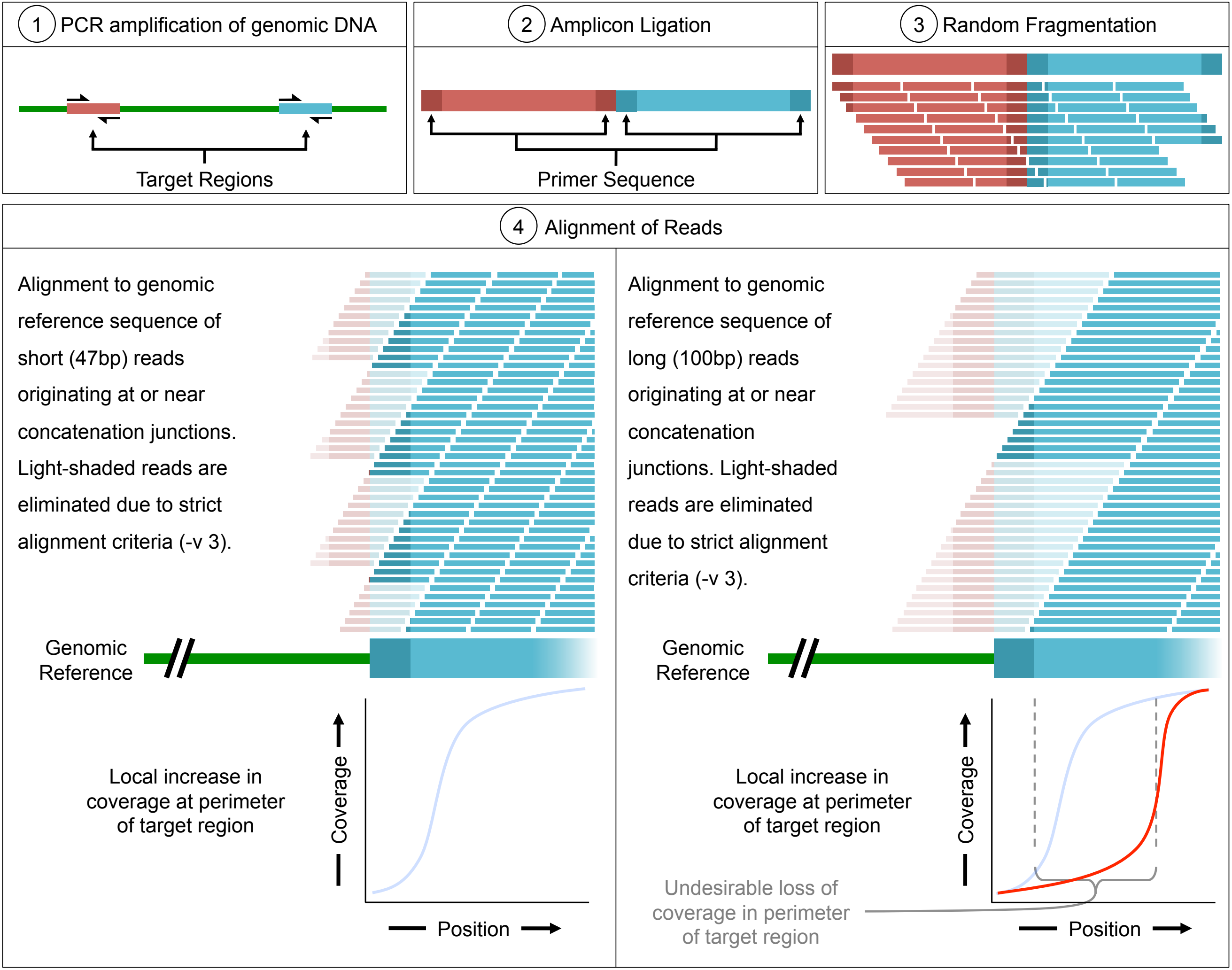
1 PCR amplification of genomic DNA
Target Regions
2 Amplicon Ligation
Primer Sequence
3 Random Fragmentation
4 Alignment of Reads
Alignment to genomic reference sequence of short (47bp) reads originating at or near concatenation junctions. Light-shaded reads are eliminated due to strict alignment criteria (-v 3).
Genomic Reference
Local increase in coverage at perimeter of target region
Coverage
Position
Alignment to genomic reference sequence of long (100bp) reads originating at or near concatenation junctions. Light-shaded reads are eliminated due to strict alignment criteria (-v 3).
Genomic Reference
Local increase in coverage at perimeter of target region
Undesirable loss of coverage in perimeter of target region
Coverage
Position
