# Supplementary material for: Effective detection of rare variants in pooled DNA samples using Cross-pool tailcurve analysis
Source: Genome Biol. 2011 Sep 28;12(9):R93. doi: 10.1186/gb-2011-12-9-r93 (PMC3308056; doi:10.1186/gb-2011-12-9-r93)
Supplement: Additional file 11 — Depth of coverage for each amplicon pool derived from second cohort sequencing data. Blue line depicts absolute coverage for plus-strand aligned reads. Green line depicts coverage of minus-strand aligned reads. Scales of X- and Y-axes are identical for all graphs depicted for each exon. Light red line indicates presumptive mismatch rate determined from plus-strand aligned reads. Light orange line indicates presumptive mismatch rate determined from minus-strand aligned reads. Ratio of mismatch rate between plus and minus strands is later incorporated into the tailcurve factor used in filtering by SERVIC4E. [file gb-2011-12-9-r93-S11.PDF]

Exon 25

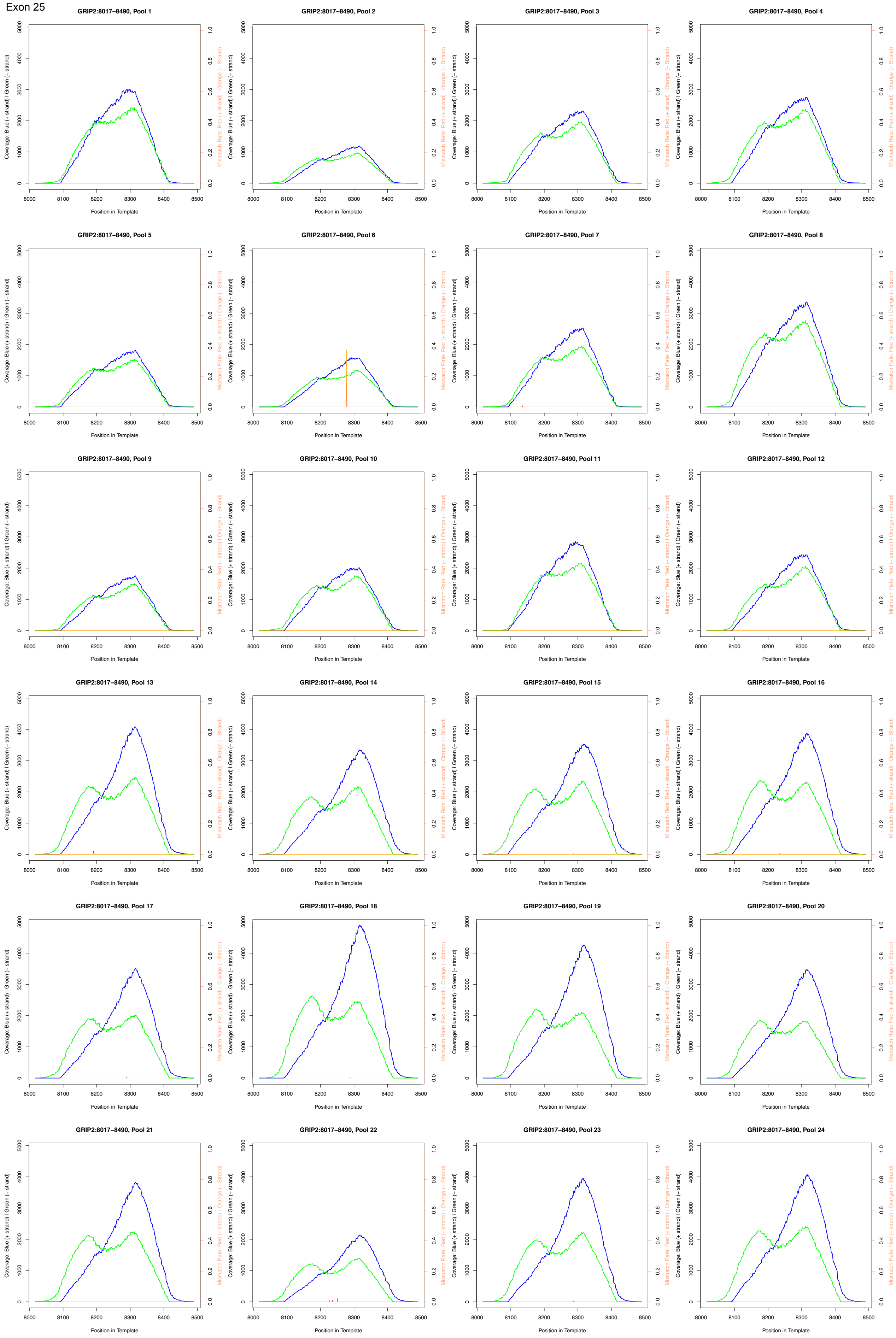
GRIP2:8017–8490, Pool 1
GRIP2:8017–8490, Pool 2
GRIP2:8017–8490, Pool 3
GRIP2:8017–8490, Pool 4
GRIP2:8017–8490, Pool 5
GRIP2:8017–8490, Pool 6
GRIP2:8017–8490, Pool 7
GRIP2:8017–8490, Pool 8
GRIP2:8017–8490, Pool 9
GRIP2:8017–8490, Pool 10
GRIP2:8017–8490, Pool 11
GRIP2:8017–8490, Pool 12
GRIP2:8017–8490, Pool 13
GRIP2:8017–8490, Pool 14
GRIP2:8017–8490, Pool 15
GRIP2:8017–8490, Pool 16
GRIP2:8017–8490, Pool 17
GRIP2:8017–8490, Pool 18
GRIP2:8017–8490, Pool 19
GRIP2:8017–8490, Pool 20
GRIP2:8017–8490, Pool 21
GRIP2:8017–8490, Pool 22
GRIP2:8017–8490, Pool 23
GRIP2:8017–8490, Pool 24
Coverage: Blue (+ strand) | Green (− strand)
Mismatch Rate: Red (+ strand) | Orange (− Strand)
Position in Template
0
1000
2000
3000
4000
5000
0.0
0.2
0.4
0.6
0.8
1.0
8000
8100
8200
8300
8400
8500

Exon 24

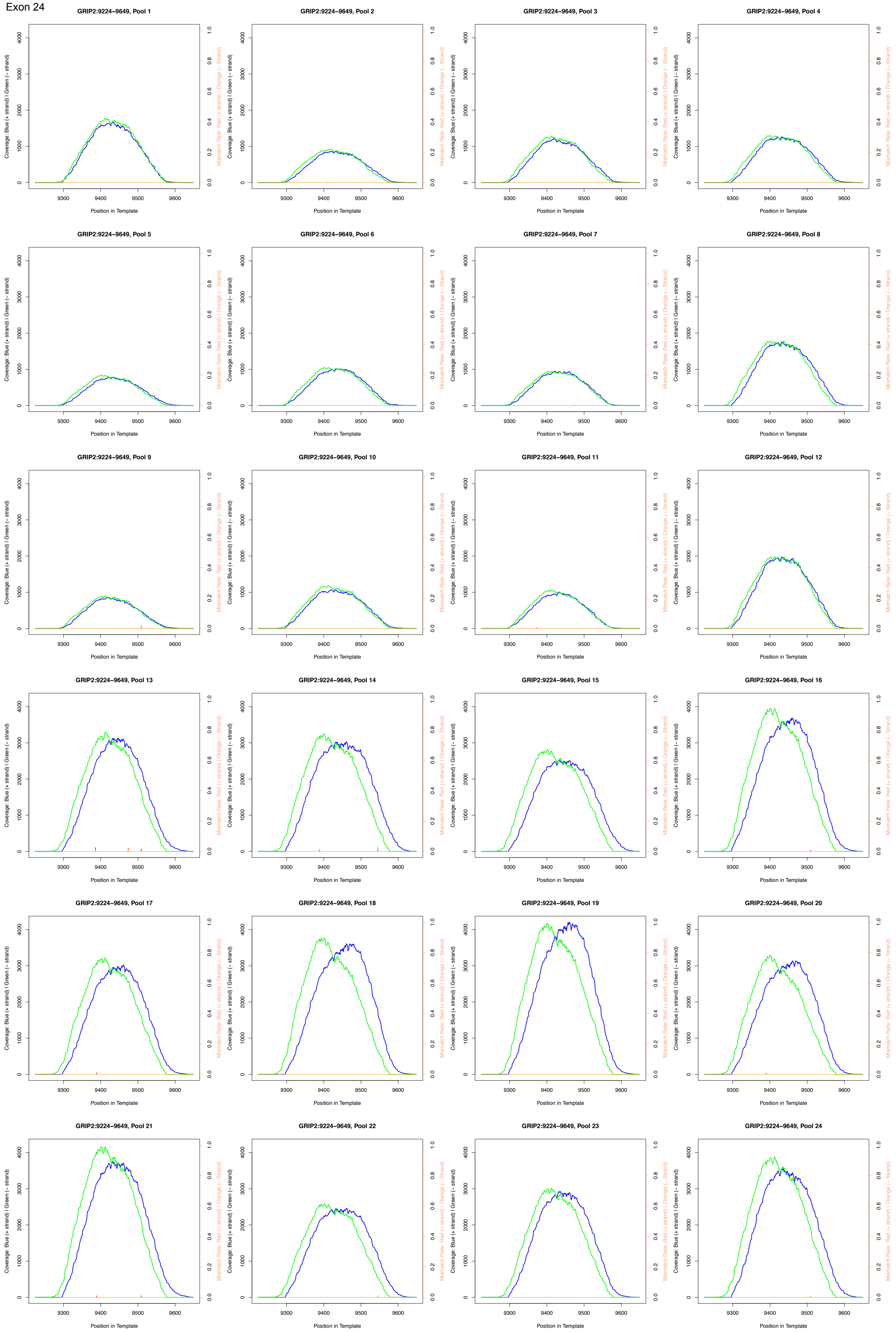
GRIP2:9224–9649, Pool 1
GRIP2:9224–9649, Pool 2
GRIP2:9224–9649, Pool 3
GRIP2:9224–9649, Pool 4
GRIP2:9224–9649, Pool 5
GRIP2:9224–9649, Pool 6
GRIP2:9224–9649, Pool 7
GRIP2:9224–9649, Pool 8
GRIP2:9224–9649, Pool 9
GRIP2:9224–9649, Pool 10
GRIP2:9224–9649, Pool 11
GRIP2:9224–9649, Pool 12
GRIP2:9224–9649, Pool 13
GRIP2:9224–9649, Pool 14
GRIP2:9224–9649, Pool 15
GRIP2:9224–9649, Pool 16
GRIP2:9224–9649, Pool 17
GRIP2:9224–9649, Pool 18
GRIP2:9224–9649, Pool 19
GRIP2:9224–9649, Pool 20
GRIP2:9224–9649, Pool 21
GRIP2:9224–9649, Pool 22
GRIP2:9224–9649, Pool 23
GRIP2:9224–9649, Pool 24
Coverage: Blue (+ strand) | Green (− strand)
Mismatch Rate: Red (+ strand) | Orange (− Strand)
Position in Template

Exon 23

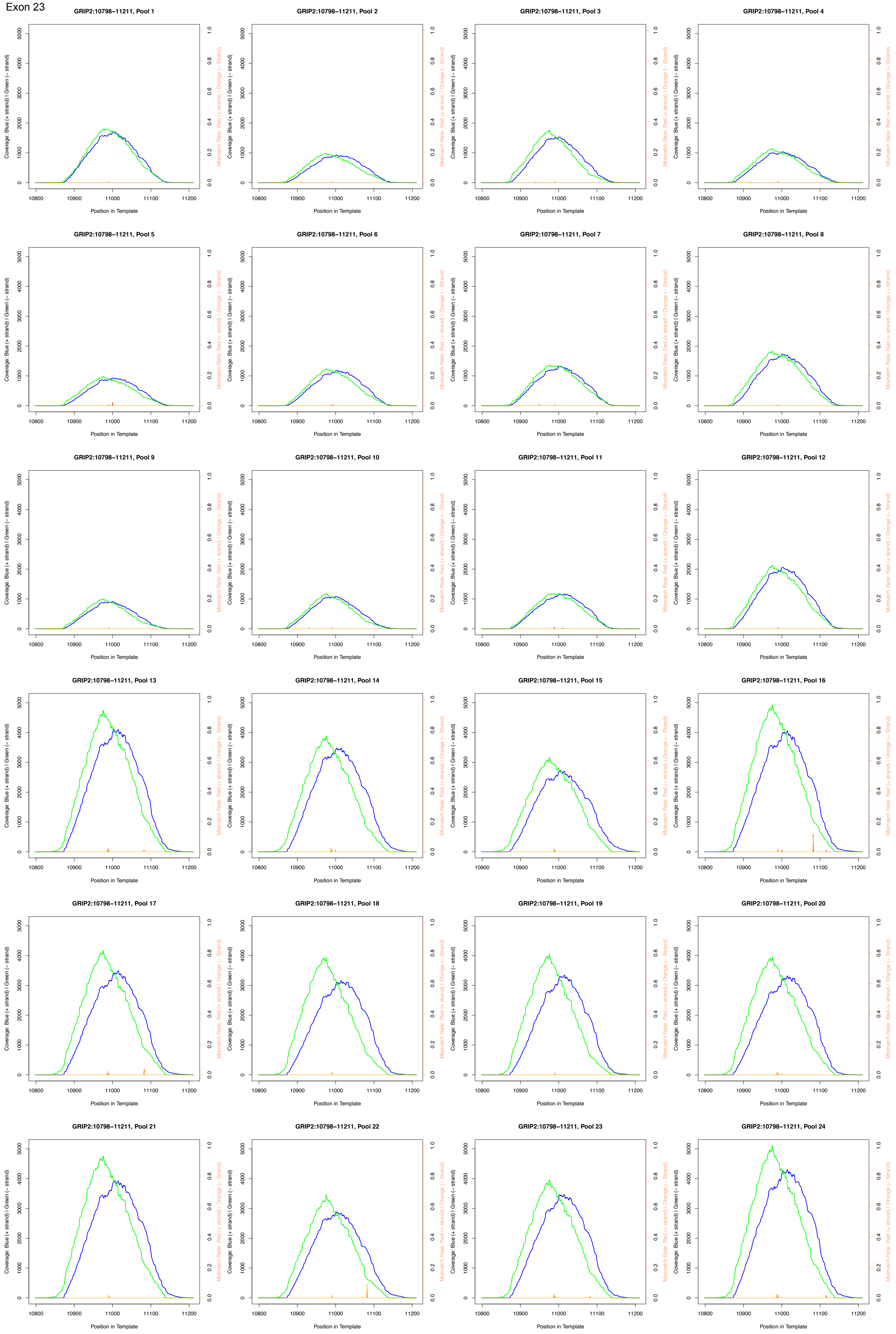
GRIP2:10798–11211, Pool 1
GRIP2:10798–11211, Pool 2
GRIP2:10798–11211, Pool 3
GRIP2:10798–11211, Pool 4
GRIP2:10798–11211, Pool 5
GRIP2:10798–11211, Pool 6
GRIP2:10798–11211, Pool 7
GRIP2:10798–11211, Pool 8
GRIP2:10798–11211, Pool 9
GRIP2:10798–11211, Pool 10
GRIP2:10798–11211, Pool 11
GRIP2:10798–11211, Pool 12
GRIP2:10798–11211, Pool 13
GRIP2:10798–11211, Pool 14
GRIP2:10798–11211, Pool 15
GRIP2:10798–11211, Pool 16
GRIP2:10798–11211, Pool 17
GRIP2:10798–11211, Pool 18
GRIP2:10798–11211, Pool 19
GRIP2:10798–11211, Pool 20
GRIP2:10798–11211, Pool 21
GRIP2:10798–11211, Pool 22
GRIP2:10798–11211, Pool 23
GRIP2:10798–11211, Pool 24
Coverage: Blue (+ strand) | Green (− strand)
Mismatch Rate: Red (+ strand) | Orange (− Strand)
Position in Template

Exon 21

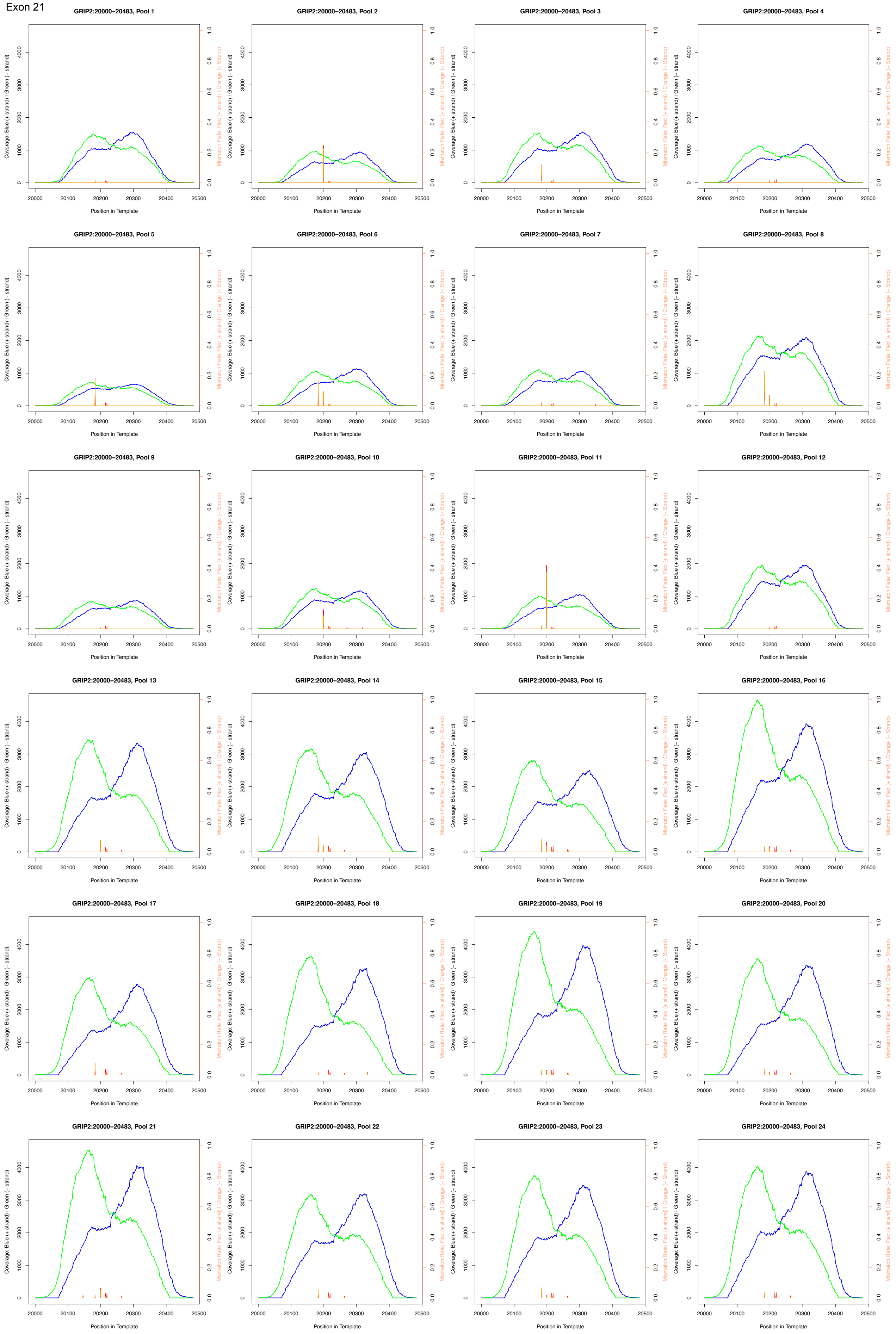
GRIP2:20000–20483, Pool 1
GRIP2:20000–20483, Pool 2
GRIP2:20000–20483, Pool 3
GRIP2:20000–20483, Pool 4
GRIP2:20000–20483, Pool 5
GRIP2:20000–20483, Pool 6
GRIP2:20000–20483, Pool 7
GRIP2:20000–20483, Pool 8
GRIP2:20000–20483, Pool 9
GRIP2:20000–20483, Pool 10
GRIP2:20000–20483, Pool 11
GRIP2:20000–20483, Pool 12
GRIP2:20000–20483, Pool 13
GRIP2:20000–20483, Pool 14
GRIP2:20000–20483, Pool 15
GRIP2:20000–20483, Pool 16
GRIP2:20000–20483, Pool 17
GRIP2:20000–20483, Pool 18
GRIP2:20000–20483, Pool 19
GRIP2:20000–20483, Pool 20
GRIP2:20000–20483, Pool 21
GRIP2:20000–20483, Pool 22
GRIP2:20000–20483, Pool 23
GRIP2:20000–20483, Pool 24
Coverage: Blue (+ strand) | Green (− strand)
Mismatch Rate: Red (+ strand) | Orange (− Strand)
Position in Template
20000
20100
20200
20300
20400
20500
0
1000
2000
3000
4000
0.0
0.2
0.4
0.6
0.8
1.0

Exon 20

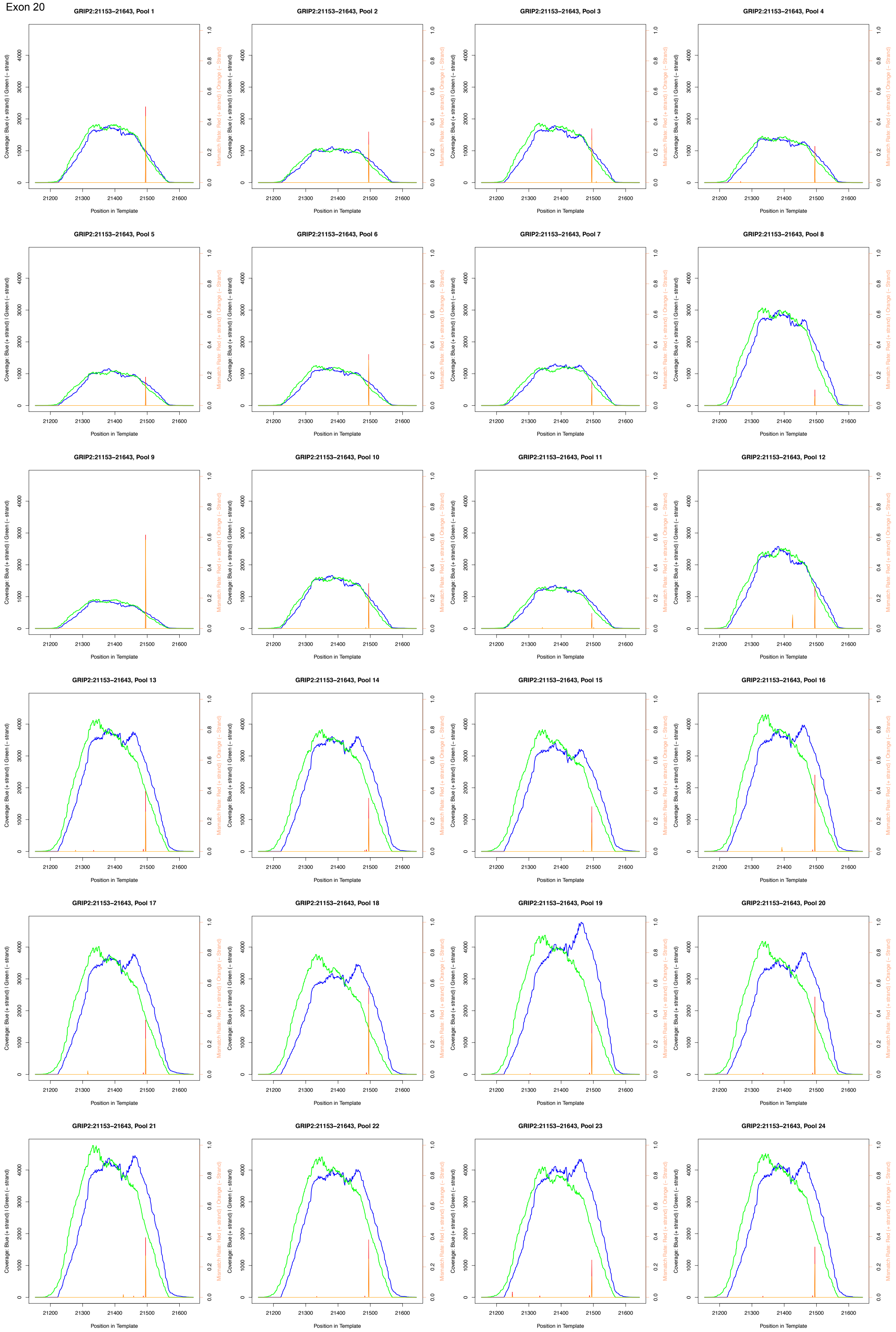
GRIP2:21153–21643, Pool 1
GRIP2:21153–21643, Pool 2
GRIP2:21153–21643, Pool 3
GRIP2:21153–21643, Pool 4
GRIP2:21153–21643, Pool 5
GRIP2:21153–21643, Pool 6
GRIP2:21153–21643, Pool 7
GRIP2:21153–21643, Pool 8
GRIP2:21153–21643, Pool 9
GRIP2:21153–21643, Pool 10
GRIP2:21153–21643, Pool 11
GRIP2:21153–21643, Pool 12
GRIP2:21153–21643, Pool 13
GRIP2:21153–21643, Pool 14
GRIP2:21153–21643, Pool 15
GRIP2:21153–21643, Pool 16
GRIP2:21153–21643, Pool 17
GRIP2:21153–21643, Pool 18
GRIP2:21153–21643, Pool 19
GRIP2:21153–21643, Pool 20
GRIP2:21153–21643, Pool 21
GRIP2:21153–21643, Pool 22
GRIP2:21153–21643, Pool 23
GRIP2:21153–21643, Pool 24
Coverage: Blue (+ strand) | Green (− strand)
Mismatch Rate: Red (+ strand) | Orange (− Strand)
Position in Template
21200
21300
21400
21500
21600
0
1000
2000
3000
4000
0.0
0.2
0.4
0.6
0.8
1.0

Exon 19

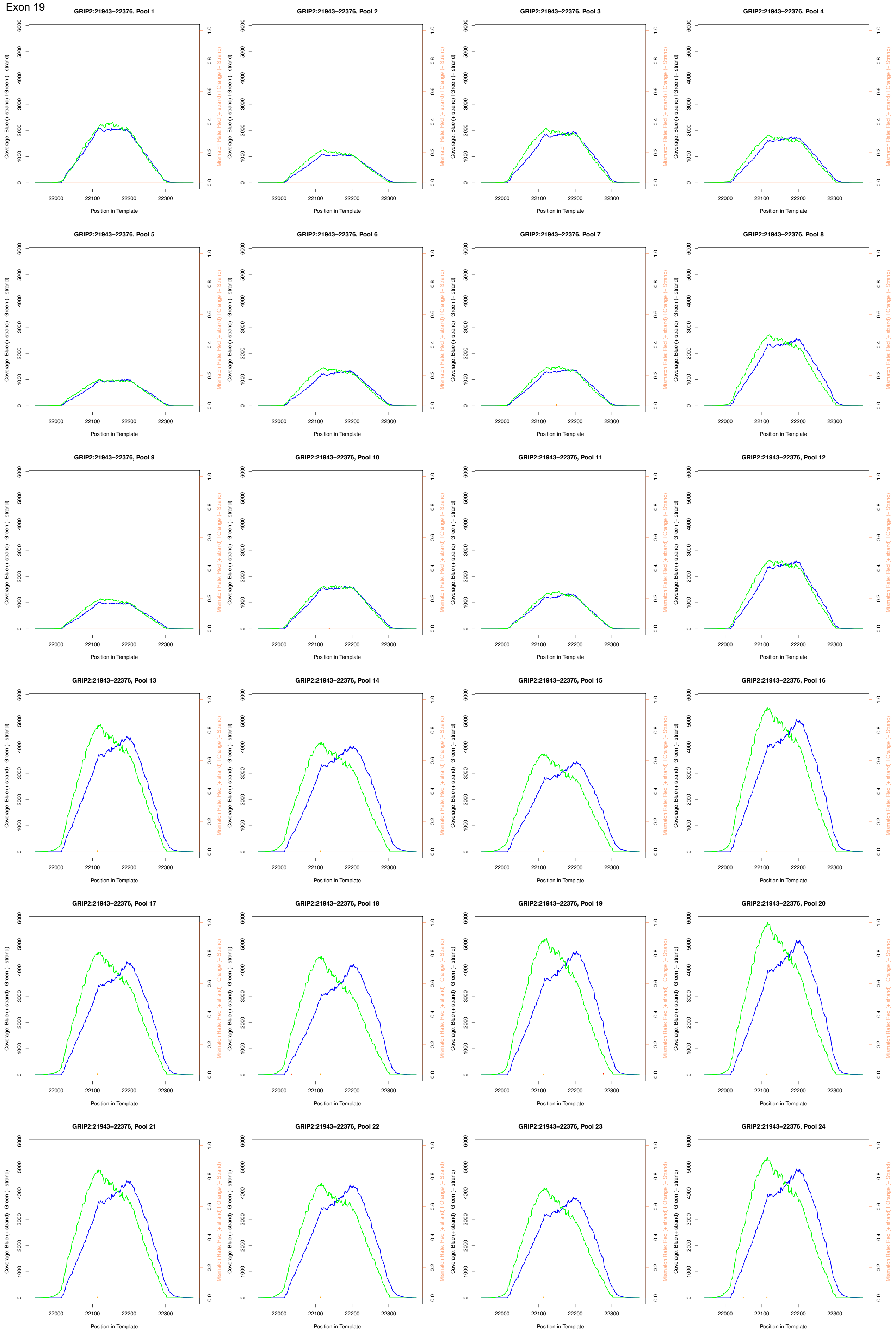
GRIP2:21943–22376, Pool 1
GRIP2:21943–22376, Pool 2
GRIP2:21943–22376, Pool 3
GRIP2:21943–22376, Pool 4
GRIP2:21943–22376, Pool 5
GRIP2:21943–22376, Pool 6
GRIP2:21943–22376, Pool 7
GRIP2:21943–22376, Pool 8
GRIP2:21943–22376, Pool 9
GRIP2:21943–22376, Pool 10
GRIP2:21943–22376, Pool 11
GRIP2:21943–22376, Pool 12
GRIP2:21943–22376, Pool 13
GRIP2:21943–22376, Pool 14
GRIP2:21943–22376, Pool 15
GRIP2:21943–22376, Pool 16
GRIP2:21943–22376, Pool 17
GRIP2:21943–22376, Pool 18
GRIP2:21943–22376, Pool 19
GRIP2:21943–22376, Pool 20
GRIP2:21943–22376, Pool 21
GRIP2:21943–22376, Pool 22
GRIP2:21943–22376, Pool 23
GRIP2:21943–22376, Pool 24
Coverage: Blue (+ strand) | Green (− strand)
Mismatch Rate: Red (+ strand) | Orange (− Strand)
Position in Template

Exon 18

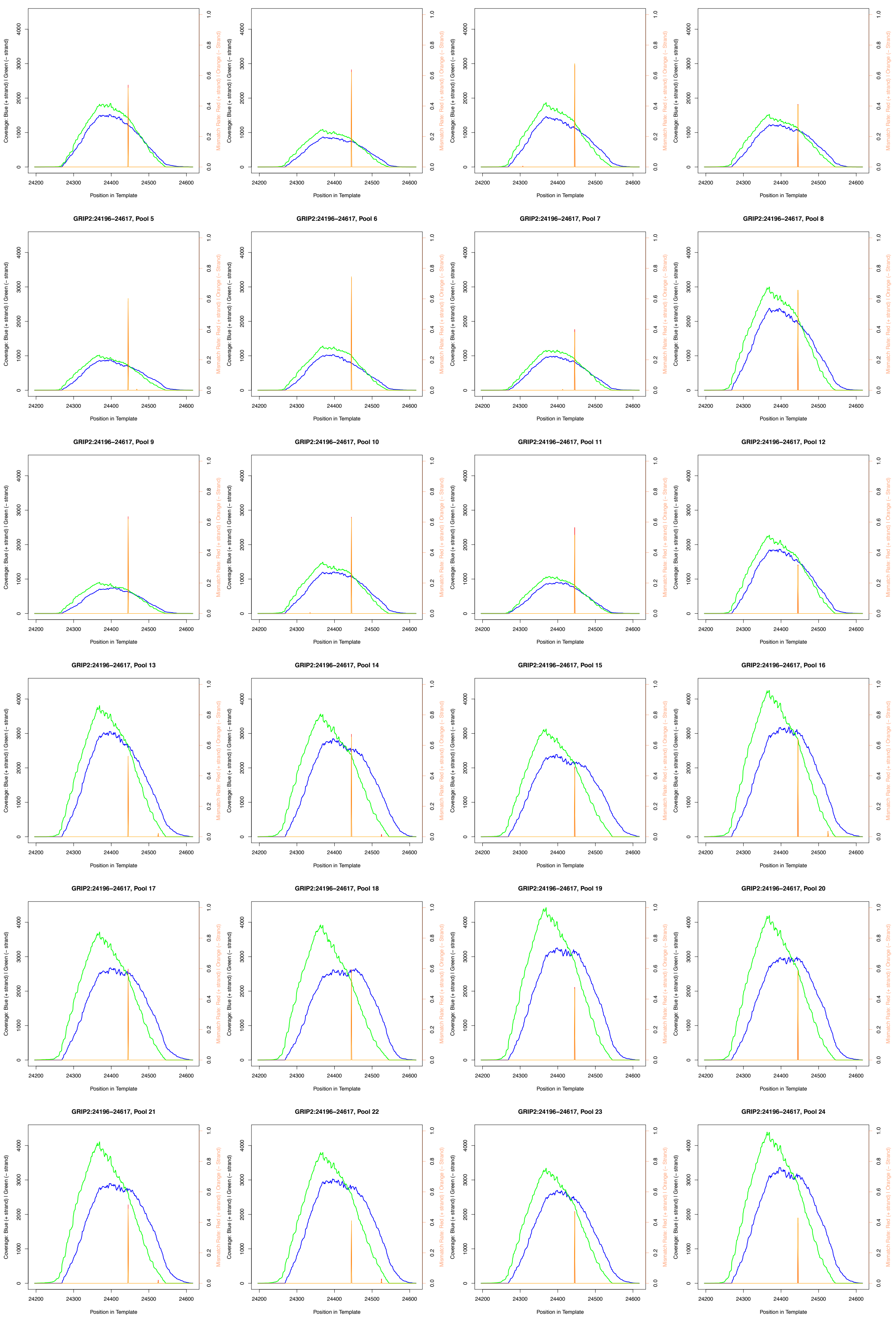

GRIP2:24196–24617, Pool 1
GRIP2:24196–24617, Pool 2
GRIP2:24196–24617, Pool 3
GRIP2:24196–24617, Pool 4
GRIP2:24196–24617, Pool 5
GRIP2:24196–24617, Pool 6
GRIP2:24196–24617, Pool 7
GRIP2:24196–24617, Pool 8
GRIP2:24196–24617, Pool 9
GRIP2:24196–24617, Pool 10
GRIP2:24196–24617, Pool 11
GRIP2:24196–24617, Pool 12
GRIP2:24196–24617, Pool 13
GRIP2:24196–24617, Pool 14
GRIP2:24196–24617, Pool 15
GRIP2:24196–24617, Pool 16
GRIP2:24196–24617, Pool 17
GRIP2:24196–24617, Pool 18
GRIP2:24196–24617, Pool 19
GRIP2:24196–24617, Pool 20
GRIP2:24196–24617, Pool 21
GRIP2:24196–24617, Pool 22
GRIP2:24196–24617, Pool 23
GRIP2:24196–24617, Pool 24
Coverage: Blue (+ strand) | Green (− strand)
Mismatch Rate: Red (+ strand) | Orange (− Strand)
Position in Template

Exons 16 & 17

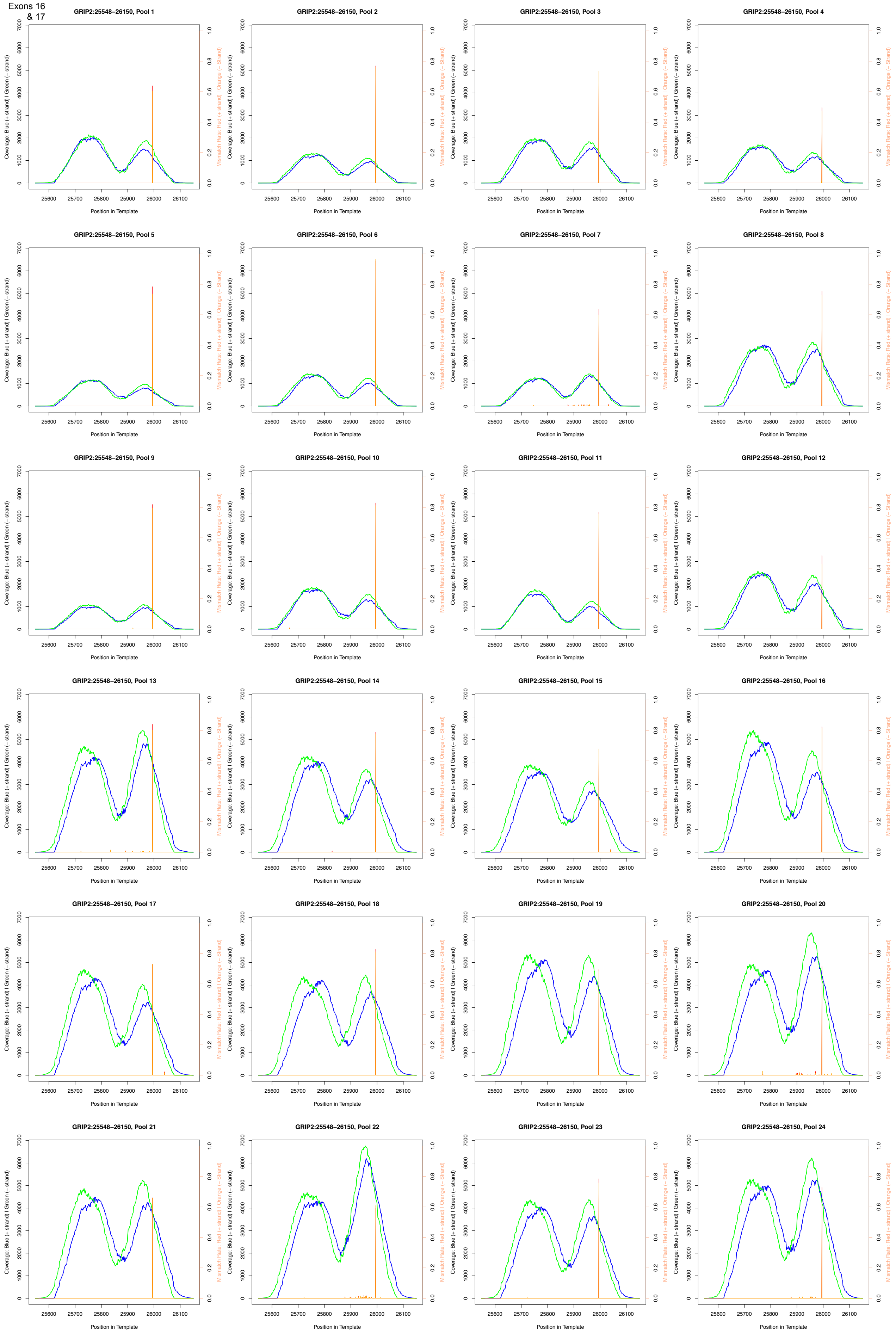
GRIP2:25548–26150, Pool 1
GRIP2:25548–26150, Pool 2
GRIP2:25548–26150, Pool 3
GRIP2:25548–26150, Pool 4
GRIP2:25548–26150, Pool 5
GRIP2:25548–26150, Pool 6
GRIP2:25548–26150, Pool 7
GRIP2:25548–26150, Pool 8
GRIP2:25548–26150, Pool 9
GRIP2:25548–26150, Pool 10
GRIP2:25548–26150, Pool 11
GRIP2:25548–26150, Pool 12
GRIP2:25548–26150, Pool 13
GRIP2:25548–26150, Pool 14
GRIP2:25548–26150, Pool 15
GRIP2:25548–26150, Pool 16
GRIP2:25548–26150, Pool 17
GRIP2:25548–26150, Pool 18
GRIP2:25548–26150, Pool 19
GRIP2:25548–26150, Pool 20
GRIP2:25548–26150, Pool 21
GRIP2:25548–26150, Pool 22
GRIP2:25548–26150, Pool 23
GRIP2:25548–26150, Pool 24
Coverage: Blue (+ strand) | Green (– strand)
Mismatch Rate: Red (+ strand) | Orange (– Strand)
Position in Template
0
1000
2000
3000
4000
5000
6000
7000
0.0
0.2
0.4
0.6
0.8
1.0
25600
25700
25800
25900
26000
26100

Exon 15

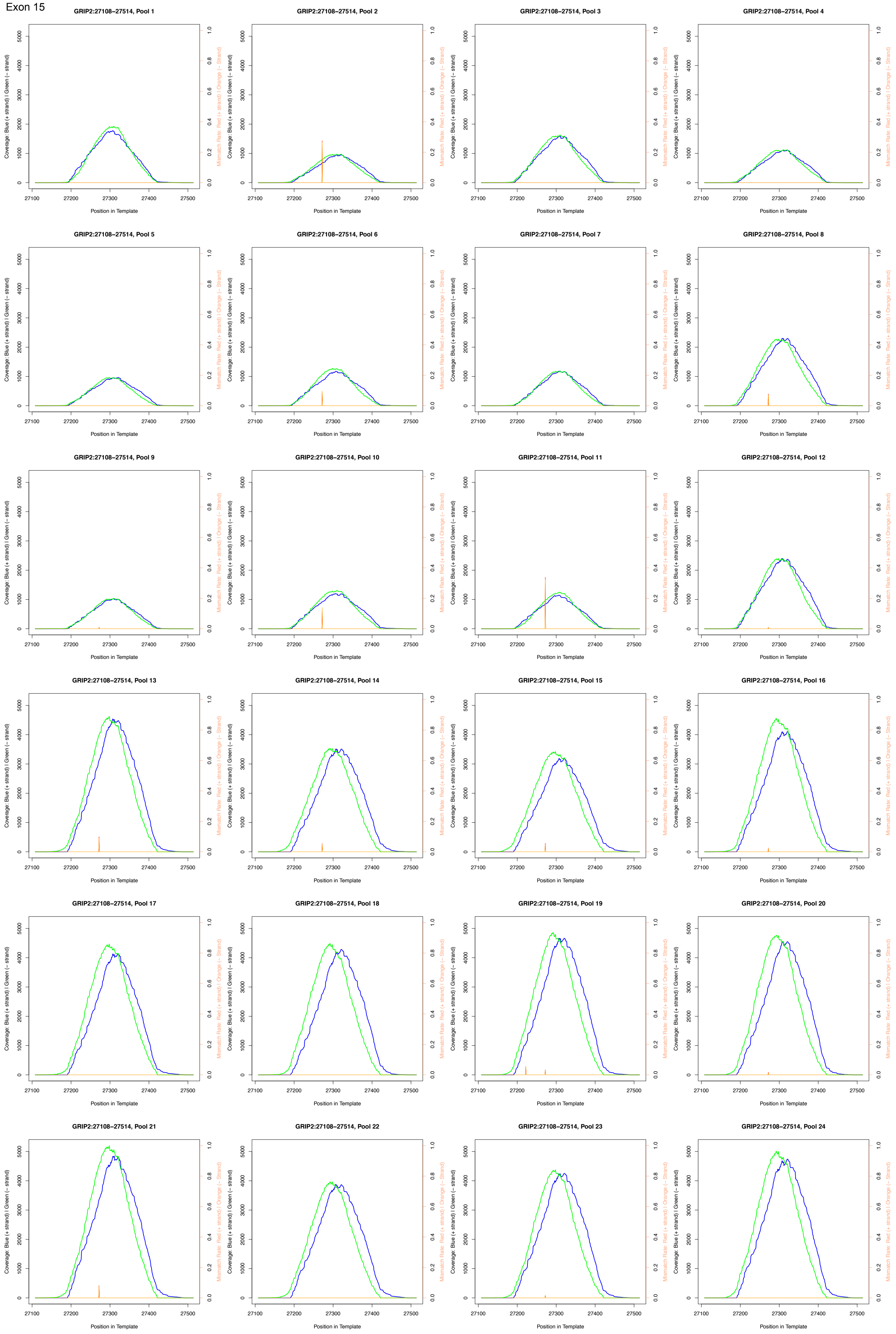
GRIP2:27108–27514, Pool 1
GRIP2:27108–27514, Pool 2
GRIP2:27108–27514, Pool 3
GRIP2:27108–27514, Pool 4
GRIP2:27108–27514, Pool 5
GRIP2:27108–27514, Pool 6
GRIP2:27108–27514, Pool 7
GRIP2:27108–27514, Pool 8
GRIP2:27108–27514, Pool 9
GRIP2:27108–27514, Pool 10
GRIP2:27108–27514, Pool 11
GRIP2:27108–27514, Pool 12
GRIP2:27108–27514, Pool 13
GRIP2:27108–27514, Pool 14
GRIP2:27108–27514, Pool 15
GRIP2:27108–27514, Pool 16
GRIP2:27108–27514, Pool 17
GRIP2:27108–27514, Pool 18
GRIP2:27108–27514, Pool 19
GRIP2:27108–27514, Pool 20
GRIP2:27108–27514, Pool 21
GRIP2:27108–27514, Pool 22
GRIP2:27108–27514, Pool 23
GRIP2:27108–27514, Pool 24
Coverage: Blue (+ strand) | Green (– strand)
Mismatch Rate: Red (+ strand) | Orange (– Strand)
Position in Template

Exon 14

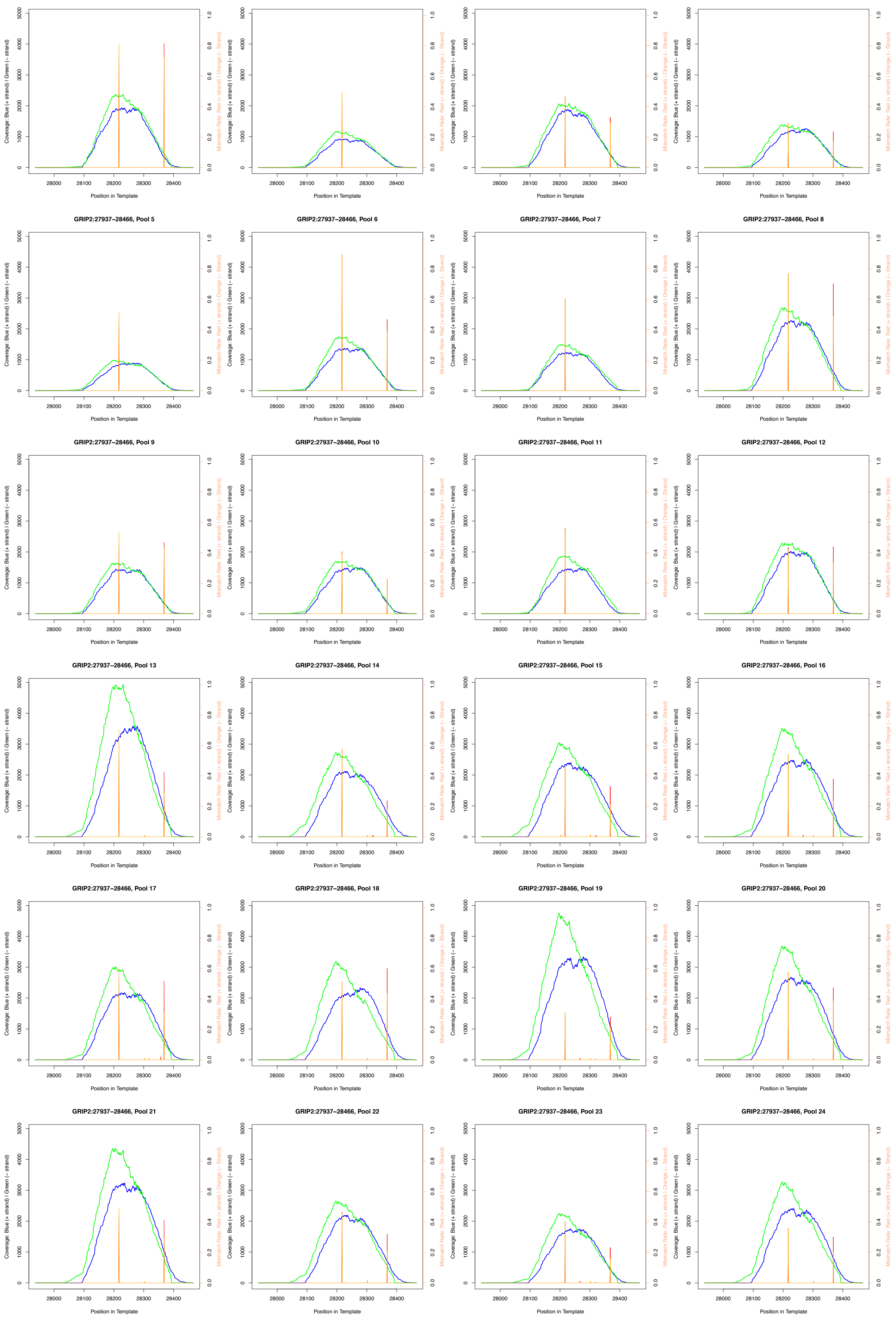

GRIP2:27937–28466, Pool 1
GRIP2:27937–28466, Pool 2
GRIP2:27937–28466, Pool 3
GRIP2:27937–28466, Pool 4
GRIP2:27937–28466, Pool 5
GRIP2:27937–28466, Pool 6
GRIP2:27937–28466, Pool 7
GRIP2:27937–28466, Pool 8
GRIP2:27937–28466, Pool 9
GRIP2:27937–28466, Pool 10
GRIP2:27937–28466, Pool 11
GRIP2:27937–28466, Pool 12
GRIP2:27937–28466, Pool 13
GRIP2:27937–28466, Pool 14
GRIP2:27937–28466, Pool 15
GRIP2:27937–28466, Pool 16
GRIP2:27937–28466, Pool 17
GRIP2:27937–28466, Pool 18
GRIP2:27937–28466, Pool 19
GRIP2:27937–28466, Pool 20
GRIP2:27937–28466, Pool 21
GRIP2:27937–28466, Pool 22
GRIP2:27937–28466, Pool 23
GRIP2:27937–28466, Pool 24
Coverage: Blue (+ strand) | Green (– strand)
Mismatch Rate: Red (+ strand) | Orange (– Strand)
Position in Template

Exon 13

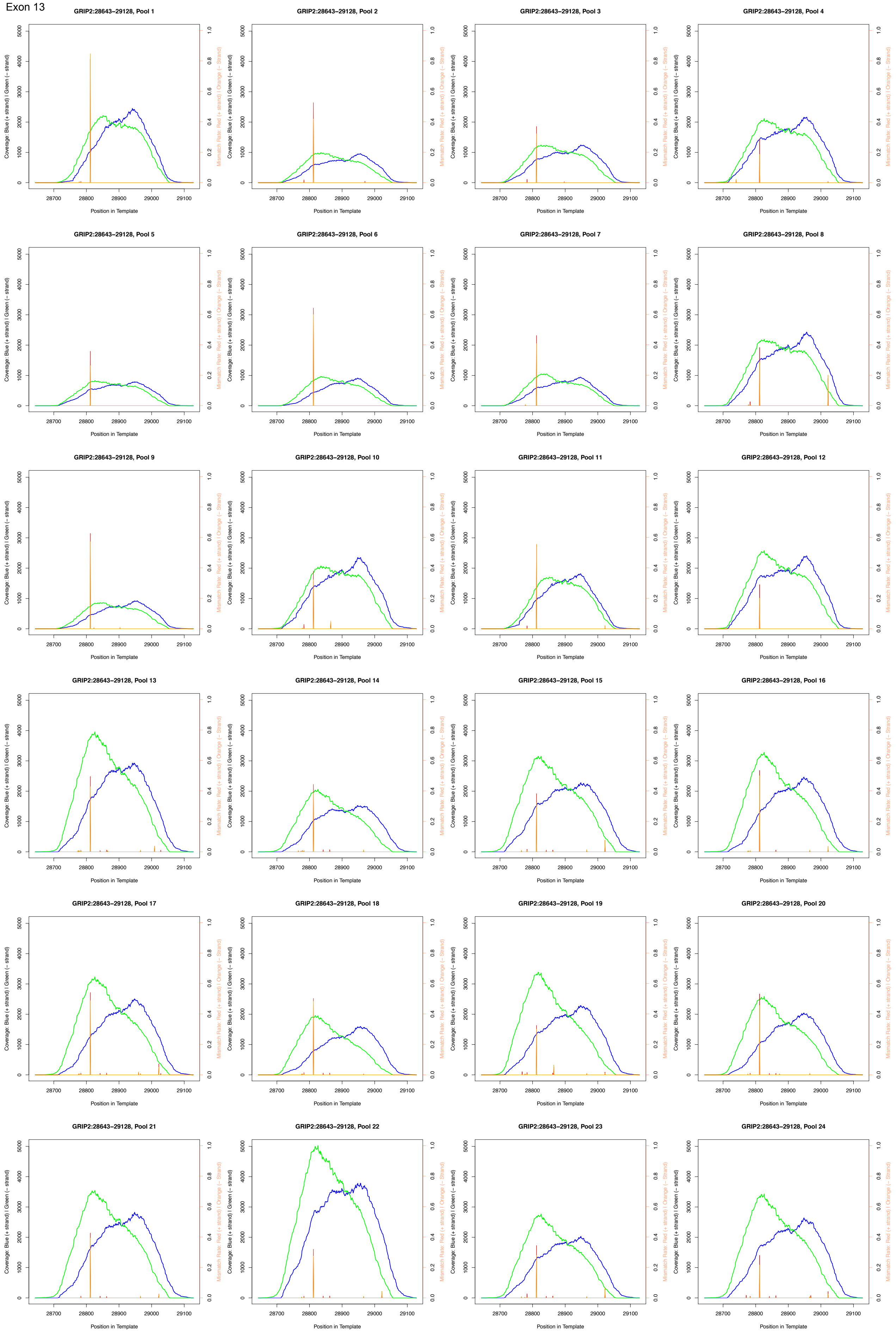
GRIP2:28643–29128, Pool 1
GRIP2:28643–29128, Pool 2
GRIP2:28643–29128, Pool 3
GRIP2:28643–29128, Pool 4
GRIP2:28643–29128, Pool 5
GRIP2:28643–29128, Pool 6
GRIP2:28643–29128, Pool 7
GRIP2:28643–29128, Pool 8
GRIP2:28643–29128, Pool 9
GRIP2:28643–29128, Pool 10
GRIP2:28643–29128, Pool 11
GRIP2:28643–29128, Pool 12
GRIP2:28643–29128, Pool 13
GRIP2:28643–29128, Pool 14
GRIP2:28643–29128, Pool 15
GRIP2:28643–29128, Pool 16
GRIP2:28643–29128, Pool 17
GRIP2:28643–29128, Pool 18
GRIP2:28643–29128, Pool 19
GRIP2:28643–29128, Pool 20
GRIP2:28643–29128, Pool 21
GRIP2:28643–29128, Pool 22
GRIP2:28643–29128, Pool 23
GRIP2:28643–29128, Pool 24
Coverage: Blue (+ strand) | Green (− strand)
Mismatch Rate: Red (+ strand) | Orange (− Strand)
Position in Template

Exon 12

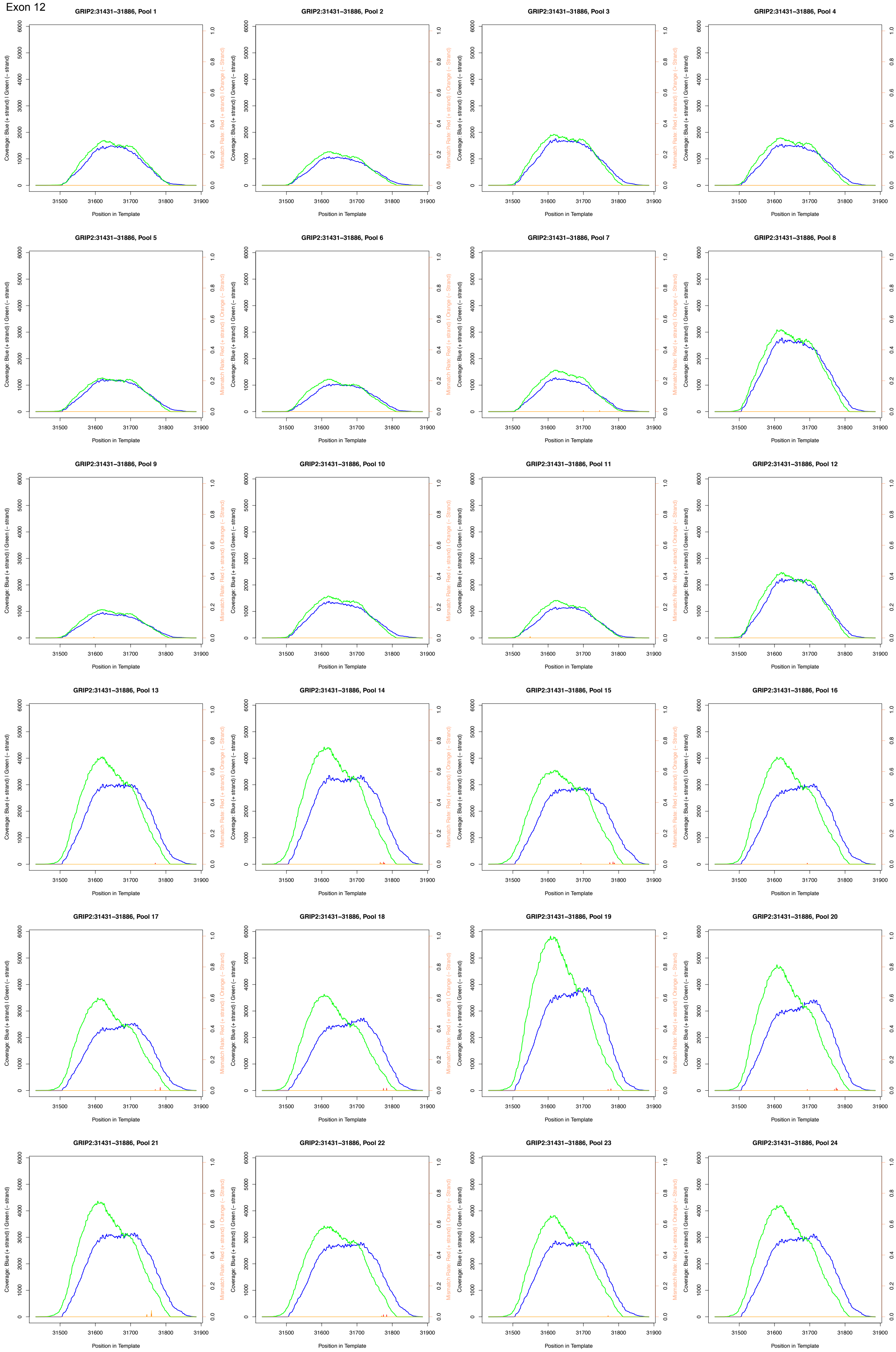

GRIP2:31431–31886, Pool 1
GRIP2:31431–31886, Pool 2
GRIP2:31431–31886, Pool 3
GRIP2:31431–31886, Pool 4
GRIP2:31431–31886, Pool 5
GRIP2:31431–31886, Pool 6
GRIP2:31431–31886, Pool 7
GRIP2:31431–31886, Pool 8
GRIP2:31431–31886, Pool 9
GRIP2:31431–31886, Pool 10
GRIP2:31431–31886, Pool 11
GRIP2:31431–31886, Pool 12
GRIP2:31431–31886, Pool 13
GRIP2:31431–31886, Pool 14
GRIP2:31431–31886, Pool 15
GRIP2:31431–31886, Pool 16
GRIP2:31431–31886, Pool 17
GRIP2:31431–31886, Pool 18
GRIP2:31431–31886, Pool 19
GRIP2:31431–31886, Pool 20
GRIP2:31431–31886, Pool 21
GRIP2:31431–31886, Pool 22
GRIP2:31431–31886, Pool 23
GRIP2:31431–31886, Pool 24
Coverage: Blue (+ strand) | Green (− strand)
Mismatch Rate: Red (+ strand) | Orange (− Strand)
Position in Template

Exon 11

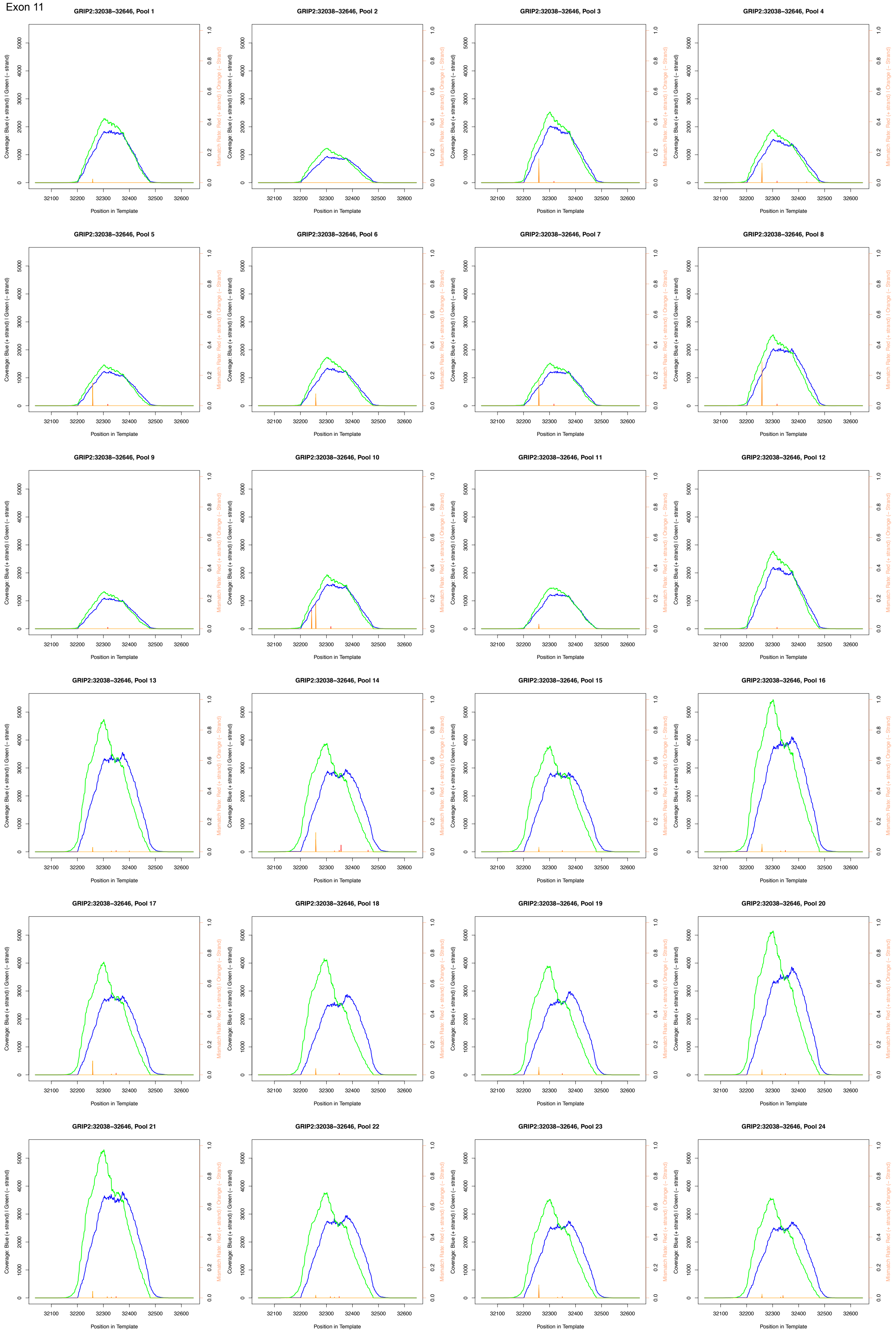
GRIP2:32038–32646, Pool 1
GRIP2:32038–32646, Pool 2
GRIP2:32038–32646, Pool 3
GRIP2:32038–32646, Pool 4
GRIP2:32038–32646, Pool 5
GRIP2:32038–32646, Pool 6
GRIP2:32038–32646, Pool 7
GRIP2:32038–32646, Pool 8
GRIP2:32038–32646, Pool 9
GRIP2:32038–32646, Pool 10
GRIP2:32038–32646, Pool 11
GRIP2:32038–32646, Pool 12
GRIP2:32038–32646, Pool 13
GRIP2:32038–32646, Pool 14
GRIP2:32038–32646, Pool 15
GRIP2:32038–32646, Pool 16
GRIP2:32038–32646, Pool 17
GRIP2:32038–32646, Pool 18
GRIP2:32038–32646, Pool 19
GRIP2:32038–32646, Pool 20
GRIP2:32038–32646, Pool 21
GRIP2:32038–32646, Pool 22
GRIP2:32038–32646, Pool 23
GRIP2:32038–32646, Pool 24
Coverage: Blue (+ strand) | Green (– strand)
Mismatch Rate: Red (+ strand) | Orange (– Strand)
Position in Template

Exon 9 & 10

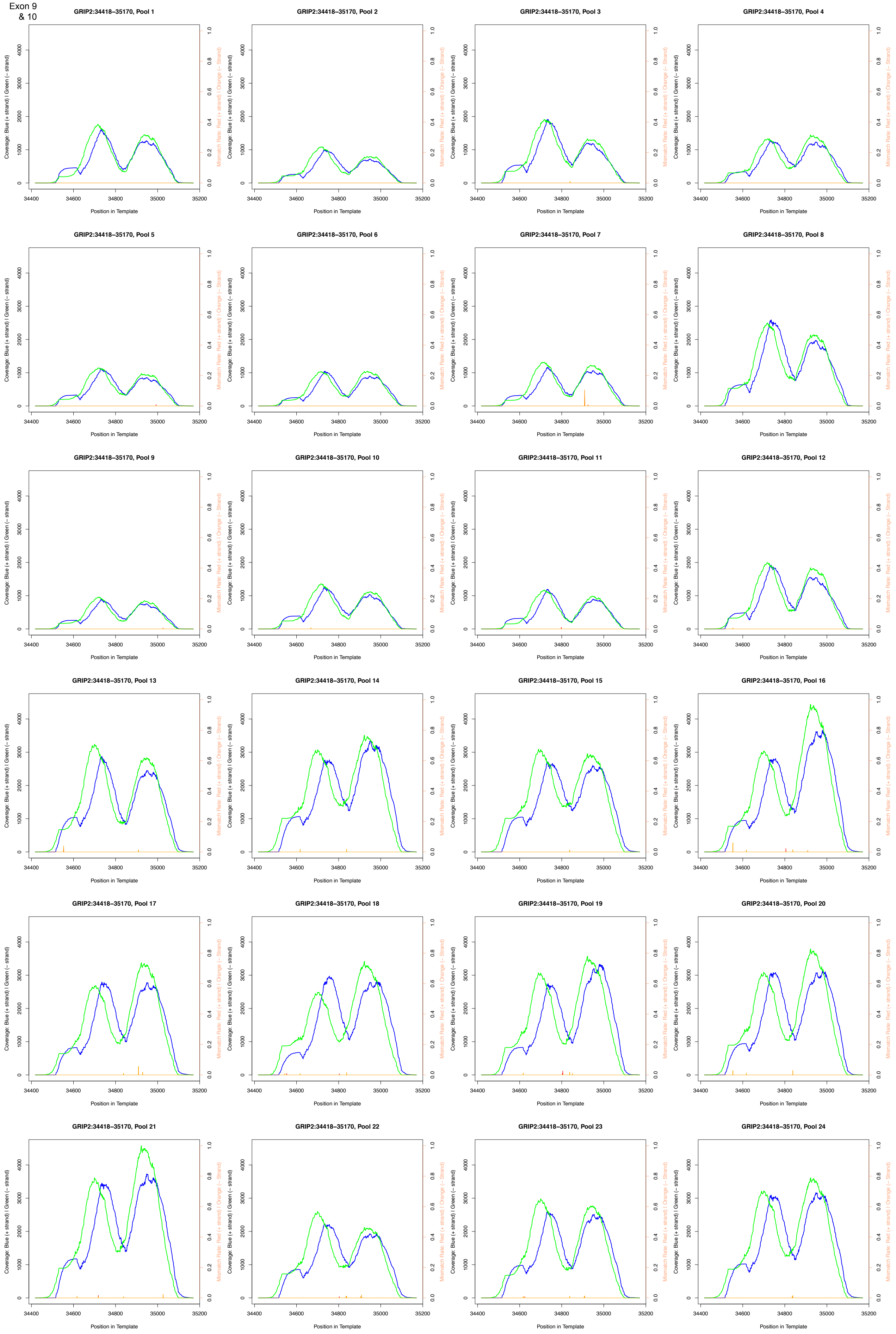
GRIP2:34418–35170, Pool 1
GRIP2:34418–35170, Pool 2
GRIP2:34418–35170, Pool 3
GRIP2:34418–35170, Pool 4
GRIP2:34418–35170, Pool 5
GRIP2:34418–35170, Pool 6
GRIP2:34418–35170, Pool 7
GRIP2:34418–35170, Pool 8
GRIP2:34418–35170, Pool 9
GRIP2:34418–35170, Pool 10
GRIP2:34418–35170, Pool 11
GRIP2:34418–35170, Pool 12
GRIP2:34418–35170, Pool 13
GRIP2:34418–35170, Pool 14
GRIP2:34418–35170, Pool 15
GRIP2:34418–35170, Pool 16
GRIP2:34418–35170, Pool 17
GRIP2:34418–35170, Pool 18
GRIP2:34418–35170, Pool 19
GRIP2:34418–35170, Pool 20
GRIP2:34418–35170, Pool 21
GRIP2:34418–35170, Pool 22
GRIP2:34418–35170, Pool 23
GRIP2:34418–35170, Pool 24
Coverage: Blue (+ strand) | Green (− strand)
Mismatch Rate: Red (+ strand) | Orange (− Strand)
Position in Template
34400
34600
34800
35000
35200
0
1000
2000
3000
4000
0.0
0.2
0.4
0.6
0.8
1.0

Exon 8

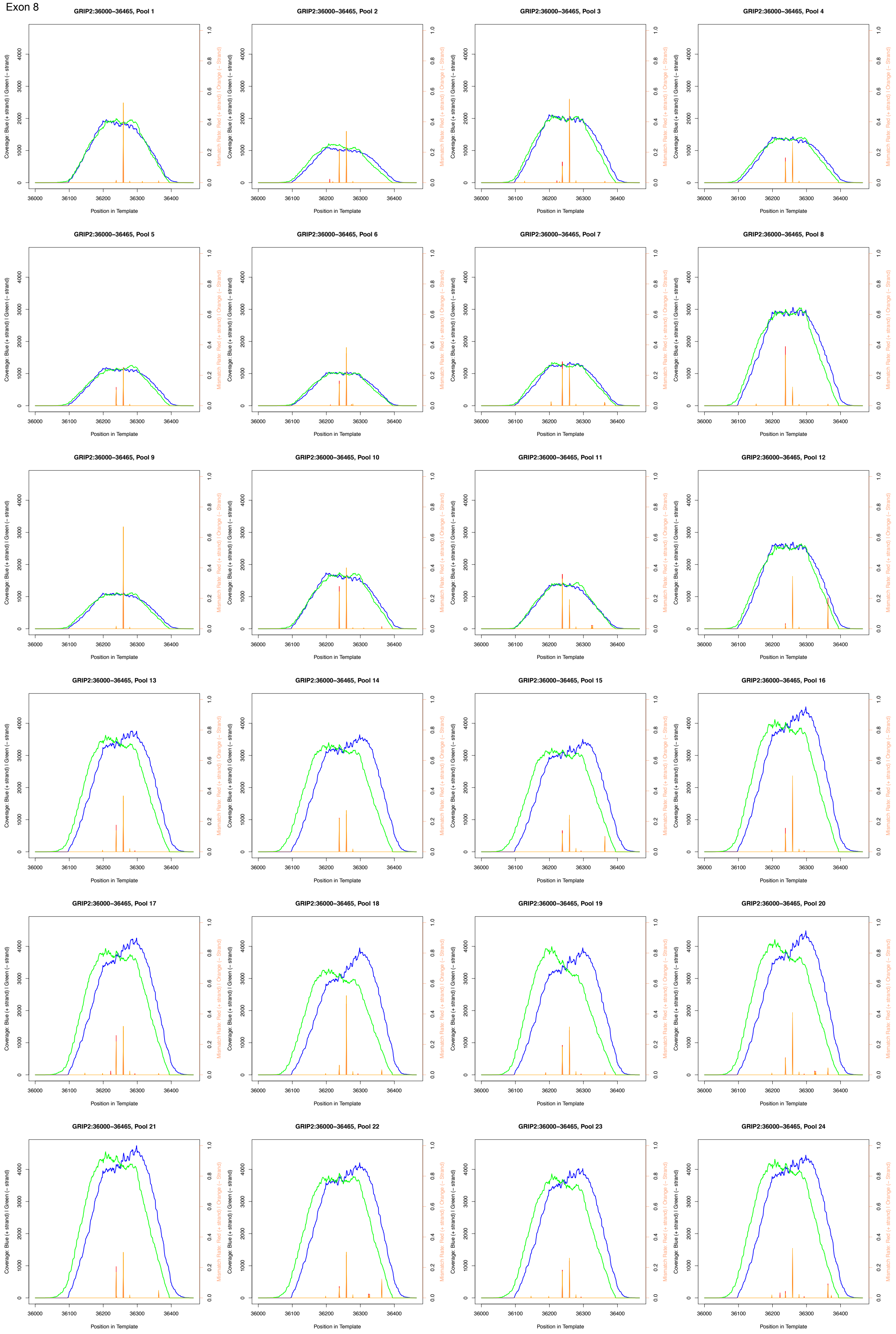
GRIP2:36000–36465, Pool 1
GRIP2:36000–36465, Pool 2
GRIP2:36000–36465, Pool 3
GRIP2:36000–36465, Pool 4
GRIP2:36000–36465, Pool 5
GRIP2:36000–36465, Pool 6
GRIP2:36000–36465, Pool 7
GRIP2:36000–36465, Pool 8
GRIP2:36000–36465, Pool 9
GRIP2:36000–36465, Pool 10
GRIP2:36000–36465, Pool 11
GRIP2:36000–36465, Pool 12
GRIP2:36000–36465, Pool 13
GRIP2:36000–36465, Pool 14
GRIP2:36000–36465, Pool 15
GRIP2:36000–36465, Pool 16
GRIP2:36000–36465, Pool 17
GRIP2:36000–36465, Pool 18
GRIP2:36000–36465, Pool 19
GRIP2:36000–36465, Pool 20
GRIP2:36000–36465, Pool 21
GRIP2:36000–36465, Pool 22
GRIP2:36000–36465, Pool 23
GRIP2:36000–36465, Pool 24
Coverage: Blue (+ strand) | Green (− strand)
Mismatch Rate: Red (+ strand) | Orange (− Strand)
Position in Template

Exon 7

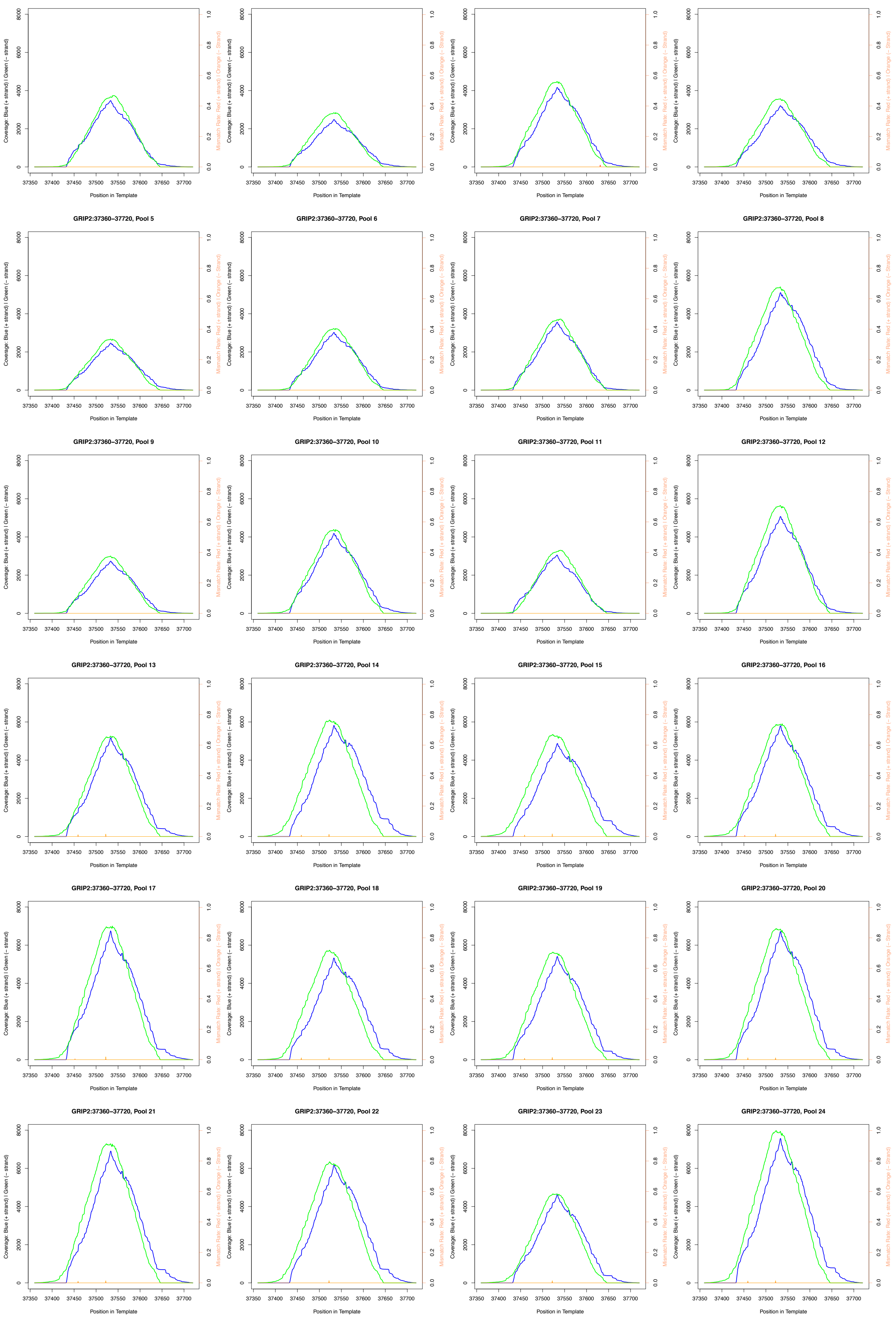
GRIP2:37360–37720, Pool 1
GRIP2:37360–37720, Pool 2
GRIP2:37360–37720, Pool 3
GRIP2:37360–37720, Pool 4
GRIP2:37360–37720, Pool 5
GRIP2:37360–37720, Pool 6
GRIP2:37360–37720, Pool 7
GRIP2:37360–37720, Pool 8
GRIP2:37360–37720, Pool 9
GRIP2:37360–37720, Pool 10
GRIP2:37360–37720, Pool 11
GRIP2:37360–37720, Pool 12
GRIP2:37360–37720, Pool 13
GRIP2:37360–37720, Pool 14
GRIP2:37360–37720, Pool 15
GRIP2:37360–37720, Pool 16
GRIP2:37360–37720, Pool 17
GRIP2:37360–37720, Pool 18
GRIP2:37360–37720, Pool 19
GRIP2:37360–37720, Pool 20
GRIP2:37360–37720, Pool 21
GRIP2:37360–37720, Pool 22
GRIP2:37360–37720, Pool 23
GRIP2:37360–37720, Pool 24
Coverage: Blue (+ strand) | Green (− strand)
Mismatch Rate: Red (+ strand) | Orange (− Strand)
Position in Template

Exon 6

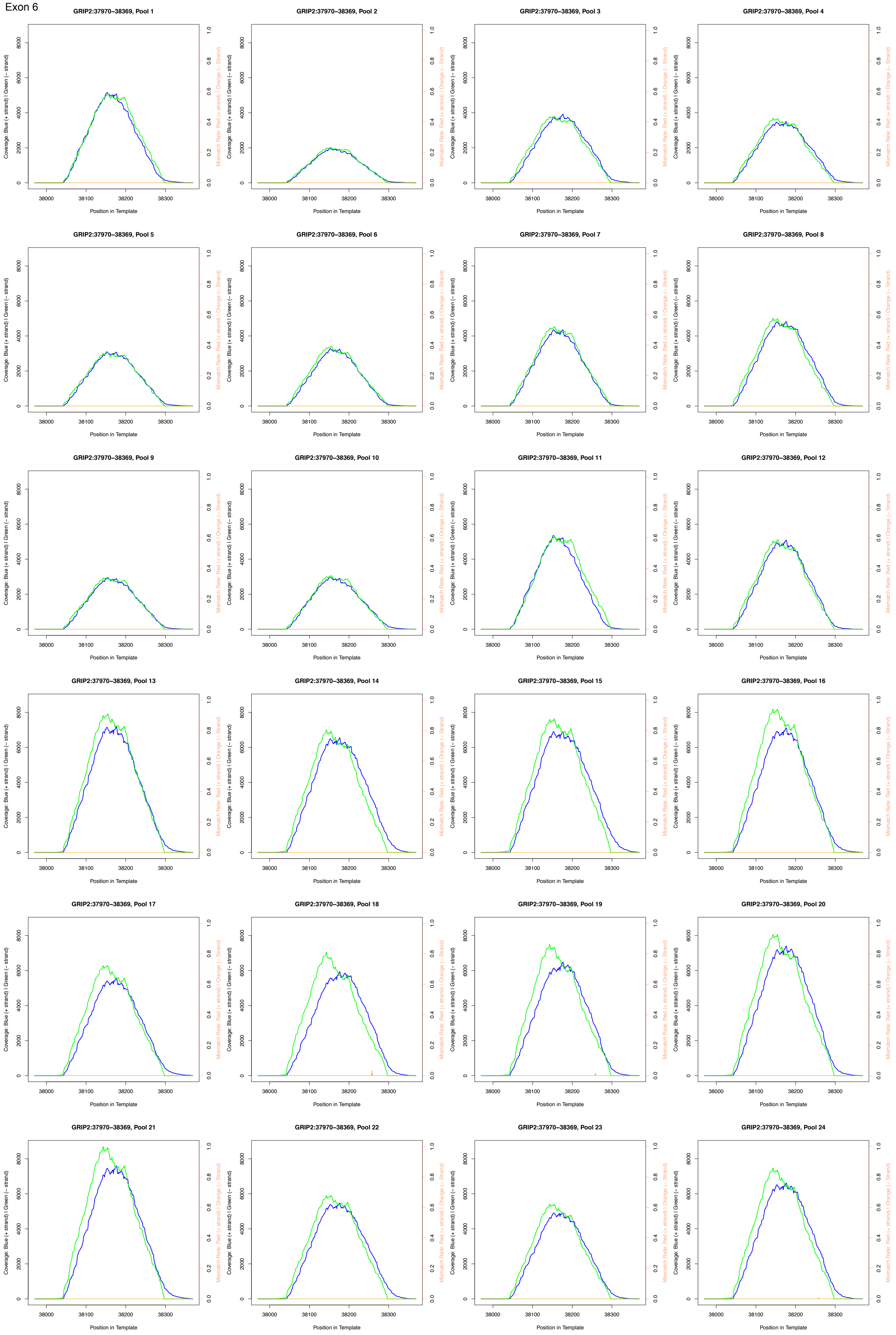

GRIP2:37970–38369, Pool 1
GRIP2:37970–38369, Pool 2
GRIP2:37970–38369, Pool 3
GRIP2:37970–38369, Pool 4
GRIP2:37970–38369, Pool 5
GRIP2:37970–38369, Pool 6
GRIP2:37970–38369, Pool 7
GRIP2:37970–38369, Pool 8
GRIP2:37970–38369, Pool 9
GRIP2:37970–38369, Pool 10
GRIP2:37970–38369, Pool 11
GRIP2:37970–38369, Pool 12
GRIP2:37970–38369, Pool 13
GRIP2:37970–38369, Pool 14
GRIP2:37970–38369, Pool 15
GRIP2:37970–38369, Pool 16
GRIP2:37970–38369, Pool 17
GRIP2:37970–38369, Pool 18
GRIP2:37970–38369, Pool 19
GRIP2:37970–38369, Pool 20
GRIP2:37970–38369, Pool 21
GRIP2:37970–38369, Pool 22
GRIP2:37970–38369, Pool 23
GRIP2:37970–38369, Pool 24
Coverage: Blue (+ strand) | Green (– strand)
Mismatch Rate: Red (+ strand) | Orange (– Strand)
Position in Template

Exon 5

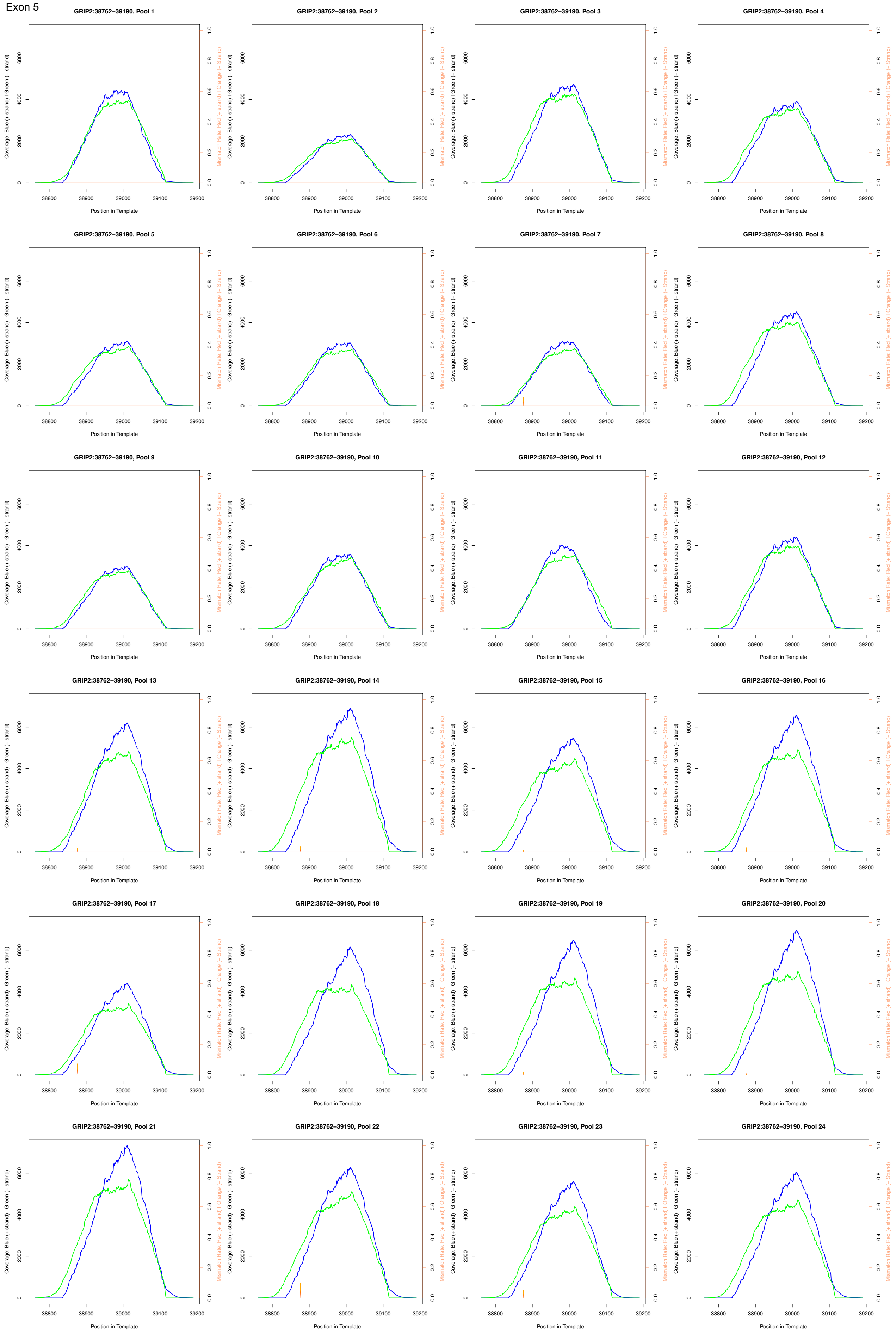

GRIP2:38762–39190, Pool 1
GRIP2:38762–39190, Pool 2
GRIP2:38762–39190, Pool 3
GRIP2:38762–39190, Pool 4
GRIP2:38762–39190, Pool 5
GRIP2:38762–39190, Pool 6
GRIP2:38762–39190, Pool 7
GRIP2:38762–39190, Pool 8
GRIP2:38762–39190, Pool 9
GRIP2:38762–39190, Pool 10
GRIP2:38762–39190, Pool 11
GRIP2:38762–39190, Pool 12
GRIP2:38762–39190, Pool 13
GRIP2:38762–39190, Pool 14
GRIP2:38762–39190, Pool 15
GRIP2:38762–39190, Pool 16
GRIP2:38762–39190, Pool 17
GRIP2:38762–39190, Pool 18
GRIP2:38762–39190, Pool 19
GRIP2:38762–39190, Pool 20
GRIP2:38762–39190, Pool 21
GRIP2:38762–39190, Pool 22
GRIP2:38762–39190, Pool 23
GRIP2:38762–39190, Pool 24
Coverage: Blue (+ strand) | Green (– strand)
Mismatch Rate: Red (+ strand) | Orange (– Strand)
Position in Template

Exon 3 & 4

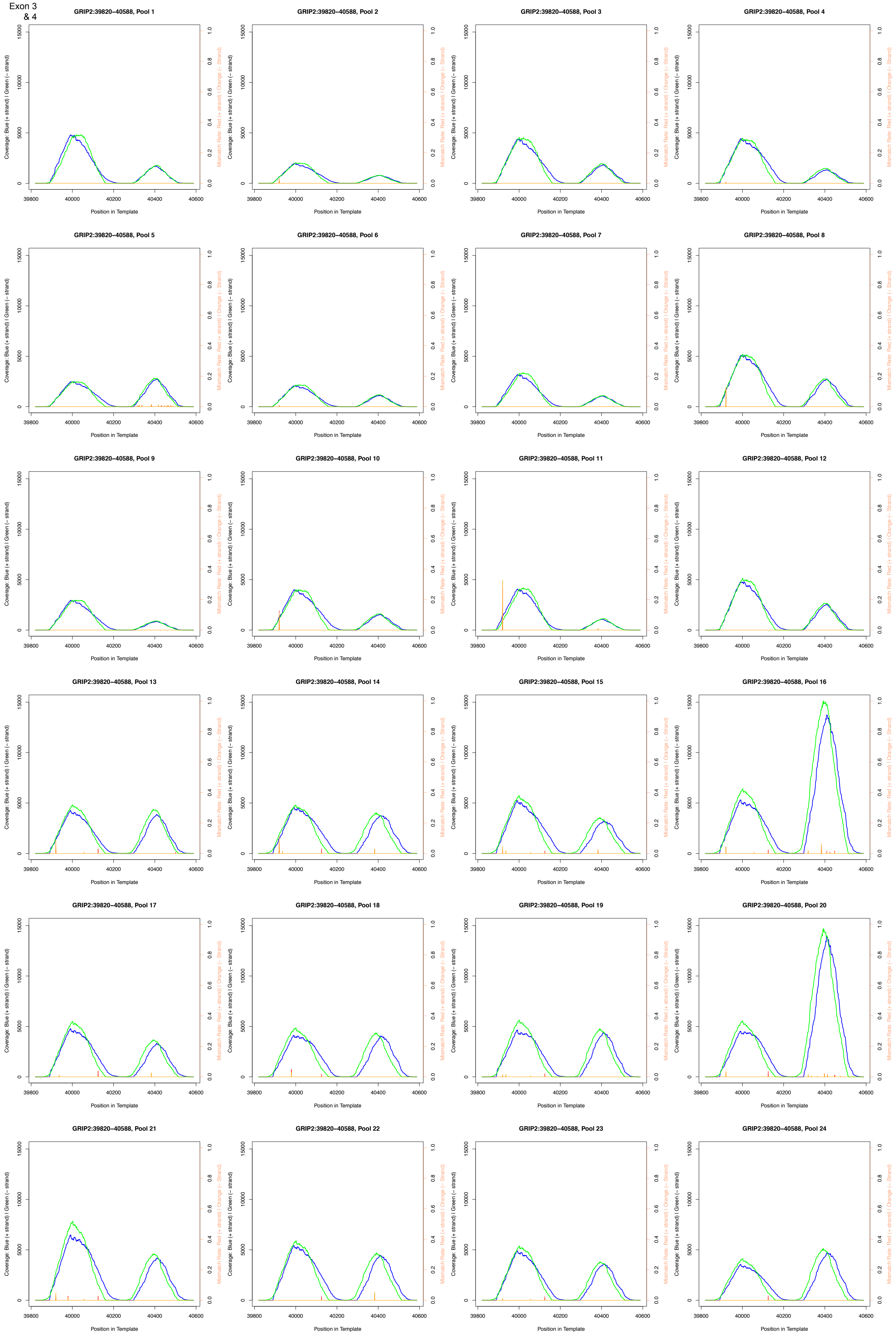
GRIP2:39820–40588, Pool 1
GRIP2:39820–40588, Pool 2
GRIP2:39820–40588, Pool 3
GRIP2:39820–40588, Pool 4
GRIP2:39820–40588, Pool 5
GRIP2:39820–40588, Pool 6
GRIP2:39820–40588, Pool 7
GRIP2:39820–40588, Pool 8
GRIP2:39820–40588, Pool 9
GRIP2:39820–40588, Pool 10
GRIP2:39820–40588, Pool 11
GRIP2:39820–40588, Pool 12
GRIP2:39820–40588, Pool 13
GRIP2:39820–40588, Pool 14
GRIP2:39820–40588, Pool 15
GRIP2:39820–40588, Pool 16
GRIP2:39820–40588, Pool 17
GRIP2:39820–40588, Pool 18
GRIP2:39820–40588, Pool 19
GRIP2:39820–40588, Pool 20
GRIP2:39820–40588, Pool 21
GRIP2:39820–40588, Pool 22
GRIP2:39820–40588, Pool 23
GRIP2:39820–40588, Pool 24
Coverage: Blue (+ strand) | Green (− strand)
Mismatch Rate: Red (+ strand) | Orange (− Strand)
Position in Template

Exon 2

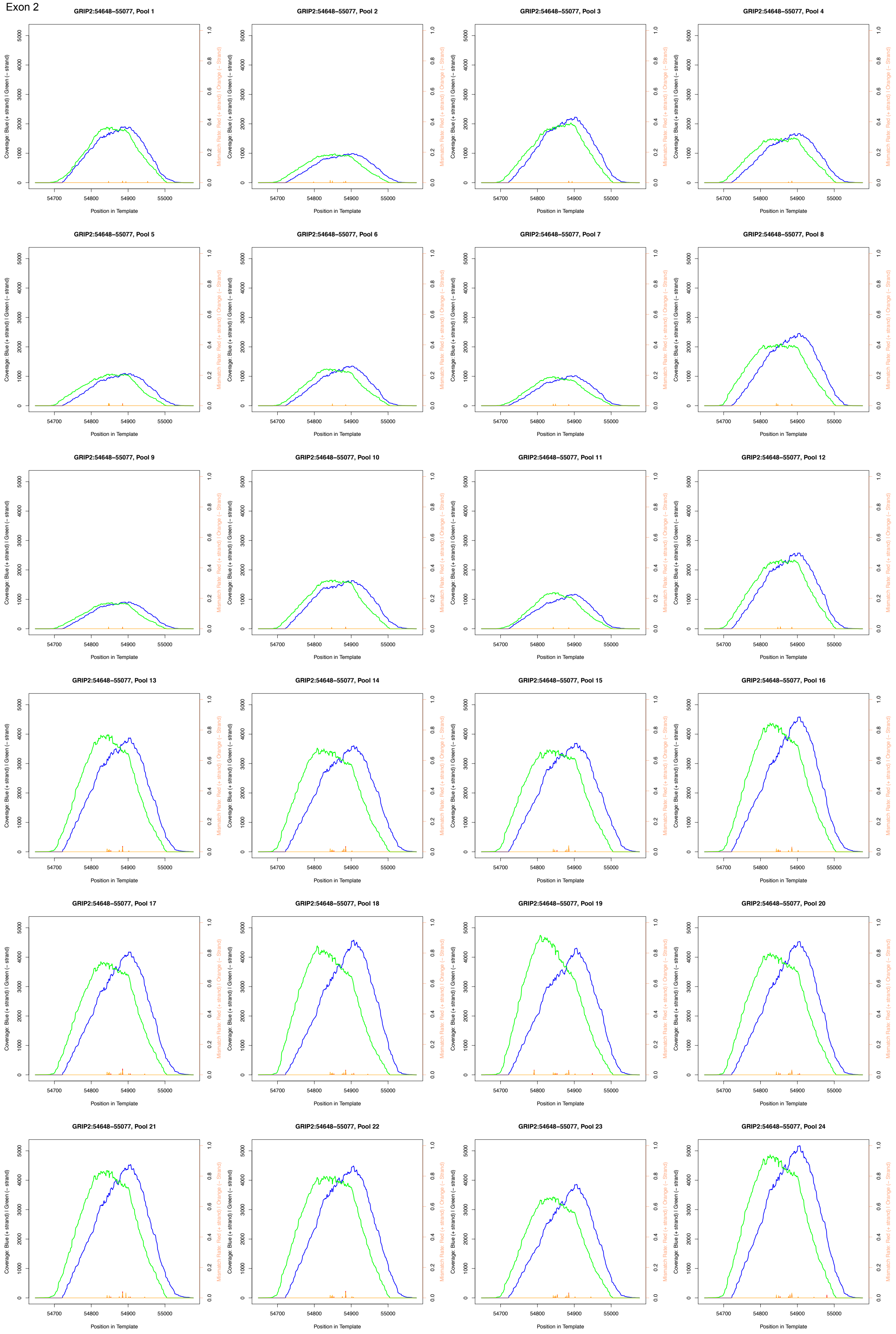
GRIP2:54648–55077, Pool 1
GRIP2:54648–55077, Pool 2
GRIP2:54648–55077, Pool 3
GRIP2:54648–55077, Pool 4
GRIP2:54648–55077, Pool 5
GRIP2:54648–55077, Pool 6
GRIP2:54648–55077, Pool 7
GRIP2:54648–55077, Pool 8
GRIP2:54648–55077, Pool 9
GRIP2:54648–55077, Pool 10
GRIP2:54648–55077, Pool 11
GRIP2:54648–55077, Pool 12
GRIP2:54648–55077, Pool 13
GRIP2:54648–55077, Pool 14
GRIP2:54648–55077, Pool 15
GRIP2:54648–55077, Pool 16
GRIP2:54648–55077, Pool 17
GRIP2:54648–55077, Pool 18
GRIP2:54648–55077, Pool 19
GRIP2:54648–55077, Pool 20
GRIP2:54648–55077, Pool 21
GRIP2:54648–55077, Pool 22
GRIP2:54648–55077, Pool 23
GRIP2:54648–55077, Pool 24
Coverage: Blue (+ strand) | Green (− strand)
Mismatch Rate: Red (+ strand) | Orange (− Strand)
Position in Template
0
1000
2000
3000
4000
5000
0.0
0.2
0.4
0.6
0.8
1.0
54700
54800
54900
55000

Exon 1

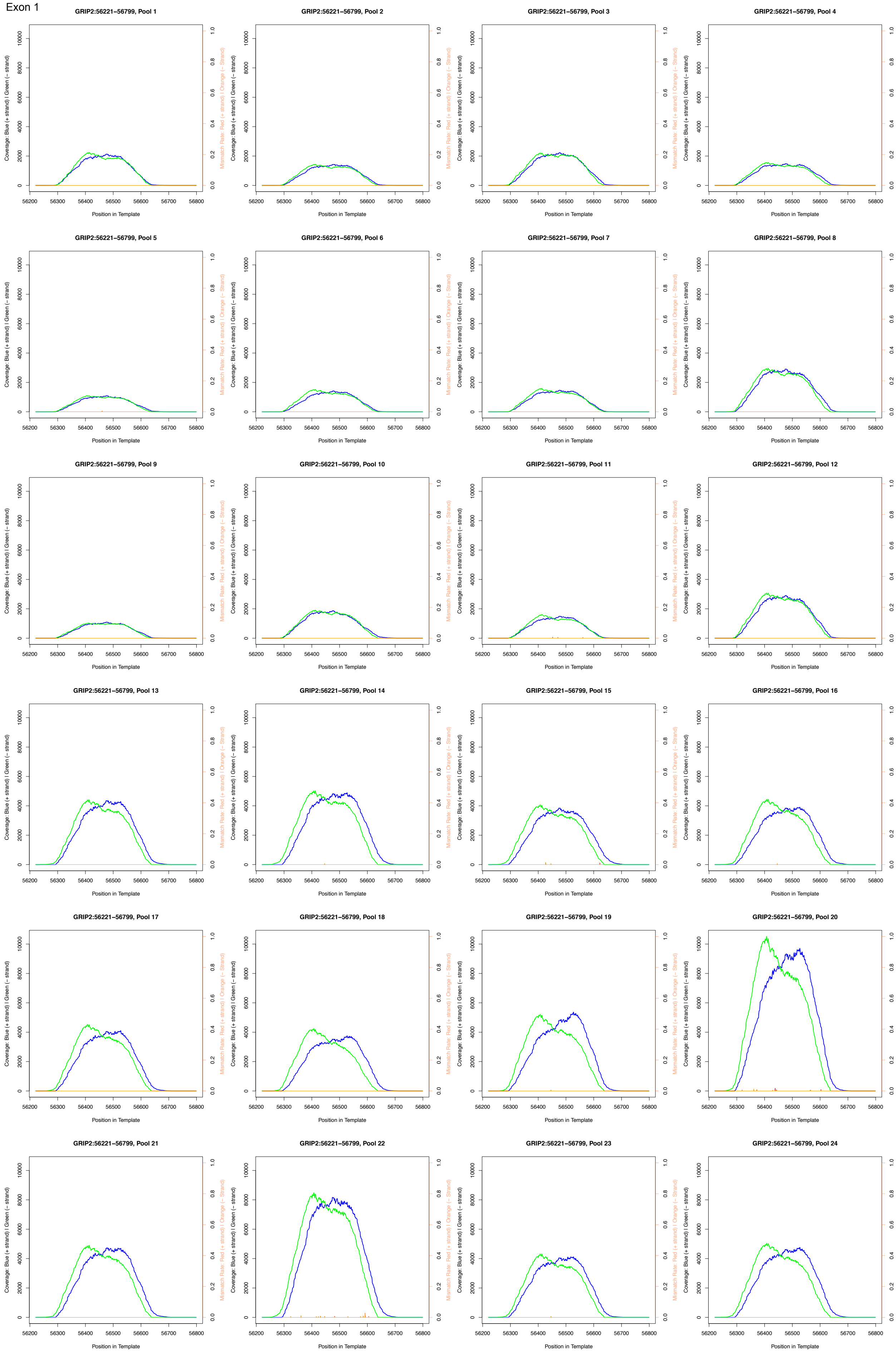
GRIP2:56221–56799, Pool 1
GRIP2:56221–56799, Pool 2
GRIP2:56221–56799, Pool 3
GRIP2:56221–56799, Pool 4
GRIP2:56221–56799, Pool 5
GRIP2:56221–56799, Pool 6
GRIP2:56221–56799, Pool 7
GRIP2:56221–56799, Pool 8
GRIP2:56221–56799, Pool 9
GRIP2:56221–56799, Pool 10
GRIP2:56221–56799, Pool 11
GRIP2:56221–56799, Pool 12
GRIP2:56221–56799, Pool 13
GRIP2:56221–56799, Pool 14
GRIP2:56221–56799, Pool 15
GRIP2:56221–56799, Pool 16
GRIP2:56221–56799, Pool 17
GRIP2:56221–56799, Pool 18
GRIP2:56221–56799, Pool 19
GRIP2:56221–56799, Pool 20
GRIP2:56221–56799, Pool 21
GRIP2:56221–56799, Pool 22
GRIP2:56221–56799, Pool 23
GRIP2:56221–56799, Pool 24
Coverage: Blue (+ strand) | Green (– strand)
Mismatch Rate: Red (+ strand) | Orange (– Strand)
Position in Template
56200 56300 56400 56500 56600 56700 56800
0 2000 4000 6000 8000 10000
0.0 0.2 0.4 0.6 0.8 1.0
