# Supplementary material for: Effective detection of rare variants in pooled DNA samples using Cross-pool tailcurve analysis
Source: Genome Biol. 2011 Sep 28;12(9):R93. doi: 10.1186/gb-2011-12-9-r93 (PMC3308056; doi:10.1186/gb-2011-12-9-r93)
Supplement: Additional file 2 — Description of tailcurve (nucleotide proportion at individual cycles along the sequence read). With perfect random fragmentation, a given position and its associated base calls (consensus and variant) should be represented at multiple sequencing cycles. With high coverage, a particular base call will be present for that position at all or most cycles. Example: for a sequencing module of 25 cycles with several hundred (24 shown) overlapping reads covering the highlighted position, all the cycles are represented by 'G', with variant reads producing the 'T' at a handful of cycles (potential variant). [file gb-2011-12-9-r93-S2.PDF]

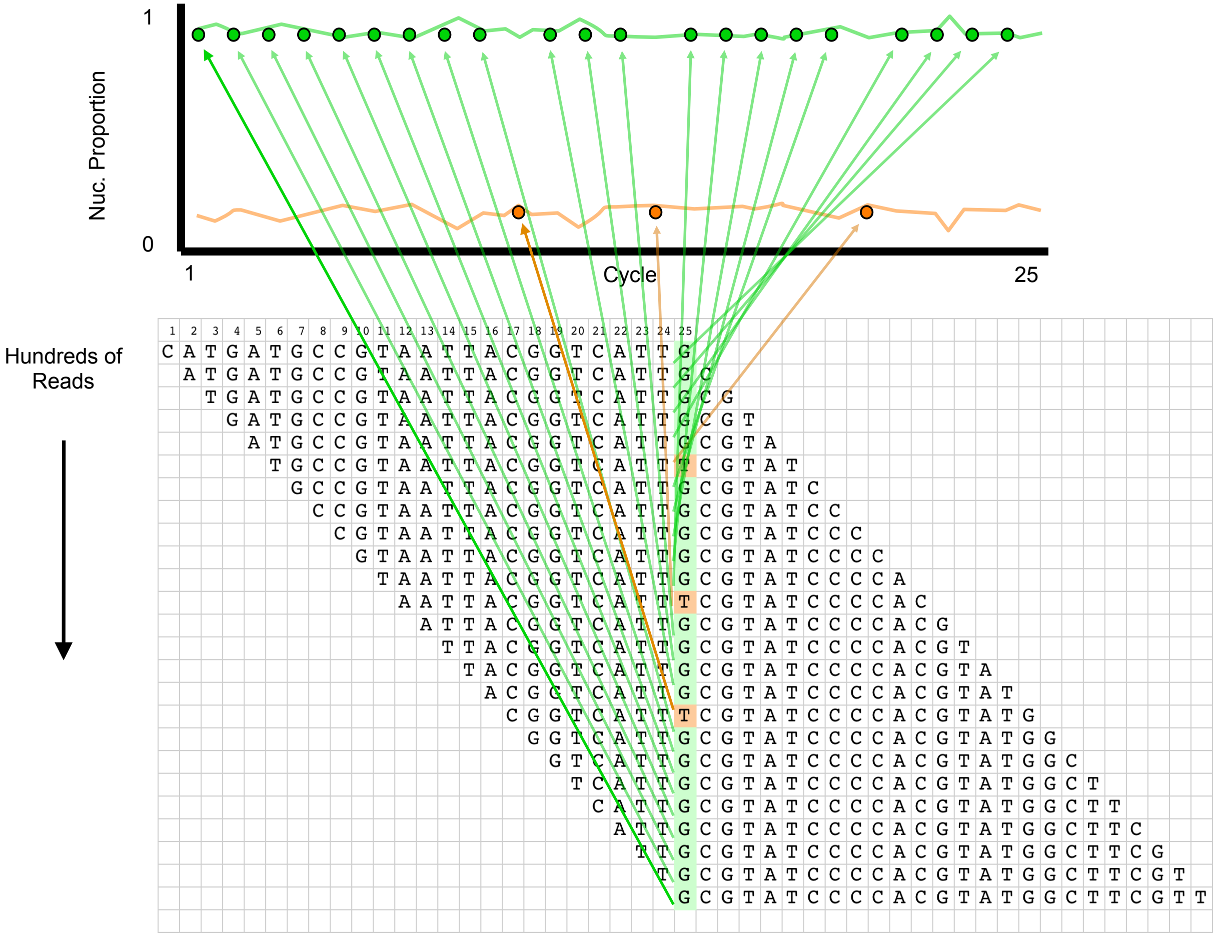
1
0
Nuc. Proportion
1
Cycle
25
Hundreds of Reads
1 2 3 4 5 6 7 8 9 10 11 12 13 14 15 16 17 18 19 20 21 22 23 24 25
C A T G A T G C C G T A A T T A C G G T C A T T G
A T G A T G C C G T A A T T A C G G T C A T T G C
T G A T G C C G T A A T T A C G G T C A T T G C G
G A T G C C G T A A T T A C G G T C A T T G C G T
A T G C C G T A A T T A C G G T C A T T G C G T A
T G C C G T A A T T A C G G T C A T T T C G T A T
G C C G T A A T T A C G G T C A T T G C G T A T C
C C G T A A T T A C G G T C A T T G C G T A T C C
C G T A A T T A C G G T C A T T G C G T A T C C C
G T A A T T A C G G T C A T T G C G T A T C C C C
T A A T T A C G G T C A T T G C G T A T C C C C A
A A T T A C G G T C A T T T C G T A T C C C C A C
A T T A C G G T C A T T G C G T A T C C C C A C G
T T A C G G T C A T T G C G T A T C C C C A C G T
T A C G G T C A T T G C G T A T C C C C A C G T A
A C G G T C A T T G C G T A T C C C C A C G T A T
C G G T C A T T T C G T A T C C C C A C G T A T G
G G T C A T T G C G T A T C C C C A C G T A T G G
G T C A T T G C G T A T C C C C A C G T A T G G C
T C A T T G C G T A T C C C C A C G T A T G G C T
C A T T G C G T A T C C C C A C G T A T G G C T T
A T T G C G T A T C C C C A C G T A T G G C T T C
T T G C G T A T C C C C A C G T A T G G C T T C G
T G C G T A T C C C C A C G T A T G G C T T C G T
G C G T A T C C C C A C G T A T G G C T T C G T T
